# Covalent Organic Framework Colloids With Core‐Shell Donor/Acceptor Architecture and Ultralong‐Lived Charge‐Separated States

**DOI:** 10.1002/anie.8901776

**Published:** 2026-09-01

**Authors:** Ting Yu, Cory Ruchlin, Tong Li, Mohammad Hossein Gohari, Zhechang He, Zeina Chamseddine, Chang Wan Kang, Dongling Ma, Dmytro F. Perepichka

**Affiliations:** ^1^ Department of Chemistry McGill University Montreal Quebec Canada; ^2^ Énergie, Matériaux et Télécommunications Institut National de La Recherche Scientifique (INRS) Varennes Québec Canada

**Keywords:** CO_2_ photoreduction, core‐shell heterojunctions, covalent organic frameworks, long‐lived charge‐separated states

## Abstract

Covalent organic frameworks (COFs) possess structural tunability, high surface area, and extended delocalization of π‐electrons but remain limited by challenges in constructing well‐defined structures that enable efficient charge separation. Here, we address this limitation through the construction of core‐shell colloidal COF particles using isostructural but electronically distinct donor (D) and acceptor (A) COFs. The resulting p‐n heterojunction architecture enables efficient spatial separation of photogenerated electrons and holes, while preserving high crystallinity. Nanoscale phase separation is confirmed by high‐angle annular dark‐field scanning transmission electron microscopy coupled with energy‐dispersive X‐ray spectroscopy (HAADF‐EDS) mapping and X‐ray photoelectron spectroscopy (XPS) analysis. Light‐induced electron paramagnetic resonance (EPR) measurements reveal enhanced populations and significantly longer lifetimes of charge‐separated states in the core‐shell COFs compared to their single‐phase counterparts, indicating suppressed charge recombination and improved charge separation. The resulting heterostructures show efficient CO_2_ photoreduction to CO and HCOOH, coupled with H_2_O oxidation, thereby requiring no sacrificial agents. The results demonstrate the importance of well‐defined D/A interfaces in COF photocatalysis.

## Graphical abstract

Uniform core‐shell colloidal particles of Covalent Organic Frameworks (COFs) are synthesized by a seeded growth of electron‐acceptor COF shells on electron‐donor COF cores. The resulting p‐n heterojunction enables efficient separation of photogenerated charges, which persist for minutes. These core‐shell COFs show efficient CO_2_ photoreduction to CO and HCOOH, coupled with H_2_O oxidation, thereby requiring no sacrificial agents.

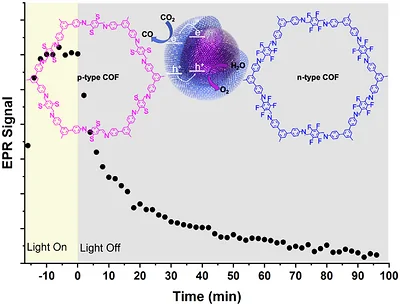

## 1 Introduction

Covalent organic frameworks (COFs) have emerged as a versatile class of crystalline, porous materials constructed by covalent bonding of organic building blocks [1, 2, 3]. Due to the possibility of 2D electron delocalization [4], π‐conjugated COFs have attracted significant interest as organic semiconductors, with potential applications in field‐effect transistors, sensors, photovoltaics, and photocatalysis [3, 5, 6, 7, 8]. Many of these applications, in particular photocatalysis and photovoltaics, require efficient separation and transport of photo‐generated charges. The high surface area and molecularly defined porosity of COF‐based semiconductors provide distinct advantages for photocatalysis by facilitating charge carrier utilization by mass transport to the internal surface. There has been very significant research activity around the use of COFs in photocatalytic H_2_ evolution [8] and CO_2_ reduction [9]. However, the overwhelming majority of reports rely on sacrificial donors to capture photogenerated holes [10, 11, 12, 13]. Overall, achieving efficient charge separation and long‐range transport within COFs remains challenging, and in most semiconducting applications, π‐conjugated COFs are still outperformed by linear conjugated polymers [14, 15].

Two main strategies have been pursued to enhance the photogenerated charge separation in COFs: (i) copolymerization of donor (D) and acceptor (A) moieties forming alternating D‐A COF sheets which could form bulk heterojunctions (BHJ) via segregated stacking [16, 17, 18, 19], and (ii) infiltration of fullerene‐based molecular acceptors (C_60_, PCBM) in the pores of a π‐conjugated COF donor [20, 21, 22]. In both strategies, the size of the donor/acceptor (D/A) domains is defined by the molecular building blocks (∼1 nm), which limits long‐distance charge separation and favors the undesired charge recombination.

Inorganic core‐shell nanoparticles (NPs) with distinct p‐ and n‐type domains in the core and shell regions, respectively, are widely used in photocatalysis because they enable efficient charge separation and feature a large surface‐to‐volume ratio, enabling high reaction rates [23]. However, the monolithic, non‐porous nature of the crystalline inorganic shell effectively blocks access to reactants for one of the photogenerated carriers in the core of the NPs [24]. In contrast, the permeability of the porous COF shells can provide access of reactant molecules to the core, enabling effective utilization of both charge carriers confined in the core and shell domains [25]. Several examples of COF‐based hybrid nanoparticle heterojunctions with inorganic materials (semiconducting metal oxides [26], MOF [27, 28], CdS [29, 30], ZnIn_2_S_4_ [31]_,_ graphene [32]) have been reported. For example, Wang et al. showed that the hollow core‐shell heterojunction structure of TAPB‐COF@ZnIn_2_S_4_ could improve the efficiency of charge separation, leading to higher CO_2_‐to‐CO photocatalytic activity than that of the original ZnIn_2_S_4_ (about 6‐fold) and TAPB‐COF (about 30‐fold) [31]. While encouraging, these reported approaches provide limited structural control of the p/n interface, which complicates the structure‐properties relationships and thus impedes the optimization of such materials.

To our knowledge, only a few studies have reported the synthesis of fully COF‐based heterostructures [33, 34, 35, 36, 37]. Early efforts used post‐synthetic linker exchange to generate compositionally heterogeneous COFs [33, 35]. However, the polydisperse morphology and identical elemental composition of both COFs complicate the analysis of the heterostructure of these COFs. Later, core‐shell imine‐linked COFs with different monomers in the core (terephthalic aldehyde) and the shell (trimesic aldehyde) have been prepared, and the self‐coagulation of the shell was suppressed through the increased concentration of monofunctional competitors (aniline and benzaldehyde) [34]. Also, core‐shell microparticles of non‐conjugated COF‐300 were prepared by controlling the crystalline polymorphs in the core (5‐fold interpenetration) and in the shell (7‐fold interpenetration) through increasing the catalyst concentration during the synthesis [36]. The only report of a COF/COF heterostructure used as a semiconducting heterojunction is the recent work of Zhang and coworkers. The authors used microcrystalline powder of COF‐316 (dioxine‐fused donor COF) as a seed to overgrow TpBpy‐COF (imine‐linked acceptor COF) [37]. Although the polydisperse nature of these COFs makes the characterization of the D/A interface challenging, an efficient overall photosplitting of water was achieved after doping these heterostructures with platinum (Pt). Nonetheless, the construction of core‐shell COF NPs with well‐defined D/A interfaces remains an unsolved challenge. In particular, the lattice mismatch between core and shell COFs, and the limited control over the reaction under solvothermal conditions can lead to ‘self‐coagulation’ of the shell, resulting in the formation of separate particles.

To address this challenge, we employed stable colloidal solutions of COF NPs. An essential element of this approach is the use of acetonitrile as a (co)solvent, which, presumably, stabilizes the charges on the COF surface, suppressing their aggregation [38]. Such stabilization enables control over the COF polymerization/crystallization process, allowing the seeded growth of COF NPs of various sizes [39] and even enables the growth of single‐crystal 2D COFs [40]. To apply this strategy for the synthesis of core‐shell COF heterojunctions, we used isostructural but electronically distinct (D and A) monomers to promote the growth of an electron‐accepting COF shell on the seeds of the electron‐donating COF core with similar lattice parameters.

Here, we report the synthesis of a series of imine‐linked COF NPs (～350–450 nm) with a core‐shell structure and precise control over shell thickness. The formation of separate donor (core) and acceptor (shell) domains is confirmed by high‐angle annular dark‐field scanning transmission electron microscopy coupled with energy‐dispersive X‐ray spectroscopy (HAADF‐EDS) and X‐ray photoelectron spectroscopy (XPS). Photoinduced electron paramagnetic resonance (EPR) spectroscopy confirmed enhanced interfacial charge separation and exceptionally long‐lived (several minutes) charge‐separated states. These core‐shell COF heterojunctions achieved high CO_2_ photoconversion rates under Xe lamp illumination (∼835 mW/cm^2^) producing CO and HCOOH (∼2 mmol h^−1^ g^−1^), along with O_2_ (∼1 mmol h^−1^ g^−1^), without the use of any sacrificial agents.

## 2 Results and Discussion

### 2.1 Synthesis of Spherical COFs

A series of electron‐donating (MeO‐COF, MeS‐COF) and electron‐accepting (TF‐COF, DCl‐COF) COFs were synthesized as spherical NPs by Schiff‐base condensation of corresponding aldehydes and amines (Scheme 1), following Dichtel's method [39, 41]. These substituents allow tuning the electronic properties of the COFs without significantly perturbing their lattice. The polymerization was carried out at 90°C with acetic acid (AcOH) as a catalyst, in a mixed acetonitrile‐benzonitrile solvent (2:1, v/v), and in the presence of aniline as a monofunctional modulator. We found these conditions to be superior to condensation with Sc(OTf)_3_ catalyst in producing well‐defined particles for all studied COFs except the TA‐COF [39] with the terephthalaldehyde linker (Figure S1). The addition of benzonitrile improves the solubility of the monomers (and growing COF oligomers), which is critical for achieving uniform COF morphology. The yellow colloidal solutions, formed within the first few minutes of the reaction, were cooled down and centrifuged to collect the COF precipitate, which was washed with acetone and acetonitrile and re‐dispersed into acetonitrile. We note that the scope of this synthetic approach may be limited by the solubility of building blocks. Thus, the synthesis of electron‐accepting DCl‐COF from the poorly soluble 4,4′,4″‐(1,3,5‐triazine)tribenzaldehyde (Tz‐TBA) required a modification of the procedure: the Tz‐TBA was first solubilized by in situ formation of an imine (heating with aniline modulator and AcOH catalyst) before introducing the diamine monomer.

**SCHEME 1:**
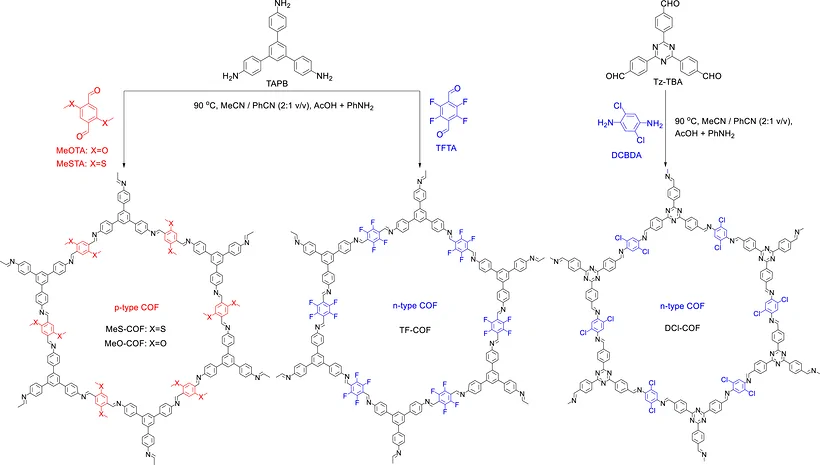
Synthesis and structures of electron‐donating MeO‐COF and MeS‐COF, and electron‐accepting TF‐COF and DCl‐COF.

Scanning Electron Microscopy (SEM) and Transmission Electron Microscopy (TEM) images confirm the formation of well‐defined spherical particles with uniform size (Figure 1a–d). The particle size can be tuned in the ∼300–1200 nm range by controlling the concentration: lower concentration of acetic acid (Figures S2–S4) and higher concentration of the monomers result in larger particles (Figure S6), consistent with a previous report [42]. We also studied the effect of reaction time on the COF growth using SEM and wide‐angle X‐ray scattering (WAXS) (Figures S8, S9). Uniform spherical NPs are already formed after 5 min, but there is a small continuous enhancement of crystallinity up to 1 h reaction time, as indicated by the reduction of the full width at half maximum (FWHM) of the (100) diffraction peak.

**FIGURE 1:**
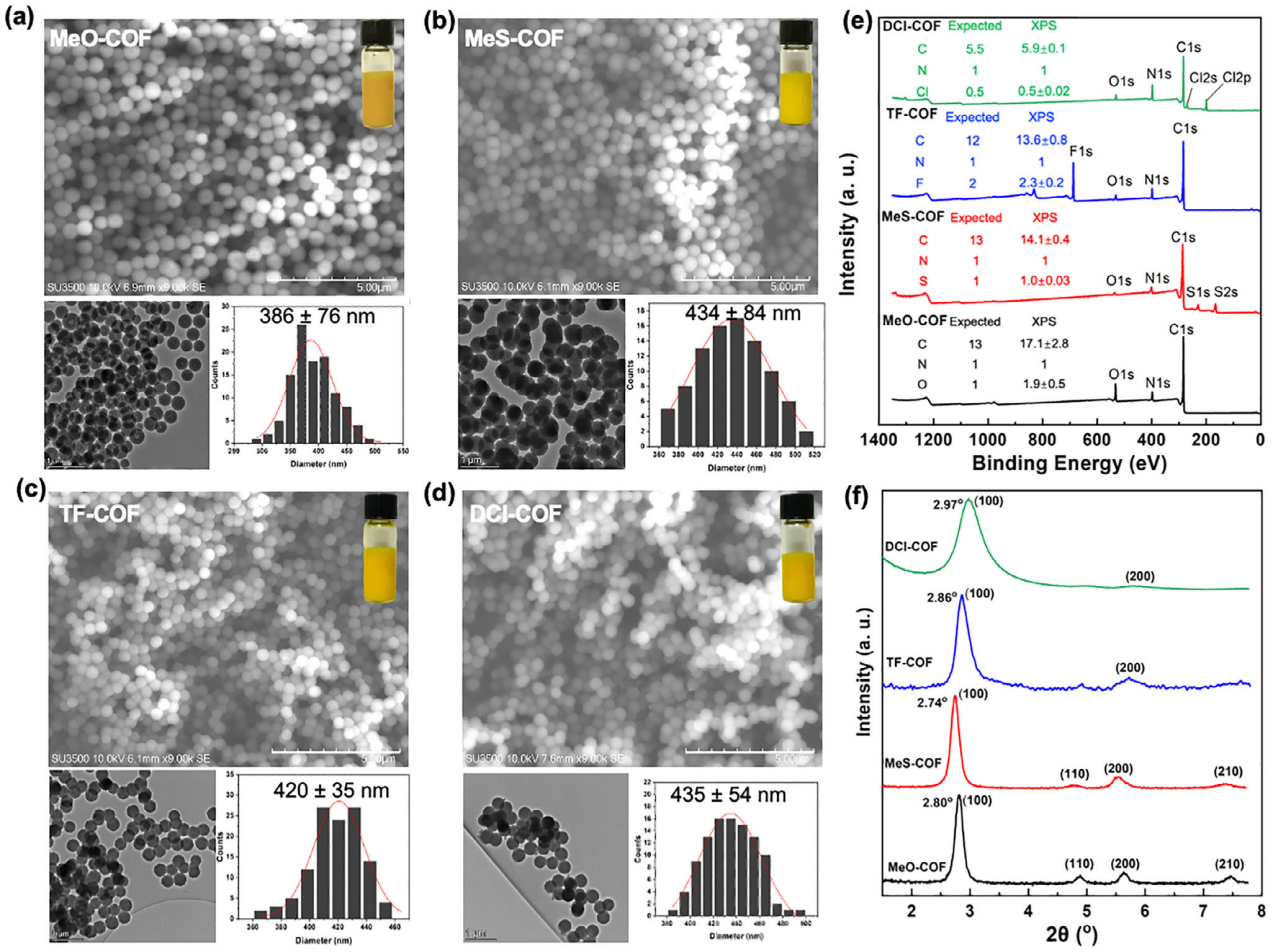
SEM image, TEM image, and particle size histogram of (a) MeO‐COF, (b) MeS‐COF, (c) TF‐COF, and (d) DCl‐COF (the average size is obtained from TEM images with ∼100 particles). (e) XPS spectra (inset: expected and XPS‐derived atomic ratio), and (f) SAXS pattern of MeO‐COF, MeS‐COF, TF‐COF, and DCl‐COF.

### 2.2 Chemical and Structural Characterization

Fourier transform infrared (FT‐IR) spectra confirmed the disappearance of the aldehyde (C═O) stretching of the monomer at ∼1668–1695 cm^−1^, and the emergence of C═N of the COF at ∼1615–1619 cm^−1^ (Figure S14). Solid‐state cross‐polarization magic‐angle spinning ^13^C nuclear magnetic resonance (^13^C CP‐MAS NMR, 11 kHz) spectra confirmed the complete disappearance of the aldehyde signal at ∼190 ppm (Figures S17‐20). XPS reveals the COFs’ composition with the elemental ratio in reasonable agreement with expected values (Figure 1e, Table S2–S5). Deconvolution of high‐resolution C1s, N1s and O1s spectra confirms the presence of the expected functionalities (C═N, C─F, C─O, C─Cl), and also reveals a small amount of unreacted carbonyl group (signals at ∼286.4 eV in the C1s and ∼532 eV in the O1s spectra, Figure S21).

Small‐angle X‐ray scattering (SAXS) measurements show characteristic diffraction peaks for MeO‐COF [43, 44], MeS‐COF [45], and TF‐COF [46], consistent with the previous reports. The previously unreported DCl‐COF also exhibited a similar pattern with reflections at 2𝜃 = 2.97° (100) and 5.79° (200) (Figure 1f). The lowest FWHM of the (100) peak achieved for these spherical COFs was 0.17° for MeO‐COF, 0.18° for MeS‐COF, 0.27° for TF‐COF, and 0.56° for DCl‐COF, which correspond to crystal coherence lengths of ∼50, ∼45, ∼30, and ∼15 nm, respectively. The broader (100) peak (FWHM = 0.56°) of DCl‐COF suggests lower crystallinity, which is likely attributed to the limited solubility of the planar Tz‐TBA monomer, leading to premature aggregation of the growing COF oligomers [47]. Full‐profile Pawley refinement revealed that MeO‐COF, MeS‐COF, TF‐COF, and DCl‐COF possess almost identical 2D unit cells, with in‐plane lattice vectors *a*≈*b*≈37 Å (Figure S15). High‐resolution TEM images of the spherical COFs exhibited well‐defined (100) lattice fringes with radial orientation indicating the polycrystalline nature of the particles (Figure S16).

### 2.3 Electronic Properties of COFs Nanoparticles

UV‐vis diffuse reflectance spectra of the synthesized COF NPs showed very similar absorption optical bandgaps *E*_g_^opt^∼2.5 eV (Figure 2a). However, ultraviolet photoelectron spectroscopy (UPS, Figure S23) shows ∼0.4 eV lower HOMO energy levels for the halogenated TF‐COFs and DCl‐COF compared to MeO‐COF/MeS‐COF (Figure 2b). Density functional theory (DFT, B3LYP/6‐31G(d)) calculations of the COF single layers predict somewhat larger bandgaps (*E*_g_^DFT^) of ∼3.1–3.2 eV, with the same trend in the HOMO energy levels as expected based on the electron‐withdrawing strength of the substituents (∼−5.4 eV for MeO/MeS‐COF, −5.9 eV for TF‐COF, DCl‐COF, Figure 2c). Thus, both the experimental and calculated energy levels show a matched band alignment between p‐type COFs (MeO‐COF and MeS‐COF) and n‐type COFs (TF‐COF and DCl‐COF), suitable for effective charge separation in corresponding heterojunctions.

**FIGURE 2:**
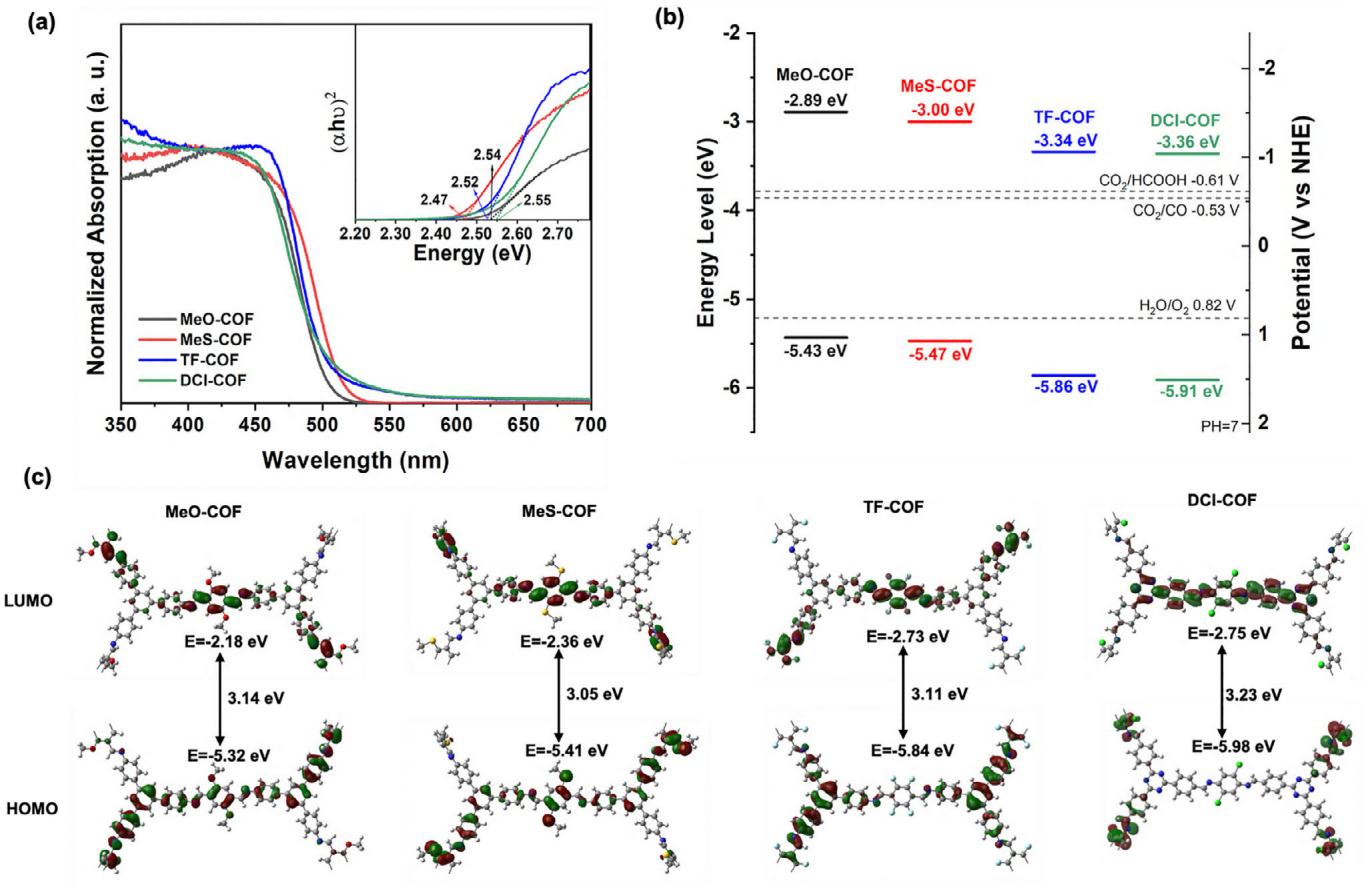
(a) Normalized absorption spectra of COF powders, (b) energy level diagram of COFs (the HOMO energies were determined by UPS, and the LUMO energies were estimated by adding the optical bandgaps obtained from the absorption spectra; the energy levels were converted to the electrochemical potentials (V) assuming NHE at ‐4.44 eV vs vacuum). (c) DFT calculated HOMO/LUMO orbital distribution of MeO‐COF, MeS‐COF, TF‐COF, and DCl‐COF at the B3LYP/6‐31G(d) level of theory.

### 2.4 Synthesis and Characterization of Core‐Shell Heterojunctions

The above experiments showed that uniform spherical COF NPs with nearly identical crystal lattices can be synthesized from monomers with donor and acceptor substituents. We reasoned that the minimal lattice mismatch between COFs with the same framework, but different substituents would facilitate the construction of well‐defined core‐shell COF NPs with a p‐n heterojunction structure for efficient charge separation. To demonstrate the feasibility of this approach, we synthesized core‐shell MeS@TF‐COF heterojunctions by growing the electron‐deficient TF‐COF shell on the electron‐rich MeS‐COF core (∼400 nm) through the seeded‐growth approach [39] (Figure 3a). This specific combination has been selected to enable elemental mapping of the building block distribution within core‐shell particles (see below), but the approach is general, and similar core‐shell MeO@TF COF NPs have also been prepared (Figures S30, 31).

**FIGURE 3:**
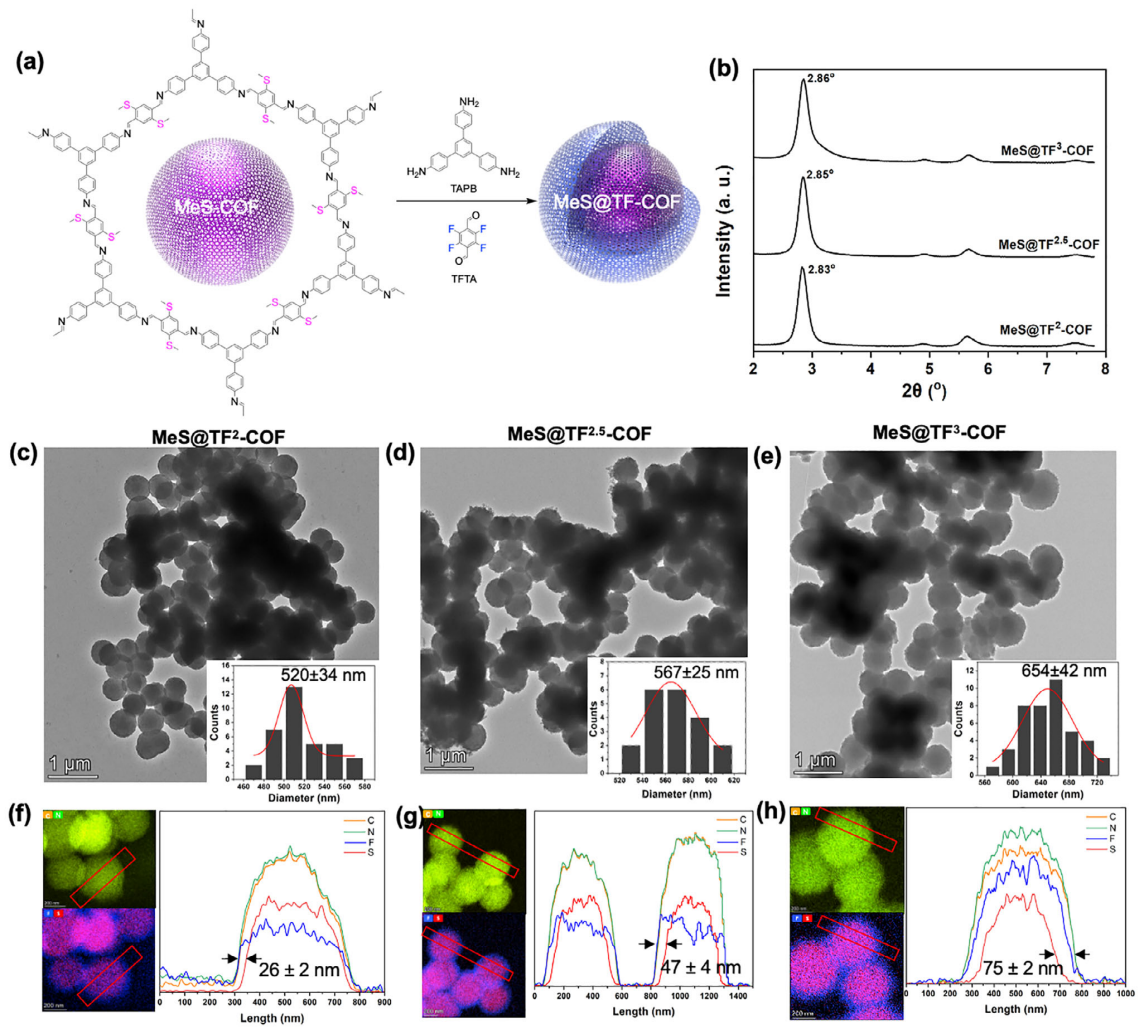
(a) Synthetic scheme of core‐shell MeS@TF‐COF with MeS‐COF as core and TF‐COF as shell. (b) SAXS pattern of core‐shell MeS@TF‐COFs with different shell thickness. (c–e) TEM images and particle size histogram of MeS@TF‐COFs with different shell thickness. (f–h) HAADF‐EDS elemental mappings and related profile lines of (f) MeS@TF^2^‐COF, (g) MeS@TF^2.5^‐COF, and (h) MeS@TF^3^‐COF. The uncertainties (±) were derived from four different positions of EDS elemental mappings.

To grow the shell, separate solutions of TAPB and TFTA monomers (along with AcOH catalyst) were added simultaneously to the above‐described MeS‐COF (particle size ∼430 nm) suspended in the same solvent (MeCN/PhCN) at 90°C. The rate of addition was maintained at 1 equiv. h^−1^ (based on the molar content of the monomers in the MeS‐COF seeds) using a syringe pump, in order to avoid the nucleation of new COF crystals [39]. The shell thickness was tuned by varying the total amount (between 2 and 3 equiv.) of shell monomers added, and the resulting core‐shell particles are labeled according to the number of shell monomer equivalents (e.g., MeS@TF^2.5^ corresponds to 2.5 equiv. of the TFTA vs MeSTA).

SAXS analysis revealed that MeS@TF‐COFs retain high crystallinity with the FWHM_(100)_ of 0.18°–0.20° (Figure 3b), the same as the pristine MeS‐COF core seed particles. As the thickness of the TF‐COF shell increases, the (100) diffraction peak gradually shifts from 2.79° to 2.86°, matching that of the pure TF‐COF (Figure S26d). Brunauer–Emmett–Teller (BET) analysis of N_2_ adsorption‐desorption isotherms of the activated MeS@TF‐COF shows a similar surface area (1497 m^2^ g^−1^) as that of the precursor MeS‐COF NPs (1586 m^2^ g^−1^) (Figure S32).

TEM images reveal the retention of the uniform spherical morphology, with the growing diameter of the particles (Figure 3c–e). High‐resolution TEM images of MeS@TF‐COF acquired from the particle edges exhibited well‐defined lattice fringes, confirming the crystallinity of shell regions (Figure S27). Energy‐dispersive X‐ray spectroscopy (EDS) mapping of F and S elements was used to verify the distribution of the fluorinated and sulfanylated monomers in the core‐shell structure (Figure 3f–h). The cut‐off difference between the F and S profiles in EDS suggests the thickness of TF‐COF shell in core‐shell MeS@TF‐COFs between ∼26 nm and ∼75 nm, depending on the amount of added shell monomers. These thicknesses are lower than what could be expected based on the amount of shell monomer added (98–131 nm). While we cannot exclude some intermixing between the MeS‐COF and TF‐COF in the interfacial region, the major reason for the lower than theoretically expected shell thickness appears to be an equilibrium with the monomers in solution. This is supported by the fact that the TEM‐measured diameter of the obtained core‐shell particle (520–650 nm, Figure 3c–e) is also lower than that expected based on adding 2–3 equiv. of the shell monomers (630–696 nm), especially for the thinner shell particles.

The XPS analysis further confirms the nanoscale phase separation by tracking the evolution of the S and F signals on the surface of COF particles. The characteristic S 2p doublet (163.4 and 164.3 eV) is dramatically suppressed but still observable in the thinnest shell MeS@TF^2^‐COF and becomes undetectable for MeS@TF‐COF with thicker shells (Figure 4a). The F1s/N1s ratios of all three core‐shell COFs are comparable with pristine TF‐COF (Figure 4b). Thus, the growing shell is represented by the pure fluorinated structure.

**FIGURE 4:**
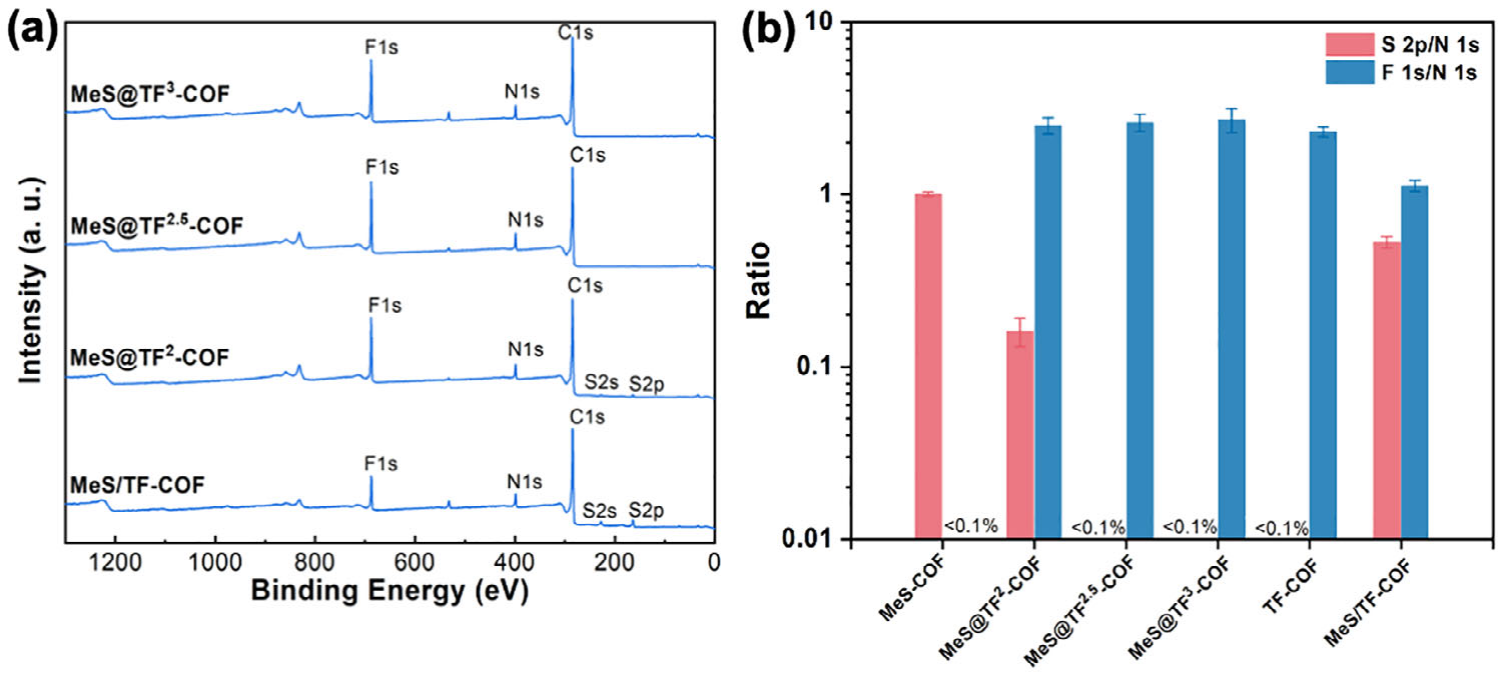
(a) XPS spectra of core‐shell MeS@TF‐COFs with different shell thickness and copolymerized D‐A MeS/TF‐COF, and (b) related F 1s/N 1s and S 2p/N 1s ratio (the higher than expected ∼2.5 F/N ratio in core‐shell structures could be due to the preferential termination of the particle surface with TFTA).

For comparison with core‐shell COF heterojunctions, a mixed donor‐acceptor MeS/TF‐COF was synthesized by copolymerizing MeSTA with TFTA in equimolar amounts using the above synthetic approach. The procedure yielded a similar well‐defined spherical morphology with an average size of ∼593 nm and high crystallinity, with a FWHM_(100)_ of 0.19° (Figures S33, 34). XPS analysis revealed that the main elements are C, N, F, and S for MeS/TF‐COF, and the measured S2p/N1s and F1s/N1s ratios are ∼0.5 and 1.1, in good agreement with the used 1:1 load ratio of the aldehyde linkers (Figure 4a and Table S11).

### 2.5 Charge Separation in Core‐Shell COF NPs

As discussed above, the pristine MeS‐COF and TF‐COF displayed similar absorption spectra with a small ∼0.05 eV red‐shift in the former (Figure 2a). However, the PL emission peak of TF‐COF is red‐shifted compared to MeS‐COF by as much as ∼0.18 eV, likely due to more pronounced charge transfer to the tetrafluoroterephthalic linker in the former (Figures 5a and S38). PL lifetimes of the MeS‐COF and TF‐COF were obtained by time‐correlated single‐photon counting (TCSPC) measurements to aid in understanding their inherent excited state dynamics, and to serve as controls for investigating the exciton dynamics in each phase of the heterogeneous material (Figure 5b). Both component COF materials showed multiexponential decays (typical of solid‐state materials), with the MeS‐COF decay being substantially faster (*τ*_ave_ ∼ 0.8 ± 0.1 ns) than that of the TF‐COF (*τ*_ave_ = 3.1 ± 0.1 ns).

**FIGURE 5:**
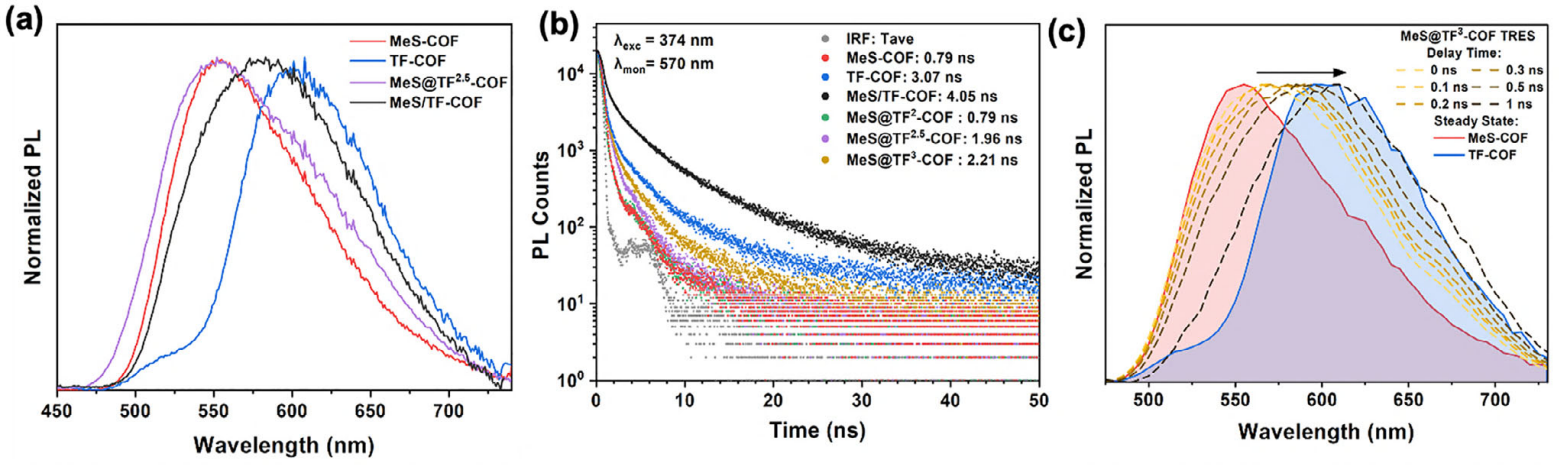
(a) PL spectra (*λ*_exc_ = 380 nm) and (b) PL decay traces of MeS‐COF, TF‐COF, MeS@TF‐COF heterojunctions, and MeS/TF‐COF, monitored at 570 nm. (c) Normalized time‐resolved emission spectra (TRES) spectral cross‐sections of MeS@TF^3^‐COF heterojunctions, tracking change in emission character over time from MeS‐COF‐like to TF‐COF‐like.

The PL spectrum of the copolymerized MeS/TF‐COF presents an emission maximum (581 nm) falling between those of the two pure COFs (554 nm and 605 nm, respectively, Figure 5a). The PL lifetime of MeS/TF‐COF is longer than those of either pure COFs (*τ*_ave_ = 4.0 ± 0.1 ns), likely due to the greater CT character of its transitions having a slower radiative recombination rate (Figure 5b).

In contrast, the steady‐state PL spectra of MeS@TF‐COF are dominated by emission from the MeS‐COF core (Figures 5a and S39). This is expected, given that the core's faster PL lifetime makes it less susceptible to quenching by interfacial charge transfer. Time‐resolved emission spectroscopy (TRES) of MeS@TF^3^‐COF showed a progressive evolution of the luminescence profile: the early emission is more consistent with that of the MeS‐COF core, but within ∼1 ns becomes dominated by the TF‐COF shell (Figure 5c). The PL decay is biexponential, with shorter (*τ*_1_∼0.5 ns) and longer (*τ*_2_∼2.3–2.8 ns) components attributable to the MeS‐COF core and TF‐COF shell, respectively. Monitoring the emission decay at bluer and redder wavelengths shows increased contributions from the shorter and longer lifetime components, respectively (Figure S40). Attributing this trend to diffusion‐limited quenching of mobile excitons at the core‐shell interface, we used these τ_2_ values to calculate the exciton diffusion length (L_D_) through the shell, and estimated L_D_ ∼ 20 nm (Figure S41), which is comparable to the values observed for the broader class of organic semiconductors [48, 49, 50].

Light‐induced EPR spectroscopy was employed to study the charge recombination dynamics in the COF NPs, as has been previously applied to P3HT/PCBM bulk heterojunctions [51]. The evolution of the EPR signal follows the formation and annihilation of radical cations and radical anions via charge separation and charge recombination, respectively. While the light‐induced EPR signal could also arise from excited triplet states, these ought to decay on the sub‐second time scale. We note that a weak EPR signal was observed in core‐shell NPs, even under dark conditions (Figure S42), which may originate from oxidized TAPB with unreacted terminal amino groups (Figure S45).

Under continuous in situ irradiation with a white light source, the EPR signal intensity gradually increases until eventual saturation after 10–20 min (Figure 6a). MeS‐COF and TF‐COF showed a similar increase in spin concentration (measured as athe double integral of the EPR signal, Δ(EDI)), but the mixed MeS/TF‐COF showed several times lower increase (Figures 6b). On the other hand, a 5‐times higher increase was observed for the core‐shell MeS@TF^2.5^‐COF. The spin concentration in the saturation regime is controlled by the competition between charge separation and recombination, and the highest spin concentration in the core‐shell structure suggests a higher ratio of charge separation to recombination. Upon removal of the excitation source, the photoinduced EPR signal persisted for multiple minutes, before gradually returning to the magnitude measured before irradiation. This growth and slow decay of the EPR signal suggest a photoinduced charge separation (also observed by the PL quenching) followed by very slow, diffusion‐limited recombination.

**FIGURE 6:**
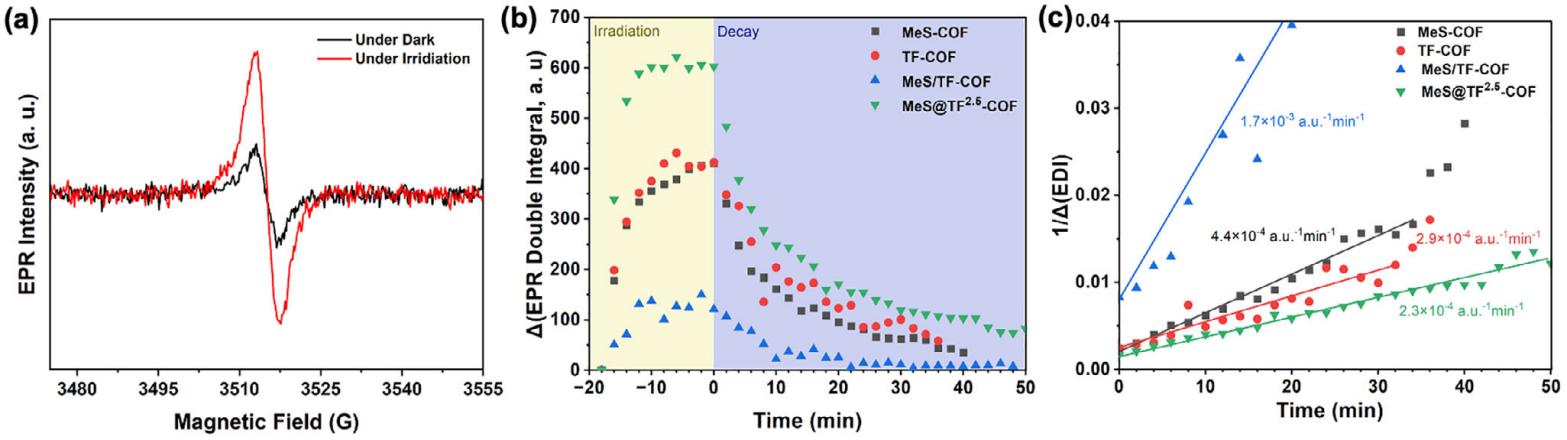
(a) EPR spectrum of MeS@TF^2.5^‐COF in the dark and under irradiation (microwave power: 1.05 mW). (b) Photogenerated EPR signal monitored during irradiation and decay in the dark (g = 2.006). (c) Time‐resolved decay profile obtained by second‐order fit of double integration of MeS‐COF, TF‐COF, MeS/TF‐COF, and MeS@TF^2.5^‐COF.

To address the charge recombination kinetics, we followed the decay of the EPR signal after turning off the light source (Figures 6b and S44). For MeS@TF‐COF, the signal decay is best described by a second‐order (reciprocal) kinetic fit, consistent with the bimolecular recombination of the diffusing holes and electrons (see the comparison of the first‐ and second‐order fits in Figure S44). The decays of MeS‐COF, TF‐COF, and MeS/TF‐COF, while showing a similar behavior, are better fit by the first‐order (exponential) kinetic equations, suggesting unipolar charge trapping by isolated defects in the COFs. The MeS@TF^2.5^‐COF heterojunction showed a lower recombination rate constant (*k*_2_ = 2.3 × 10^−4^ a.u.^−1^ min^‐1^) and longer‐persisting photogenerated EPR signal than the pristine core and shell COFs or the mixed‐phase COF (*k*_2_ = 2.9 × 10^−4^ − 1.6 × 10^−3^ a.u.^−1^ min^‐1^, Figure 6c). To our knowledge, this is one of the longest‐lived charge‐separated states observed in organic heterojunctions [52, 53].

### 2.6 Photocatalytic CO_2_ Reduction

Bulk heterojunction (BHJ) NPs composed of blends of donor and acceptor semiconducting polymers have emerged as efficient photocatalysts for hydrogen evolution [54, 55] and CO_2_ reduction [56]. The nanoscale BHJ morphology promotes exciton dissociation and charge separation, but the non‐porous nature of these materials confines the chemical reactions to the particle surface, and all reported examples with efficient photoreduction using BHJ nanoparticles rely on sacrificial donors (i.e., cannot oxidize water efficiently). On the other hand, the crystalline core‐shell MeS@TF‐COF heterojunctions offer well‐defined donor–acceptor interfaces and long‐lived charge carriers, together with very high, accessible surface area, making them promising platforms for efficient photocatalysis. In addition, the band alignment of the MeS@TF‐COF heterojunction is favorable for photocatalytic CO_2_ reduction (Figure 2b). As a proof‐of‐principle demonstration, the photocatalytic performance of MeS@TF‐COF was investigated in a H_2_O/MeCN (v/v = 1:4) [57] mixed solvent saturated with CO_2_ under irradiation with a 300 W Xe lamp (∼320–780 nm, 835 mW cm^−2^) without any sacrificial donor. The use of acetonitrile as a solvent was dictated by the need to maintain stable colloidal solutions; also, using pure water as a medium accelerates the hydrolysis of these COFs under illumination. Gas chromatographic (GC) analysis of the reactor headspace showed a CO production rate of 0.23 mmol g^−1 ^h^−1^ (Figure 7a), which is very substantial for a system without any sacrificial donor or metal co‐catalysts (Table S13).

**FIGURE 7:**
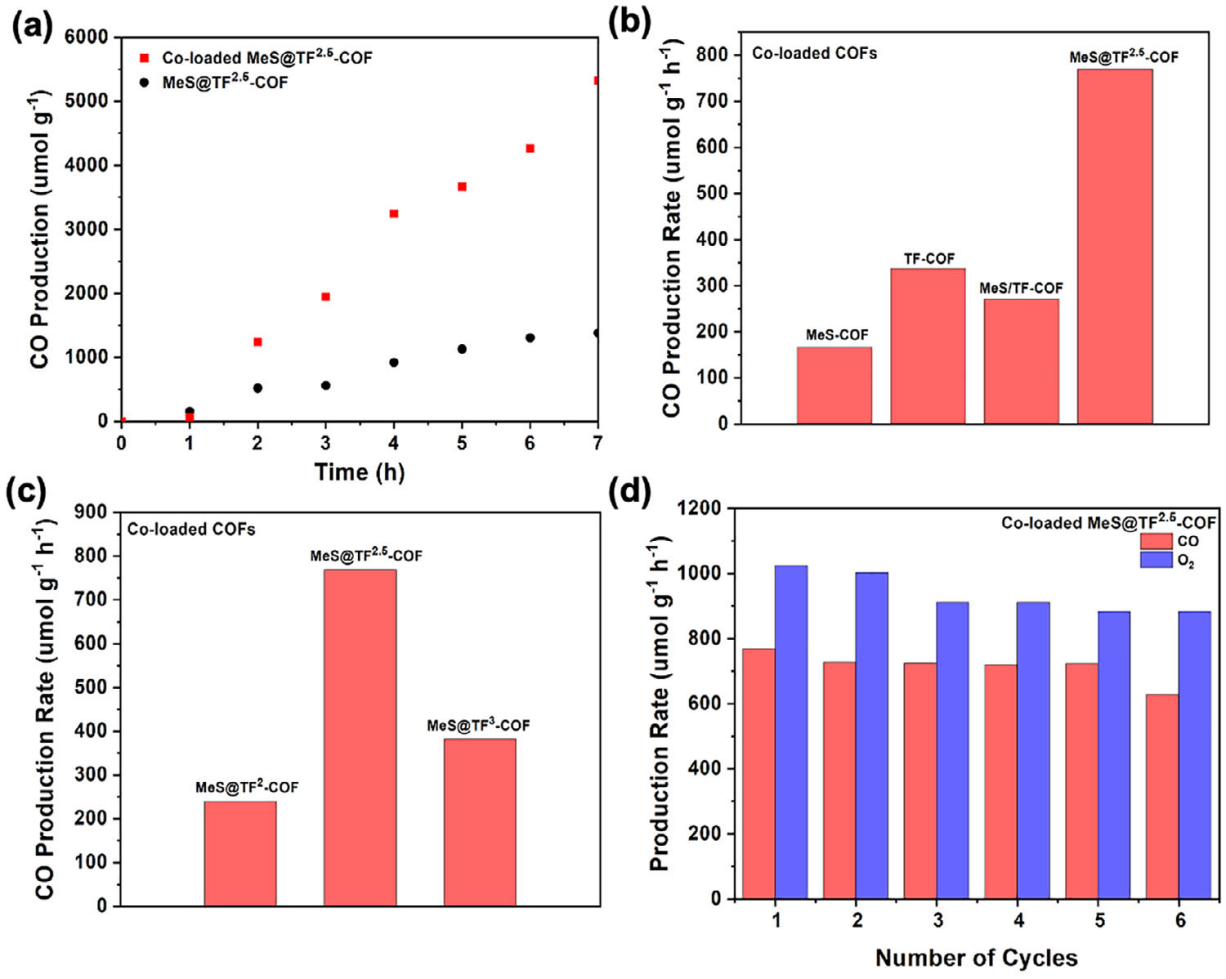
(a) Photocatalytic production rate using [Co(bpy)_3_]^2+^ complex, MeS@TF^2.5^‐COF, and Co‐loaded MeS@TF^2.5^‐COF as photocatalyst. (b) Comparison of photocatalytic production rate for Co‐loaded MeS‐COF, Co‐loaded TF‐COF, Co‐loaded MeS/TF‐COF, and Co‐loaded MeS@TF^2.5^‐COF heterojunctions. (c) Effect of shell thickness of Co‐loaded MeS@TF‐COF on the photocatalytic performance. (d) Cycling stability of the Co‐loaded MeS@TF^2.5^‐COF photocatalyst; each cycle is presented by 5 h of irradiation followed by purging the reaction with CO_2_.

It has been shown that cobalt co‐catalysts can accelerate the photocatalytic CO_2_ reduction, serving as electron‐transfer mediators that bind and activate CO_2_ molecules [58, 59]. The photogenerated electrons in the COF can reduce the Co(II) to Co(I), which then transfers the electron to the coordinated CO_2,_ resulting in its proton‐coupled reduction while regenerating the Co(II) state for continuous turnover. To incorporate the Co co‐catalyst into the COF NPs, MeS@TF^2.5^‐COF was soaked in [Co(bpy)_3_]Cl_2_ solution in methanol, then centrifuged and washed with methanol. This treatment is expected to trap the Co co‐catalyst in the COF pores, possibly aided by coordination to nitrogen atoms. Indeed, inductively coupled plasma optical emission spectrometry (ICP‐OES) of so‐treated COFs detects the presence of ∼0.35 wt% of Co. On the other hand, the XPS surface analysis of these COFs suggests that the amount of Co is below the detection limit (< 0.1 at%), and SEM‐EDX mapping also shows ≤ 0.1 wt% of Co (Figures S51,52). These results suggest that Co is preferentially absorbed in the interior of COF particles rather than on their surface.

The so‐prepared Co‐loaded MeS@TF^2.5^‐COF exhibited a CO production rate of 0.73 mmol g^−1^ h^−1^, a ∼3‐fold enhancement compared to the metal‐free COF photocatalyst. A similar rate of CO production is maintained for at least 7 h of the reaction, except for the apparent “activation period” (slower formation of CO in the first hour), which could be due to photoactivation of the Co catalytic site (Figure 7a). The repeated measurements show a small (∼10%) deterioration of the photocatalytic activity after six 5‐h cycles (Figure 7d). The SEM of the COFs isolated after the reaction shows the retention of their spherical morphology (Figure S53). The gradual loss of efficiency could be, at least in part, due to gradual hydrolysis of the imine links, as suggested by the growing aldehyde stretch in the FTIR spectra (Figure S54) of the COF catalyst isolated after the reaction.

Oxygen evolution is an oxidative counterpart of photocatalytic CO_2_ reduction in the absence of the sacrificial agent. However, the measured CO/O_2_ ratio is smaller than the expected stoichiometry of the reaction (2:1), suggesting a possible formation of an additional reduced product not detected by GC in the reactor headspace. To identify any additional products, the reaction solution was analyzed by ^1^H‐NMR spectroscopy. Indeed, an additional peak at 8.80 ppm characteristic of formic acid was observed, and its quantification yielded a production rate of 1.17 mmol g^−1 ^h^−1^ (Figure S60). The combined rates of formation of the reduction products (CO, 0.73 mmol g^−1 ^h^−1^ and HCOOH, 1.17 mmol g^−1 ^h^−1^) correspond well to that of the oxidation product (O_2_, 1.02 mmol g^−1 ^h^−1^), showing an overall balanced redox reaction (2.32 ×10^21^ electrons g^−1^ h^−1^ vs 2.46 ×10^21^ holes g^−1^ h^−1^).

Co‐loaded MeS‐COF, TF‐COF, and MeS/TF‐COF exhibit the CO production rate of 0.17, 0.34, and 0.27 mmol g^−1 ^h^−1^, which are ∼3 times lower than that of Co‐loaded MeS@TF^2.5^‐COF (Figure 7b). This difference highlights the importance of core‐shell architectures for enhanced photocatalytic performance, arising from efficient spatial charge separation. In addition, the photocatalytic performance of Co‐loaded MeS@TF^2.5^‐COF outperformed those with thinner (26 nm) or thicker shells (75 nm) (Figure 7c). The optimal performance of the medium‐thickness shell NPs is attributed to the competition between charge separation (most effective for the thinnest shell) and charge recombination (most suppressed for the thickest shell), which is expected to lead to the largest amount of long‐lived charge carriers (as observed by EPR, Figure 6).

We also performed control experiments, which revealed no detectable CO formed in the absence of COFs (i.e., with the cobalt complex only), in the absence of CO_2_ (nitrogen atmosphere), or without light irradiation (Figure S55).

## 3 Conclusion

We developed a versatile synthetic strategy to produce uniform core‐shell COF particles. This strategy relies on the previously reported colloidal stabilization of COFs in nitrile‐based solvents, and we showed its generality for a range of imine‐linked donor, acceptor and randomly mixed D‐A COFs (6 examples). Our key innovation is based on lattice matching between the core and shell COFs by employing isostructural but electronically distinct (D/A) monomers. It allows for a uniform growth of the COF shell seeded by (a different) COF core. Structural and compositional analyses by EDS and XPS measurements confirmed the formation of the core‐shell architecture comprising the donor MeS‐COF core and the acceptor TF‐COF shell. EPR measurements revealed more efficient charge separation and slower charge recombination in the core‐shell MeS@TF‐COF architecture relative to either single‐phase (donor or acceptor) COFs or randomly copolymerized MeS/TF‐COF. The ultralong‐lived (minutes) charge population and slow recombination kinetics indicate that the engineered core‐shell p‐n heterojunctions facilitate spatial charge separation and suppress charge recombination, which is essential for exploiting the chemical reactivity of the generated electrons *and* holes. Consequently, MeS@TF‐COF exhibited a remarkable photocatalytic CO_2_‐to‐CO conversion rate among metal‐free COFs without the use of sacrificial donors (0.23 mmol g^−1^ h^−1^), which can be more than tripled upon the addition of cobalt co‐catalyst. The combined production rate of the reduction products (0.73 mmol g^−1^ h^−1^ of CO and 1.17 mmol g^−1^ h^−1^ of HCOOH) is approximately balanced with the oxidation product (1.02 mmol g^−1^ h^‐1^of O_2_). The system shows reasonable stability under the reaction conditions over >30 h of irradiation, although the hydrolysis of the imine‐linked framework occurs in pure aqueous media under irradiation, indicating the need for improved chemical robustness. This work establishes a general and structurally precise approach for engineering COF heterojunctions, in which the relative oxidation/reduction rates and the overall photocatalytic performance can be fine‐tuned by adjusting the shell thickness and/or reversing the p/n core/shell architecture. We expect that the demonstration of porous semiconducting p/n‐heterojunctions based on core‐shell COFs with structurally similar but electronically distinct monomers will stimulate the further development of optoelectronic (e.g., photovoltaic) and photocatalytic applications of these materials.

## Supporting Information

Additional information can be found in the Supporting Information section, including chemical/structural characterization (Figures S1–S16, S21–S36, and S48–S54), absorption/emission data (Figures S37–S40), EPR measurements (Figures S42–S46), and photocatalysis experiments (Figures S55–S60).

## Author Contributions

**Ting Yu**: conceptualization, methodology, investigation, formal analysis, data curation, visualization, writing – original draft, writing – review and editing. **Cory Ruchlin**: investigation, data curation, visualization, writing – review and editing. **Tong Li**: investigation, data curation. **Mohammad Hossein Gohari**: investigation, data curation. **Zhechang He**: investigation. **Zeina Chamseddine**: investigation. **Chang Wan Kang**: visualization. **Dongling Ma**: supervision, writing – review and editing. **Dmytro F. Perepichka**: conceptualization, supervision, writing – review and editing.

## Conflicts of Interest

The authors declare no conflicts of interest.

## Supporting information




**Supporting File**: anie74547‐sup‐0001‐SuppMat.pdf.

## Data Availability

The data that support the findings of this study are available in the Supporting Information of this article and by request from the authors.

## References

[1] A. P. Cote , A. I. Benin , N. W. Ockwig , M. O'Keeffe , A. J. Matzger , and O. M. Yaghi , “Porous, Crystalline, Covalent Organic Frameworks,” Science 310 (2005): 1166–1170, 10.1126/science.1120411. 16293756

[2] K. Geng , T. He , R. Liu , et al., “Covalent Organic Frameworks: Design, Synthesis, and Functions,” Chemical Reviews 120 (2020): 8814–8933, 10.1021/acs.chemrev.9b00550. 31967791

[3] D. Jiang , V. G. W. Tan , Y. Gong , et al., “Semiconducting Covalent Organic Frameworks,” Chemical Reviews 125 (2025): 6203–6308, 10.1021/acs.chemrev.4c00950. 40366230

[4] R. Gutzler and D. F. Perepichka , “π‐Electron Conjugation in Two Dimensions,” Journal of the American Chemical Society 135 (2013): 16585–16594, 10.1021/ja408355p. 24047465

[5] S. Bag , H. S. Sasmal , S. P. Chaudhary , et al., “Covalent Organic Framework Thin‐Film Photodetectors From Solution‐Processable Porous Nanospheres,” Journal of the American Chemical Society 145 (2023): 1649–1659, 10.1021/jacs.2c09838. 36622362

[6] Q. Liu , Q. Li , Y. Li , et al., “Two‐Dimensional Covalent Organic Frameworks in Organic Electronics,” Angewandte Chemie International Edition 64 (2025): e202502536, 10.1002/anie.202502536. 40052756

[7] M. Souto and D. F. Perepichka , “Electrically Conductive Covalent Organic Frameworks: Bridging the Fields of Organic Metals and 2D Materials,” Journal of Materials Chemistry C 9 (2021): 10668–10676, 10.1039/D1TC00750E.

[8] L. Yao , A. M. Pütz , H. Vignolo‐González , and B. V. Lotsch , “Covalent Organic Frameworks as Single‐Site Photocatalysts for Solar‐to‐Fuel Conversion,” Journal of the American Chemical Society 146 (2024): 9479–9492, 10.1021/jacs.3c11539. PMC11009957 38547041

[9] S. Yang , W. Hu , X. Zhang , et al., “2D Covalent Organic Frameworks as Intrinsic Photocatalysts for Visible Light‐Driven CO_2_ Reduction,” Journal of the American Chemical Society 140 (2018): 14614–14618, 10.1021/jacs.8b09705. 30352504

[10] Y. Chen and D. Jiang , “Photocatalysis With Covalent Organic Frameworks,” Accounts of Chemical Research 57 (2024): 3182–3193, 10.1021/acs.accounts.4c00517. 39370855

[11] A. Rodríguez‐Camargo , K. Endo , and B. V. Lotsch , “Celebrating Ten Years of Covalent Organic Frameworks for Solar Energy Conversion: Past, Present, and Future,” Angewandte Chemie International Edition 63 (2024): e202413096, 10.1002/anie.202413096. 39166746

[12] B. Mishra , A. Alam , A. Chakraborty , et al., “Covalent Organic Frameworks for Photocatalysis,” Advanced Materials 37 (2024): 2413118, 10.1002/adma.202413118. PMC12747479 39654345

[13] Y. Zhi , Z. Wang , H. L. Zhang , and Q. Zhang , “Recent Progress in Metal‐Free Covalent Organic Frameworks as Heterogeneous Catalysts,” Small 16 (2020): 2001070, 10.1002/smll.202001070. 32419332

[14] C. Chen , L. Wang , W. Xia , et al., “Molecular Interaction Induced Dual Fibrils Towards Organic Solar Cells With Certified Efficiency Over 20%,” Nature Communications 15 (2024): 6865, 10.1038/s41467-024-51359-w. PMC11316771 39127750

[15] W.‐C. Lin , Y.‐E. Sun , Y.‐R. Zhuang , et al., “Optimally Miscible Polymer Bulk‐Heterojunction‐Particles for Nonsurfactant Photocatalytic Hydrogen Evolution,” Journal of the American Chemical Society 147 (2024): 2537–2548, 10.1021/jacs.4c13856. PMC11760146 39705715

[16] S. Jin , X. Ding , X. Feng , et al., “Charge Dynamics in a Donor–Acceptor Covalent Organic Framework With Periodically Ordered Bicontinuous Heterojunctions,” Angewandte Chemie Intnational Edition 52 (2013): 2017–2021, 10.1002/anie.201209513. 23325571

[17] S. Jin , K. Furukawa , M. Addicoat , et al., “Large Pore Donor–Acceptor Covalent Organic Frameworks,” Chemical Science 4 (2013): 4505, 10.1039/c3sc52034j.

[18] M. Calik , F. Auras , L. M. Salonen , et al., “Extraction of Photogenerated Electrons and Holes From a Covalent Organic Framework Integrated Heterojunction,” Journal of the American Chemical Society 136 (2014): 17802–17807, 10.1021/ja509551m. PMC4706362 25412210

[19] S. Jin , M. Supur , M. Addicoat , et al., “Creation of Superheterojunction Polymers via Direct Polycondensation: Segregated and Bicontinuous Donor–Acceptor π‐Columnar Arrays in Covalent Organic Frameworks for Long‐Lived Charge Separation,” Journal of the American Chemical Society 137 (2015): 7817–7827, 10.1021/jacs.5b03553. 26030399

[20] M. Dogru , M. Handloser , F. Auras , et al., “A Photoconductive Thienothiophene‐Based Covalent Organic Framework Showing Charge Transfer Towards Included Fullerene,” Angewandte Chemie Intnational Edition 52 (2013): 2920–2924, 10.1002/anie.201208514. 23382014

[21] J. Guo , Y. Xu , S. Jin , et al., “Conjugated Organic Framework With Three‐Dimensionally Ordered Stable Structure and Delocalized π Clouds,” Nature Communications 4 (2013): 2736, 10.1038/ncomms3736. PMC3868157 24220603

[22] D. D. Medina , V. Werner , F. Auras , et al., “Oriented Thin Films of a Benzodithiophene Covalent Organic Framework,” ACS Nano 8 (2014): 4042–4052, 10.1021/nn5000223. PMC4762543 24559375

[23] T. Hirakawa and P. V. Kamat , “Charge Separation and Catalytic Activity of Ag@ TiO_2_ Core−Shell Composite Clusters Under UV−Irradiation,” Journal of the American Chemical Society 127 (2005): 3928–3934, 10.1021/ja042925a. 15771529

[24] S. Das , J. Pérez‐Ramírez , J. Gong , et al., “Core–Shell Structured Catalysts for Thermocatalytic, Photocatalytic, and Electrocatalytic Conversion of CO_2_ ,” Chemical Society Reviews 49 (2020): 2937–3004, 10.1039/C9CS00713J. 32407432

[25] X. Wu , M. Sayed , G. Wang , W. Yu , and B. Zhu , “COF‐Based S‐Scheme Heterojunction Photocatalyst,” Advanced Materials 38 (2026): e11322, 10.1002/adma.202511322. 40970807

[26] M. Zhang , M. Lu , Z.‐L. Lang , et al., “Semiconductor/Covalent‐Organic‐Framework Z‐Scheme Heterojunctions for Artificial Photosynthesis,” Angewandte Chemie International Edition 59 (2020): 6500–6506, 10.1002/anie.202000929. 31989745

[27] M. Y. Zhang , J. K. Li , R. Wang , S. N. Zhao , S. Q. Zang , and T. C. Mak , “Construction of Core–Shell MOF@COF Hybrids With Controllable Morphology Adjustment of COF Shell as a Novel Platform for Photocatalytic Cascade Reactions,” Advanced Science 8 (2021): 2101884, 10.1002/advs.202101884. PMC8498909 34378352

[28] J. Wang , Z. Dai , L. Wang , et al., “A Z‐Scheme Heterojunction of Porphyrin‐Based Core–Shell Zr‐MOF@Pro‐COF‐Br Hybrid Materials for Efficient Visible‐Light‐Driven CO_2_ Reduction,” Journal of Materials Chemistry A 11 (2023): 2023–2030, 10.1039/D2TA08333G.

[29] Y. Feng , J. Li , S. Ye , S. Gao , and R. Cao , “Growing COFs In Situ on CdS Nanorods as Core–Shell Heterojunctions to Improve the Charge Separation Efficiency,” Sustainable Energy & Fuels 6 (2022): 5089–5099, 10.1039/D2SE01159J.

[30] Y. Wang , Z. Hu , W. Wang , et al., “Design of Well‐Defined Shell–Core Covalent Organic Frameworks/Metal Sulfide as an Efficient Z‐Scheme Heterojunction for Photocatalytic Water Splitting,” Chemical Science 12 (2021): 16065–16073, 10.1039/D1SC05893B. PMC8672765 35024128

[31] H. Fan , M. Hu , Y. Duan , et al., “Hollow Core–Shell Heterojunction TAPB‐COF@ZnIn_2_S_4_ as Highly Efficient Photocatalysts for Carbon Dioxide Reduction,” Chemical Science 16 (2025): 2316–2324, 10.1039/D4SC07077A. PMC11701727 39776658

[32] Q. Niu , W. Chen , G. Pan , et al., “Spatially Separated Photoredox in a Covalent Organic Frameworks Heterostructure Boosting Overall Water Splitting,” ACS Materials Letters 6 (2024): 1411–1417, 10.1021/acsmaterialslett.4c00026.

[33] G. Zhang , M. Tsujimoto , D. Packwood , et al., “Construction of a Hierarchical Architecture of Covalent Organic Frameworks via a Postsynthetic Approach,” Journal of the American Chemical Society 140 (2018): 2602–2609, 10.1021/jacs.7b12350. 29376387

[34] S. Wang , Y. Yang , P. Liu , et al., “Core‐Shell and Yolk‐Shell Covalent Organic Framework Nanostructures With Size‐Selective Permeability,” Cell Reports Physical Science 1 (2020): 100062, 10.1016/j.xcrp.2020.100062.

[35] Z. Zhang , A. Xiao , C. Yin , X. Wang , X. Shi , and Y. Wang , “Heterostructured Two‐Dimensional Covalent Organic Framework Membranes for Enhanced Ion Separation,” Chemical Communications 58 (2022): 7136–7139, 10.1039/D2CC01749K. 35666182

[36] J. Tang , Y. Chen , T. Gong , and J. Gong , “COF@COF: Constructing Core–Shell Structured Covalent–Organic Frameworks From Interpenetration Isomers,” ACS Macro Letters 12 (2023): 1564–1568, 10.1021/acsmacrolett.3c00624. 37930350

[37] B. B. Luan , X. Chu , Y. Wang , X. Qiao , Y. Jiang , and F. M. Zhang , “Construction of COF/COF Organic S‐Scheme Heterostructure for Enhanced Overall Water Splitting,” Advanced Materials 36 (2024): 2412653, 10.1002/adma.202412653. 39422373

[38] B. J. Smith , L. R. Parent , A. C. Overholts , et al., “Colloidal Covalent Organic Frameworks,” ACS Central Science 3 (2017): 58–65, 10.1021/acscentsci.6b00331. PMC5269657 28149954

[39] R. L. Li , N. C. Flanders , A. M. Evans , et al., “Controlled Growth of Imine‐Linked Two‐Dimensional Covalent Organic Framework Nanoparticles,” Chemical Science 10 (2019): 3796–3801, 10.1039/C9SC00289H. PMC6446964 30996969

[40] A. M. Evans , L. R. Parent , N. C. Flanders , et al., “Seeded Growth of Single‐Crystal Two‐Dimensional Covalent Organic Frameworks,” Science 361 (2018): 52–57, 10.1126/science.aar7883. 29930093

[41] A. Natraj , I. R. Landman , C. E. Pelkowski , D. W. Burke , S. Kewalramani , and W. R. Dichtel , “Nonclassical Crystallization Processes of Single‐Crystalline Two‐Dimensional Covalent Organic Frameworks,” Journal of the American Chemical Society 146 (2024): 16775–16786, 10.1021/jacs.4c04674. 38843538

[42] W. Ma , Q. Zhang , Y. He , et al., “Size‐controllable synthesis of uniform spherical covalent organic frameworks at room temperature for highly efficient and selective enrichment of hydrophobic peptides,” Journal of the American Chemical Society 141 (2019): 18271–18182. 10.1021/jacs.9b09189 31656073

[43] H. Xu , J. Gao , and D. Jiang , “Stable, Crystalline, Porous, Covalent Organic Frameworks as a Platform for Chiral Organocatalysts,” Nature Chemistry 7 (2015): 905–912. 10.1038/nchem.2352 26492011

[44] R. Yuan , Y. He , B. Tang , and H. He , “Enzyme‐immobilized Spherical Covalent Organic Frameworks as Nanoreactors for Heterogeneous Biocatalysis,” CrystEngComm 25 (2023): 3604–3608, 10.1039/D3CE00274H.

[45] N. Huang , L. Zhai , H. Xu , and D. Jiang , “Stable Covalent Organic Frameworks for Exceptional Mercury Removal From Aqueous Solutions,” Journal of the American Chemical Society 139 (2017): 2428–2434, 10.1021/jacs.6b12328. 28121142

[46] Q. Liao , C. Ke , X. Huang , et al., “Catalyst‐Free and Efficient Fabrication of Highly Crystalline Fluorinated Covalent Organic Frameworks for Selective Guest Adsorption,” Journal of Materials Chemistry A 7 (2019): 18959–18970, 10.1039/C9TA06214A.

[47] Y. Che , T. Yu , Z. He , et al., “Highly Crystalline Semiconducting Tetrathienonaphthalene‐Based Covalent Organic Frameworks With Oblique Lattice,” Chemistry of Materials 38 (2026): 7790–7801, 10.1021/acs.chemmater.6c00918.

[48] J. D. A. Lin , O. V. Mikhnenko , J. Chen , et al., “Systematic Study of Exciton Diffusion Length in Organic Semiconductors by Six Experimental Methods,” Materials Horizons 1 (2014): 280–285, 10.1039/C3MH00089C.

[49] Y. Firdaus , V. M. Le Corre , S. Karuthedath , et al., “Long‐Range Exciton Diffusion in Molecular Non‐Fullerene Acceptors,” Nature Communications 11 (2020): 5220, 10.1038/s41467-020-19029-9. PMC7562871 33060574

[50] M. T. Sajjad , A. Ruseckas , L. K. Jagadamma , Y. Zhang , and I. D. Samuel , “Long‐Range Exciton Diffusion in Non‐Fullerene Acceptors and Coarse Bulk Heterojunctions Enable Highly Efficient Organic Photovoltaics,” Journal of Materials Chemistry A 8 (2020): 15687–15694, 10.1039/D0TA06017H.

[51] E. A. Lukina , M. N. Uvarov , and L. V. Kulik , “Charge Recombination in P3HT/PC70BM Composite Studied by Light‐Induced EPR,” Journal of Physical Chemistry C 118 (2014): 18307–18314, 10.1021/jp502299c.

[52] R. Kabe and C. Adachi , “Organic Long Persistent Luminescence,” Nature 550 (2017): 384–387, 10.1038/nature24010. 28967911

[53] B. Cai , A. Brnovic , M. V. Pavliuk , et al., “Organic Crystalline Nanoparticles With a Long‐Lived Charge‐Separated State for Efficient Photocatalytic Hydrogen Production,” Nature Chemistry 18 (2026): 723–730, 10.1038/s41557-025-02035-z. PMC13061632 41559419

[54] J. Kosco , S. Gonzalez‐Carrero , C. T. Howells , et al., “Generation of Long‐Lived Charges in Organic Semiconductor Heterojunction Nanoparticles for Efficient Photocatalytic Hydrogen Evolution,” Nature Energy 7 (2022): 340–351, 10.1038/s41560-022-00990-2.

[55] J. Kosco , M. Bidwell , H. Cha , et al., “Enhanced Photocatalytic Hydrogen Evolution From Organic Semiconductor Heterojunction Nanoparticles,” Nature Materials 19 (2020): 559–565, 10.1038/s41563-019-0591-1. PMC7558859 32015530

[56] M. Ferree , J. Kosco , N. Alshehri , et al., “Organic Semiconductor Nanoparticles for Visible‐Light‐Driven CO_2_ Conversion,” Sustainable Energy & Fuels 8 (2024): 2423–2430, 10.1039/D4SE00196F.

[57] Q. Zhang , S. Gao , Y. Guo , et al., “Designing Covalent Organic Frameworks With Co‐O_4_ Atomic Sites for Efficient CO_2_ Photoreduction,” Nature Communications 14 (2023): 1147, 10.1038/s41467-023-36779-4. PMC9975230 36854683

[58] J.‐M. Lehn and R. Ziessel , “Photochemical Generation of Carbon Monoxide and Hydrogen by Reduction of Carbon Dioxide and Water Under Visible Light Irradiation,” Proceedings of the National Academy of Sciences 79 (1982): 701–704, 10.1073/pnas.79.2.701. PMC345815 16593151

[59] Z. Fu , C. Shu , X. Wang , et al., “Fluorinated Covalent Organic Frameworks Coupled With Molecular Cobalt Cocatalysts for Efficient Photocatalytic CO_2_ Reduction,” CCS Chemistry 5 (2023): 2290–2300, 10.31635/ccschem.023.202202688.

