# Supplementary material for: Covalent Organic Framework Colloids With Core‐Shell Donor/Acceptor Architecture and Ultralong‐Lived Charge‐Separated States
Source: Angew Chem Int Ed Engl. 2026 Sep 1;65(43):e8901776. doi: 10.1002/anie.8901776 (PMC13676409; doi:10.1002/anie.8901776)
Supplement: Supplementary file 1 [file ANIE-65-e8901776-s001.pdf]

Covalent Organic Framework Colloids with Core-Shell Donor/Acceptor Architecture and Ultralong-Lived Charge-Separated States

Ting Yu,[1] Cory Ruchlin,[1] Tong Li,[2] Mohammad Hossein Gohari,[1] Zhechang He,[1] Zeina Chamseddine,[1] Chang Wan Kang,[1] Dongling Ma,*[2] and Dmytro F. Perepichka*[1]

[1] Department of Chemistry, McGill University, Montreal, Quebec, Canada, H3A 0B8,
[2] Énergie, Matériaux et Télécommunications, Institut National de la Recherche Scientifique (INRS), 1650 Boul. Lionel-Boulet, Varennes, Québec, Canada, J3X 1P7

Correspondence:

Dmytro F. Perepichka (dmytro.perepichka@mcgill.ca), Dongling Ma (dongling.ma@inrs.ca)

Table of Content

1. Chemical and General Methods S2
2. Synthetic Procedures S4
3. Characterization of Spherical COF NPs S7
4. DFT Calculations of NMR Chemical Shifts S16
5. XPS/UPS Data S20
6. Characterization of Core-Shell and Mixed D/A COFs S29
7. Optical Characterization of COF NPs S36
8. EPR Characterization of COF NPs S39
9. Photocatalytic CO_2 Reduction Experiments S42
10. Reference S51

1. Chemical and General Methods

Chemicals: All chemicals and reagents were used without further purification. 1,3,5-Tris(4-aminophenyl)benzene (TAPB, ≥95%), 2,5-dimethoxyterephthalaldehyde (MeOTA, 98%), 2,5-bis(methylthio)terephthalaldehyde (MeSTA, 98%), 2,3,5,6-tetrafluoroterephthalaldehyde (TFTA, 96%), 2,5-dichlorobenzene-1,4-diamine (DCBDA, >98%), 4,4',4''-(1,3,5-triazine-2,4,6-triyl)tribenzaldehyde (Tz-TBA, 95%), terephthalaldehyde (TA, 98%) were purchased from A2B Chem.

General Method:

Solid-state NMR spectra were recorded using a Varian VNMRS 400 MHz spectrometer equipped with a 4 mm Varian Chemagnetics T3 probe at a spinning frequency of 11 kHz.

FTIR spectra of powders were recorded using a Nicolet 6700 FT-IR spectrometer in ATR mode in the 600–4000 cm^{-1} region.

SAXS and WAXS patterns were recorded using an Anton Paar SAXSpoint 2.0 under nickel-filtered CuKα (λ = 1.54056 Å) source. COF suspension in acetonitrile was loaded into 1 mm capillary sample holder.

WAXS refinements were performed using the Reflex module in the Materials Studio 2020, in which the Thompson-Cox-Hastings profile function was used for the profile fitting, with all FWHM parameters (U, V, X, Y) and line shift allowed to vary. A medium convergence was first utilized before ultra-fine convergence was applied repetitively. Peak asymmetry was corrected for using the Rietveld correction. Crystallite size and lattice strain broadenings were also refined.

UV-Vis diffuse reflectance spectra of COF powder were ground with KBr and the spectra were recorded using an Agilent Cary-5000 spectrometer.

Steady-state photoluminescence (PL) spectra of COF suspensions in acetonitrile were recorded using a Varian Cary Eclipse fluorescence spectrometer. Time-resolved PL decay measurements were performed by Time-Correlated Single Photon Counting (TCSPC) using a Horiba Scientific system equipped with a DeltaDiode pulsed laser (λ_{exc} = 374 nm) and controlled by a DeltaHub controller. The emission decay was monitored at 570 nm. Time-Resolved Emission Spectra (TRES) were collected by integrating cross-sections of TCSPC traces taken with a constant exposure time (15 seconds) over each wavelength in the scan range. The integrated slices were then normalized for clarity.

Sample preparation for DLS measurement: The as-synthesized COFs were dispersed in acetonitrile to form a very dilute colloidal suspension (ca. 10^{-2} mg/mL). The suspensions were sonicated for 10 min to ensure homogeneous dispersion. The DLS measurements were performed at 60 °C, after ~ 2 min temperature equilibration, to avoid aggregation (which was observed in room temperature measurements).

TEM measurements were conducted using a Thermo Scientific Talos F200X (S)TEM (200 kV). TEM samples were prepared by drop-casting COF suspensions in acetonitrile onto carbon-coated TEM grids. The particle sizes are obtained from average values of over 100 particles in TEM image.

SEM measurements were carried out with Hitachi SU3500 variable pressure-SEM equipped with Oxford SDD EDS and EBSD. SEM samples were prepared by drop-casting COF suspensions in acetonitrile on carbon tape.

X-ray Photoelectron Spectroscopy (XPS) and **Ultraviolet Photoelectron Spectroscopy (UPS)** spectra were obtained with a Thermo scientific spectrometer (Nexsa G2) equipped with an Al Kα monochromatic source and flood gun for charge compensation at base pressures less than 10^{-8} Torr. All binding energies were referenced according to sp^2 C=C at ~284 eV

Density functional theory (DFT) calculations were performed using as implemented in the Gaussian 16 program. Molecular geometries were fully optimized at the B3LYP/6-31G(d) level of theory. Harmonic vibrational frequency calculations were subsequently carried out at the same level to confirm that the optimized structures correspond to true minima on the potential energy surface (no imaginary frequencies).

EPR measurements were conducted using a Bruker X-band EPR spectrometer (Elexsys-680). The spectra were recorded using a modulation amplitude of 1 G and different microwave power to check for signal saturation (see Figure S42). **Light-induced EPR experiments** were carried out using a Fiber-Lite A-200 high-intensity illuminator with the power of 35 mW cm^{-2} (Dolan-Jenner Industries Inc.), and the EPR spectra were continuously recorded (2 min per scan). Sample preparation for EPR measurements was carried out inside a nitrogen-filled glovebox. The COFs were dispersed in toluene (~10 mg/mL) prior to loading into a quartz EPR tube (diameter 4 mm).

The specific surface area of the activated COFs (40 ºC under 10^{-3} Torr for 30 min) was determined from N_2 adsorption isotherms using the multipoint Brunauer–Emmett–Teller (BET) method over a relative pressure (P/P_0) range of 0.05-0.30. BET surface areas were calculated using the BETSI software. Prior to activation, COFs were solvent-exchanged with low-surface-tension solvents (first acetone, then pentane). Direct activation of the COF prepared in MeCN solvent leads to pore collapse resulting in 5-10 times smaller measured surface areas.[1]

2. Synthetic Procedures

Synthesis of MeO-COF Nanoparticles: A vial was charged with MeOTA (14.0 mg, 0.072 mmol) in 12 mL of a mixed acetonitrile/benzonitrile (2:1, v/v) solvent. The mixture was heated in 90 ºC oil bath until the MeOTA was completely dissolved. Then, glacial acetic acid (0.60, 1.2, and 1.8 mL, corresponding to 5, 10, and 15 v/v%, respectively), aniline (13.7 µl, 0.15 mmol, density: 1.02 g mL^{-1}) and TAPB solution (16.9 mg, 0.050 mmol) were subsequently added. The mixture was kept at 90 ºC in an oil bath for 1h without stirring. After completion of the reaction, the mixture was allowed to cool naturally to room temperature. The obtained yellow colloid was centrifuged at 7000 rpm for 10 min, the supernatant solution was decanted, the precipitate was washed with acetonitrile, acetone and methanol (by re-dispersing and centrifugation), and re-dispersed into acetonitrile. Note that drying of COF particles results in partially irreversible aggregation.

Synthesis of MeS-COF Nanoparticles: MeS-COF was prepared following the procedure described above for MeO-COF, using MeSTA (14.0 mg, 0.060 mmol) and TAPB (14.5 mg, 0.040 mmol) monomers. The volume of glacial acetic acid (0.50, 1.0, 1.5 and 2.0 mL, corresponding to 5, 10, 15 and 20 v/v%, respectively) was added to tune the particle morphology and size of MeS-COFs (see Figure S4).

Synthesis of TF-COF Nanoparticles: TF-COF was prepared following the procedure described above for MeO-COF, using TFTA (12.4 mg, 0.060 mmol) and TAPB (13.7 mg, 0.040 mmol) monomers. The volume of glacial acetic acid (0.50, 1.0, 1.5 and 2.0 mL, corresponding to 5, 10, 15 and 20 v/v%, respectively) was added to tune the particle morphology and size of TF-COFs (see Figure S3).

Synthesis of DCl-COF Nanoparticles: A vial was charged with Tz-TBA (15.9 mg, 0.040 mmol) in a 6.4 mL of acetonitrile and benzonitrile (2:1, v/v) mixed solvent. The mixture was heated in 90 ºC oil bath until the Tz-TBA was partially dissolved. Then, acetic acid (1.3 mL) and aniline (9.9 µl, 0.11 mmol) were then added, resulting in the complete dissolution of Tz-TBA. Subsequently, a slight excess of DCBDA (13.8 mg, 0.078 mmol) was added to promote the formation of uniform spherical COF NP, as it facilitates the rapid and homogeneous nucleation under aldehyde-limited condition. The mixture was kept at 90 ºC for 1h without shaking or stirring. After completion of the reaction, the mixture was allowed to cool naturally to room temperature. The obtained yellow colloid was centrifuged at 7000 rpm for 10 min, the supernatant solution was decanted, the precipitate was washed with acetonitrile, acetone and methanol, and re-dispersed into acetonitrile.

Synthesis of MeO/TF-COF Nanoparticles: A vial was charged with MeOTA (11.3 mg, 0.060 mmol) and TFTA (12.0 mg, 0.060 mmol) in 20 mL of acetonitrile and benzonitrile (2:1, v/v) mixed solvent. The mixture was heated in 90 ºC oil bath until the monomers were completely dissolved. Then, acetic acid (3.0 mL), aniline (21.9 µl, 0.240 mmol) and TAPB (27.2 mg, 0.080 mmol) were subsequently added. The mixture was kept at 90 ºC in an oil bath for 1h without any shaking or stirring. After completion of the reaction, the mixture was allowed to cool naturally to room temperature. The obtained yellow MeO/TF-COF colloid was centrifugated at 7000 rpm for 10 min, the supernatant solution was decanted, the precipitate was washed with acetonitrile, acetone and methanol, and re-dispersed into acetonitrile.

Synthesis of MeS/TF-COF Nanoparticles: A vial was charged with MeSTA (13.6 mg, 0.0600 mmol) and TFTA (12.0 mg, 0.0600 mmol) in 20 mL of acetonitrile and benzonitrile (2:1, v/v) mixed solvent. The mixture was heated in 90 ºC oil bath until the monomer were completely dissolved. Then, acetic acid (3.0 mL), aniline (21.9 µl, 0.240 mmol) and TAPB solution (27.2 mg,

0.0800 mmol) were subsequently added. The mixture was kept at 90 ºC in an oil bath for 1h without any shaking or stirring. After completion of the reaction, the mixture was allowed to cool naturally to room temperature. The obtained yellow MeS/TF-COF colloid was centrifugated at 7000 rpm for 10 min, the supernatant solution was decanted, the precipitate was washed with acetonitrile, acetone and methanol, and re-dispersed into acetonitrile.

Synthesis of core-shell MeS@TF-COF: The spherical MeS-COF (5.0 mg, corresponding to 0.012 mmol of MeSTA monomer) was dispersed in 4 mL of the mixed MeCN/PhCN (2:1, v/v) solvent and heated in 90 ºC oil bath. Then, separate solution of TAPB (5.6 mg, 0.016 mmol in 2 mL of 2:1 (v/v) MeCN/PhCN solvent) and TFTA (4.9 mg, 0.024 mmol in 1.7 mL of 2:1 (v/v) MeCN/PhCN solvents with glacial acetic acid, 0.30 mL) were charged into separate syringes and placed on the dual-syringe pump. These solutions were slowly dropped into the vial with MeS-COF dispersion at a rate of 1 equiv. h^{-1}. The shell thickness was tuned by increasing the amount of TFTA monomer (2, 2.25, 2.5, 2.75 and 3 equiv.) relative to the MeSTA monomer in MeS-COF core. The obtained yellow core-shell MeS@TF-COF was centrifuged at 7000 rpm for 10 min, washed with acetonitrile, acetone and methanol, and dispersed into acetonitrile. The shell thicknesses were determined based on the elemental profile from EDS mapping to range from ~26 nm to 75 nm.

Synthesis of core-shell MeO@TF-COF: MeO@TF-COF was prepared following the procedure described above for MeS@TF-COF, using MeO-COF (4.2 mg, corresponding to 0.0011 mmol of MeOTA in the $C_{78}H_{72}N_6O_6$ repeat unit of MeO-COF) as seeds, and adding TAPB (0.014 mmol for thinner shell; 0.021 mmol for thicker shell) and TFTA (0.021 mmol for thinner shell; 0.032 mmol for thicker shell) monomers for growing the shell. The shell thickness was tuned by increasing the amount of TFTA monomer (2 and 3 equiv.)

Preparation of TA-COF with $Sc(OTf)_3$ catalyst: This synthetic procedure was followed by the Dichtel's method.[2] A vial was charged with TAPB (7.5 mg, 0.021 mmol) and PDA (4.3 mg, 0.032 mmol) in MeCN (5 mL), and the solution was sonicated briefly to dissolve the monomers. $Sc(OTf)_3$ (5.0 mg, 0.010 mmol) was subsequently added. The mixture was reacted without any shaking or stirring at room temperature for 2 days. The obtained colloid was centrifugated at 7000 rpm for 10 min, the supernatant solution was decanted, the precipitate was washed with acetonitrile, acetone and methanol, and re-dispersed into acetonitrile.

MeO-COF was synthesized following the same procedure above using TAPB and DMTA monomers.

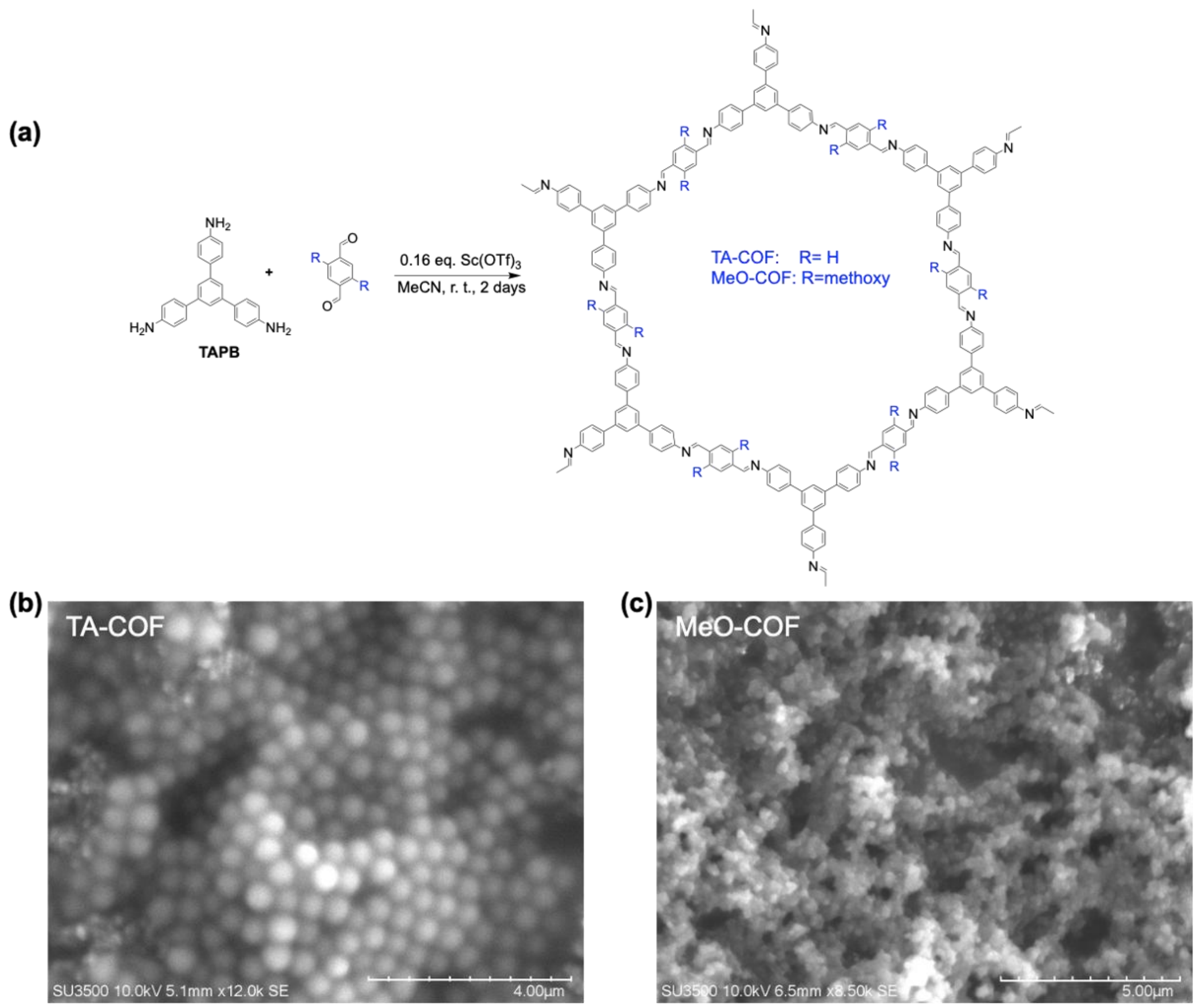

Figure S1 (a) Schematic of synthesis and structures of TA-COF and MeO-COF using $Sc(OTf)_3$ as the catalyst, and the resulting SEM images of (b) TA-COF and (c) MeO-COF.

3. Characterization of Spherical COF NPs

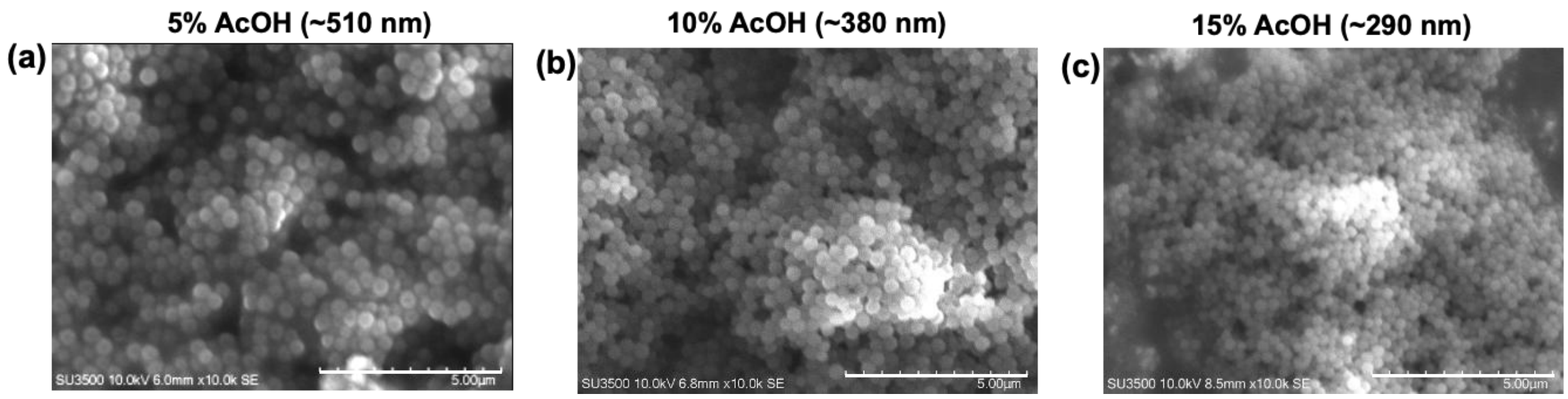

Figure S2 SEM images of spherical MeO-COF prepared in 5, 10, and 15 v/v% AcOH, corresponding to the total volume of reaction solvent.

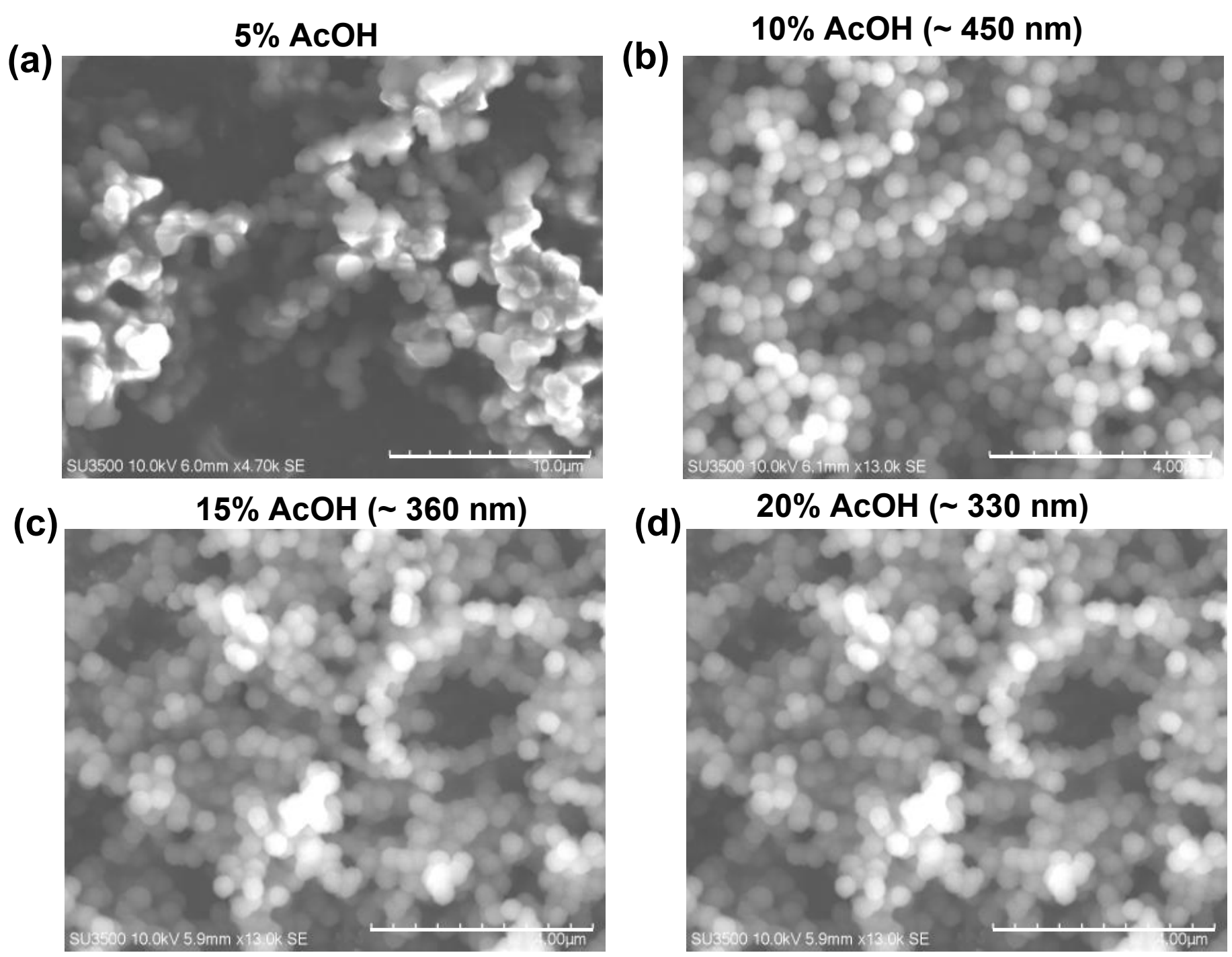

Figure S3 SEM images of spherical TF-COF prepared in 5, 10, 15 and 20 v/v% AcOH, corresponding to the total volume of reaction solvent.

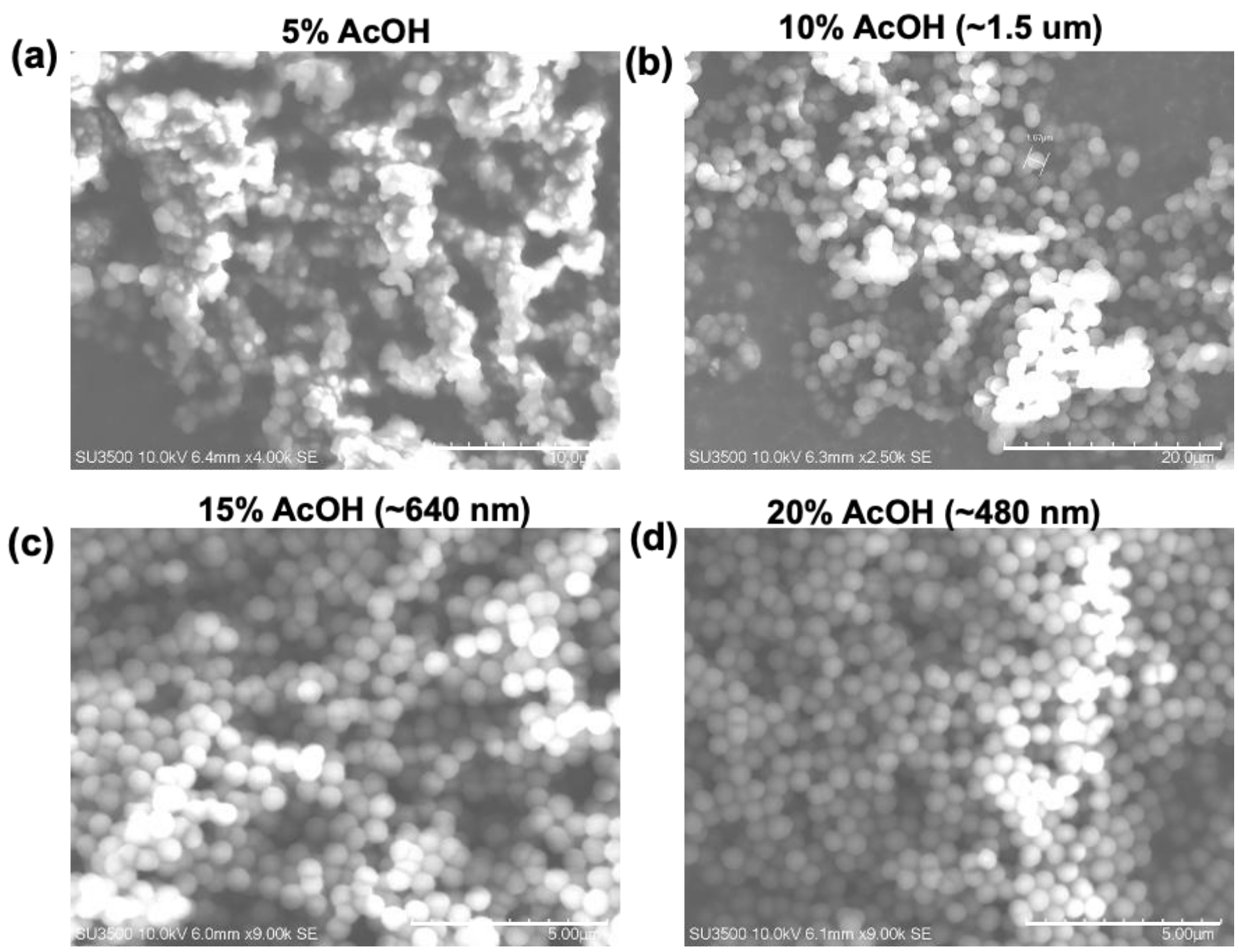

Figure S4 SEM images of spherical MeS-COF prepared in 5, 10, 15 and 20 v/v% AcOH, corresponding to the total volume of reaction solvent.

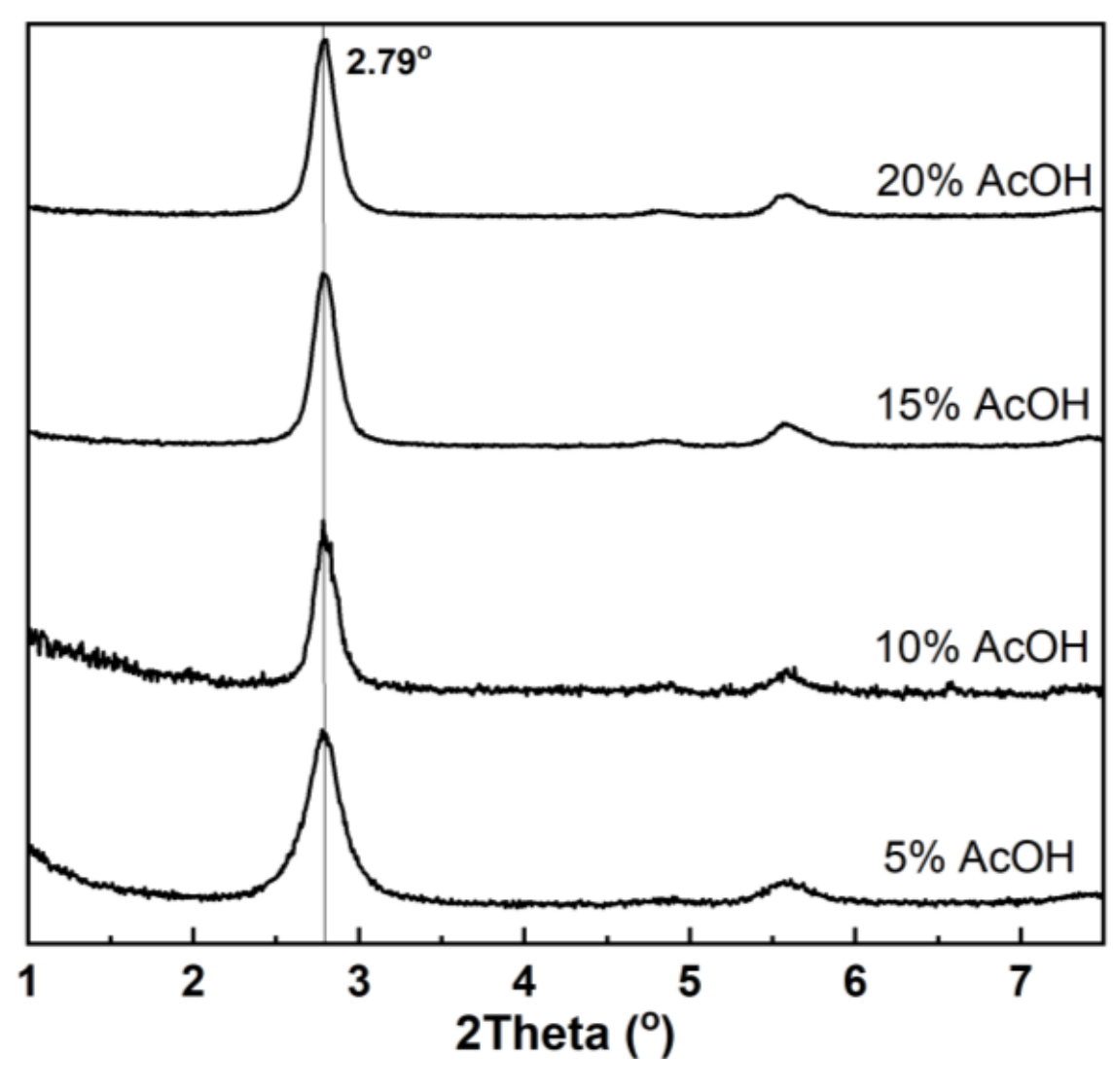

Figure S5 SAXS spectra of MeS-COF prepared with different amount of catalyst.

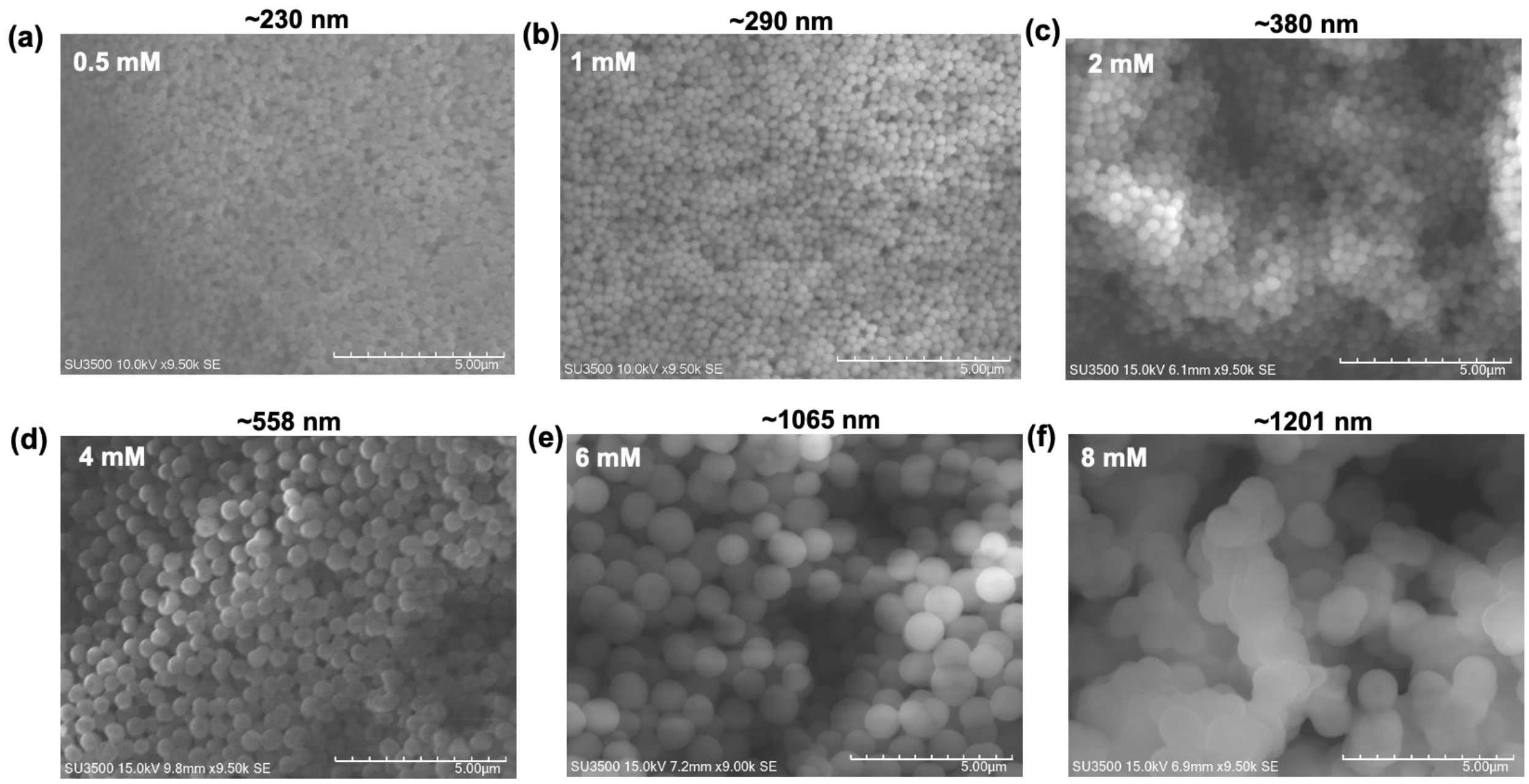

Figure S6 SEM images of MeS-COF prepared at different TAPB monomer concentrations (0.5 mM, 1 mM, 2 mM, 4 mM, 6 mM, and 8 mM).

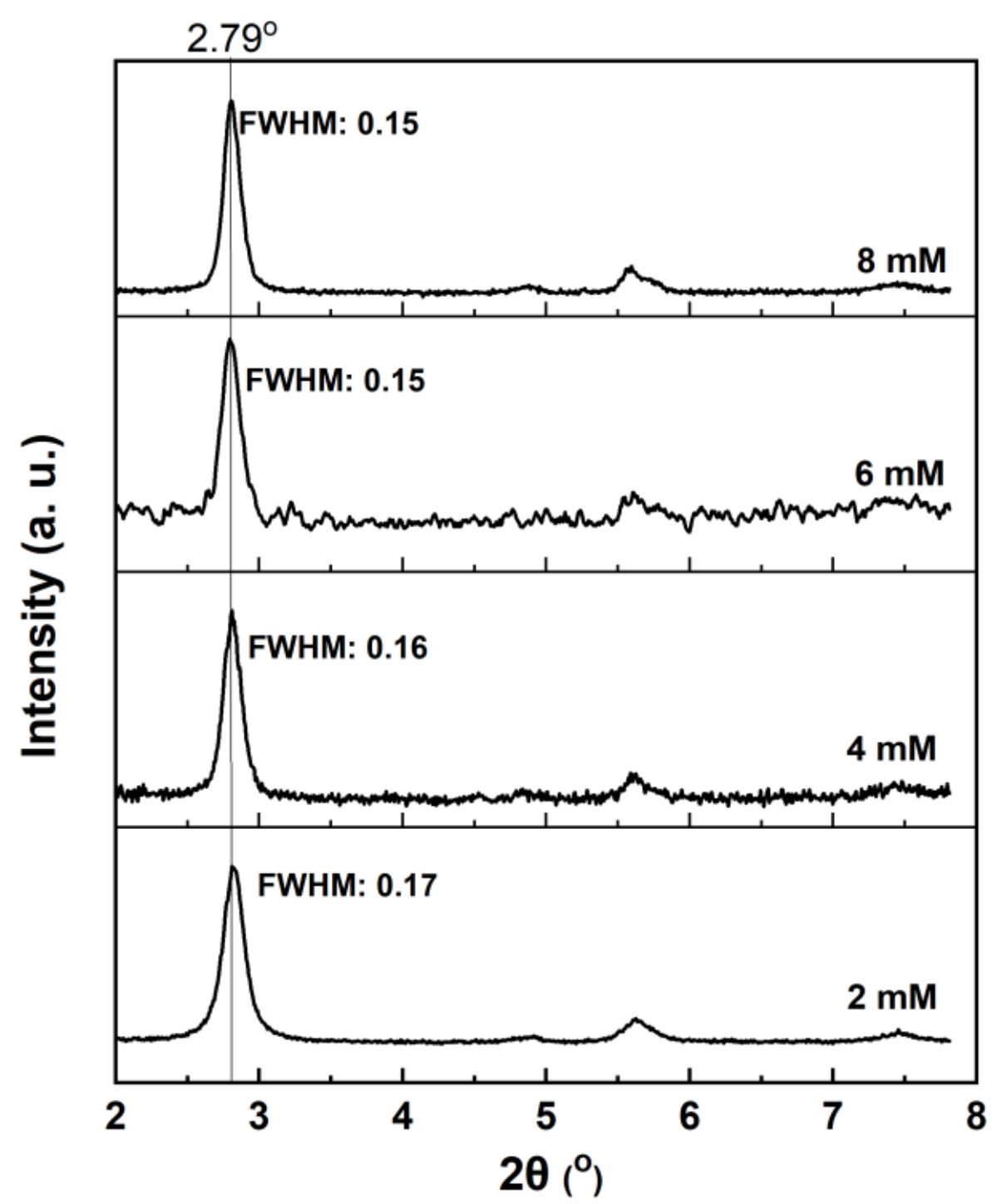

Figure S7 SAXS spectra of MeS-COF prepared at different TAPB monomer concentrations.

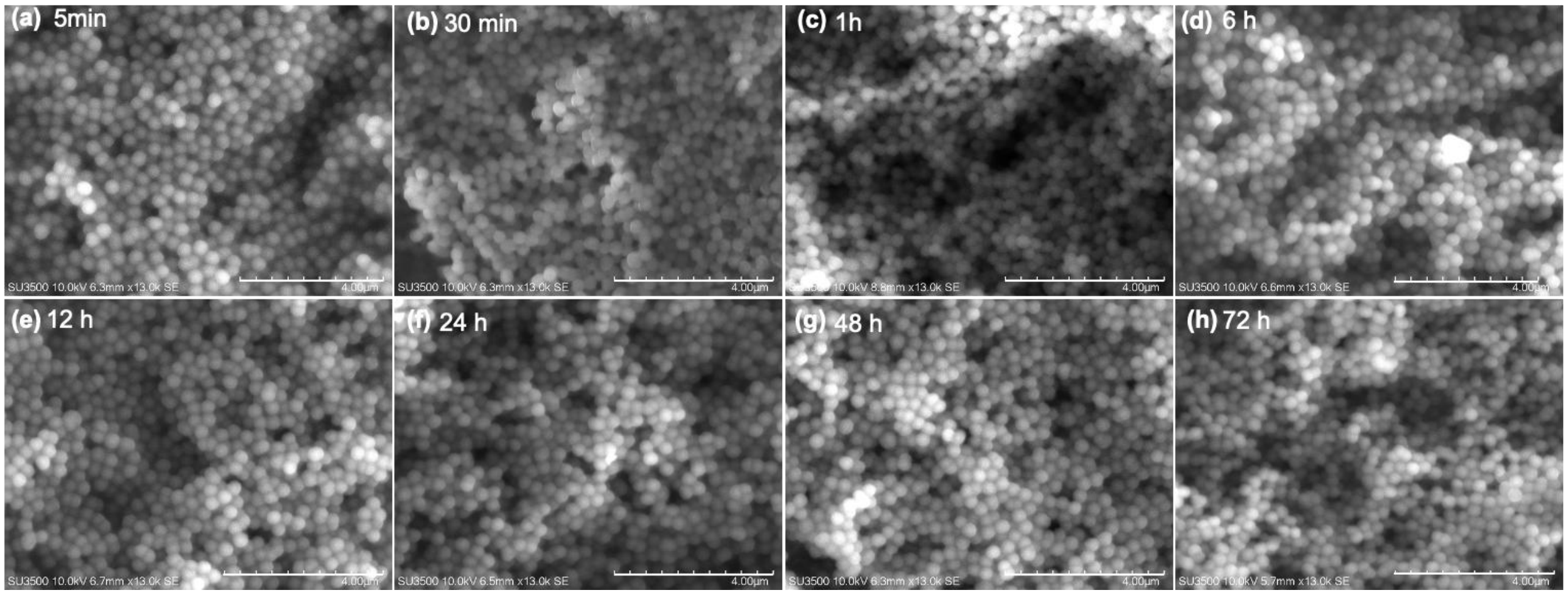

Figure S8 SEM images of MeS-COF after different reaction times.

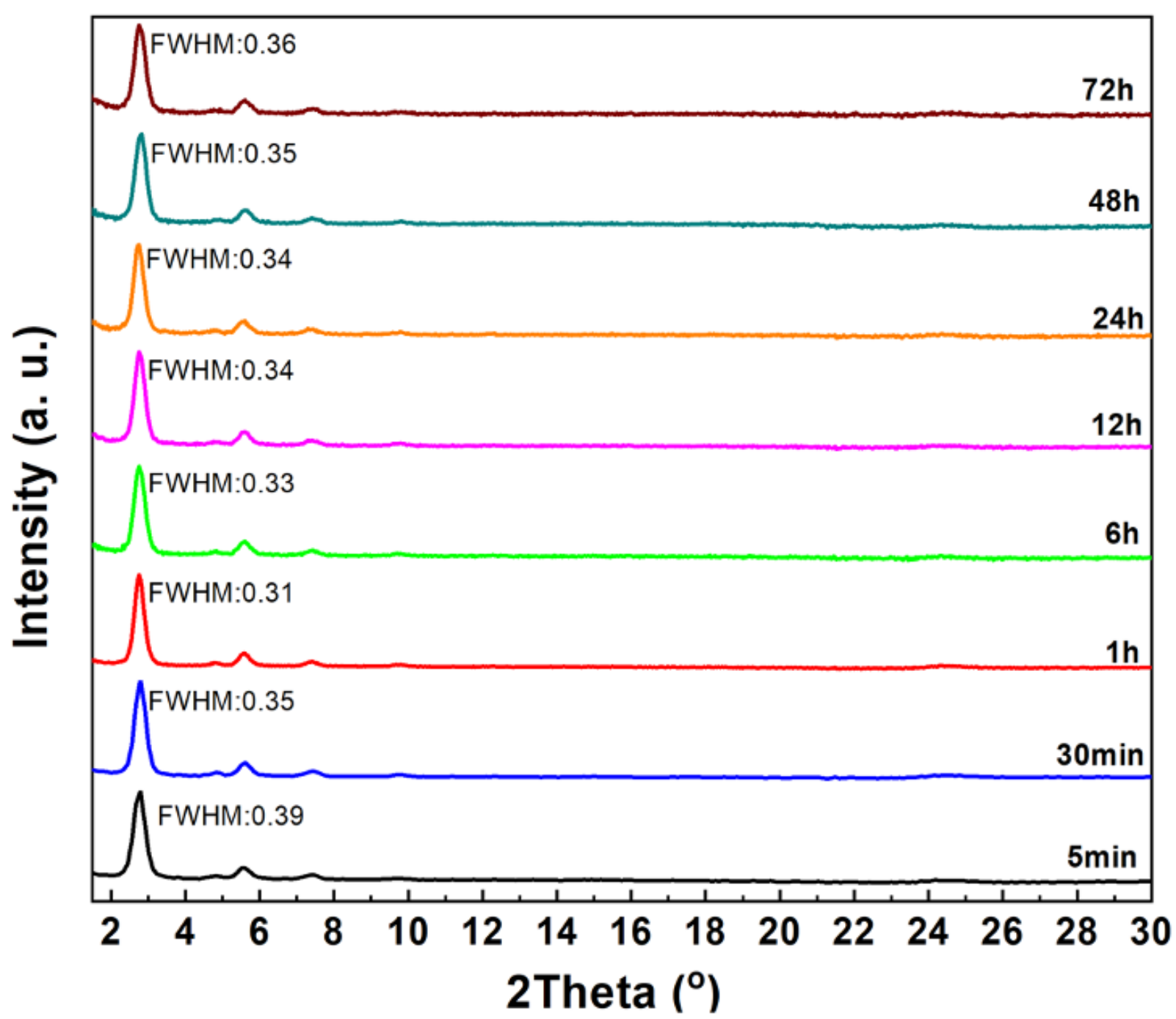

Figure S9 WAXS spectra of MeS-COF after different reaction time using a TAPB concentration of 4 mM and 20 vol% AcOH.

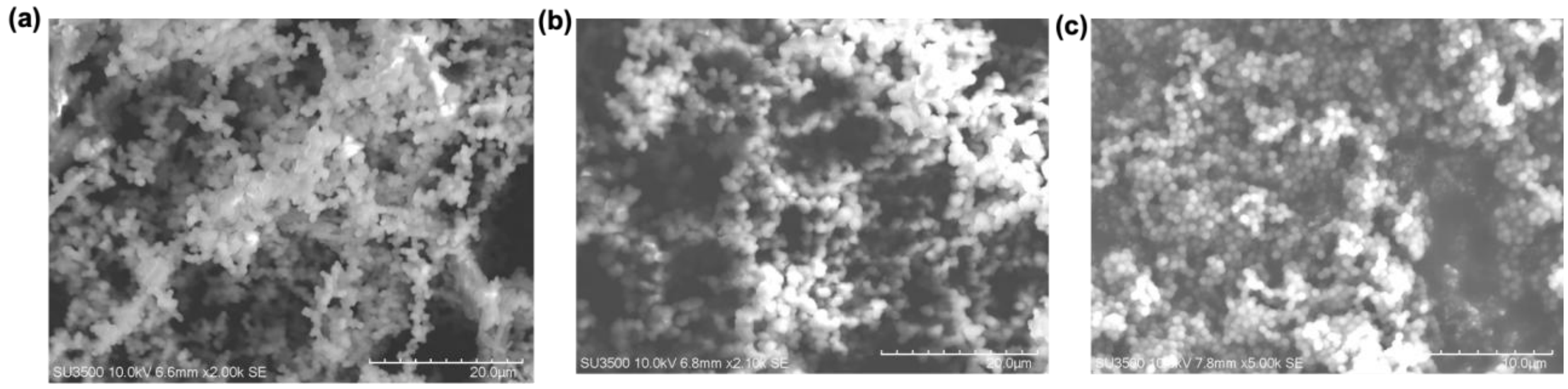

Figure S10 SEM images of spherical DCl-COF synthesized using (a) $Sc(OTf)_3$ catalyst, (b) TFA catalyst, and (c) AcOH catalyst.

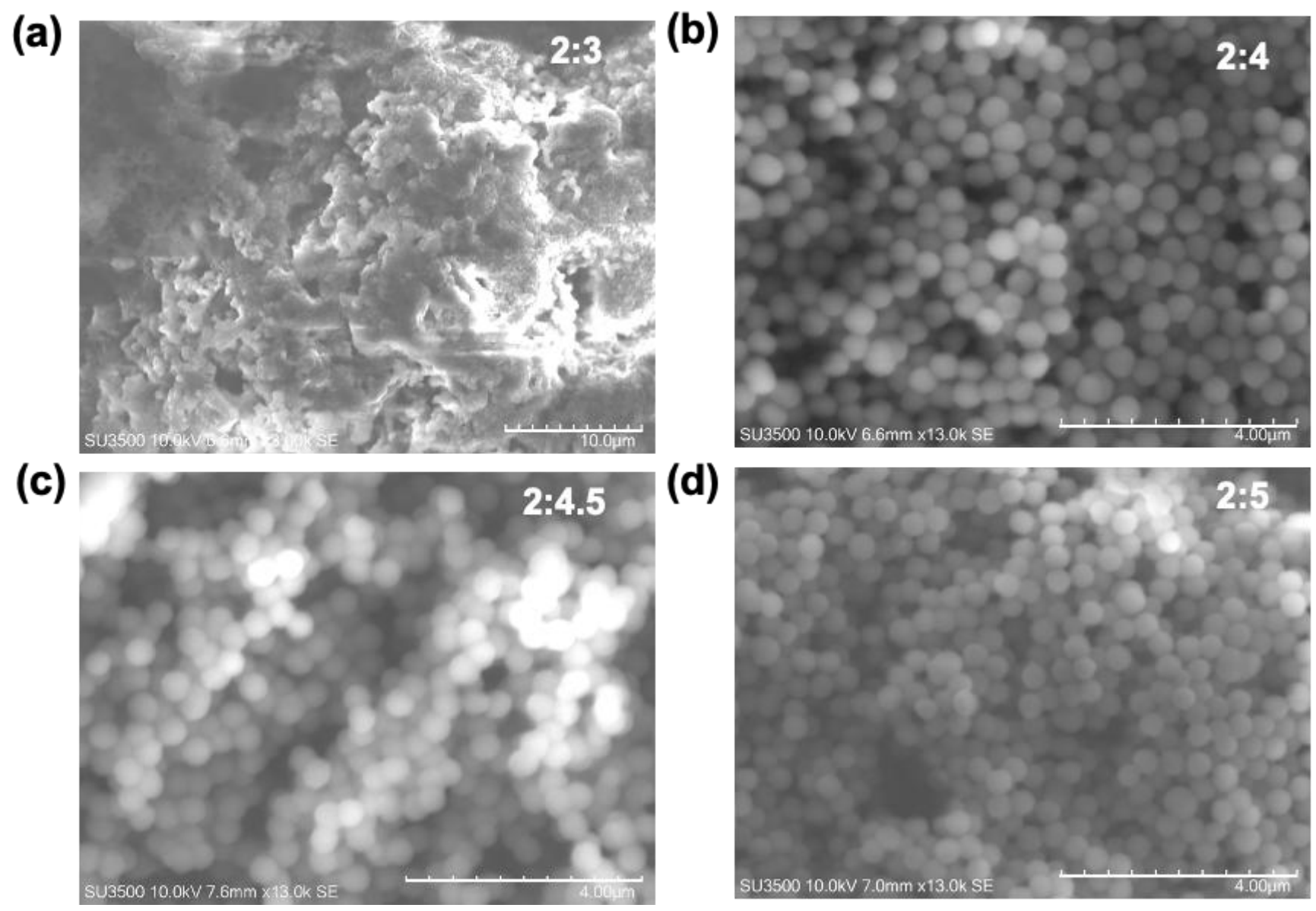

Figure S11 SEM images of DCl-COF prepared with the different Tz-TBA:DCBDA ratios.

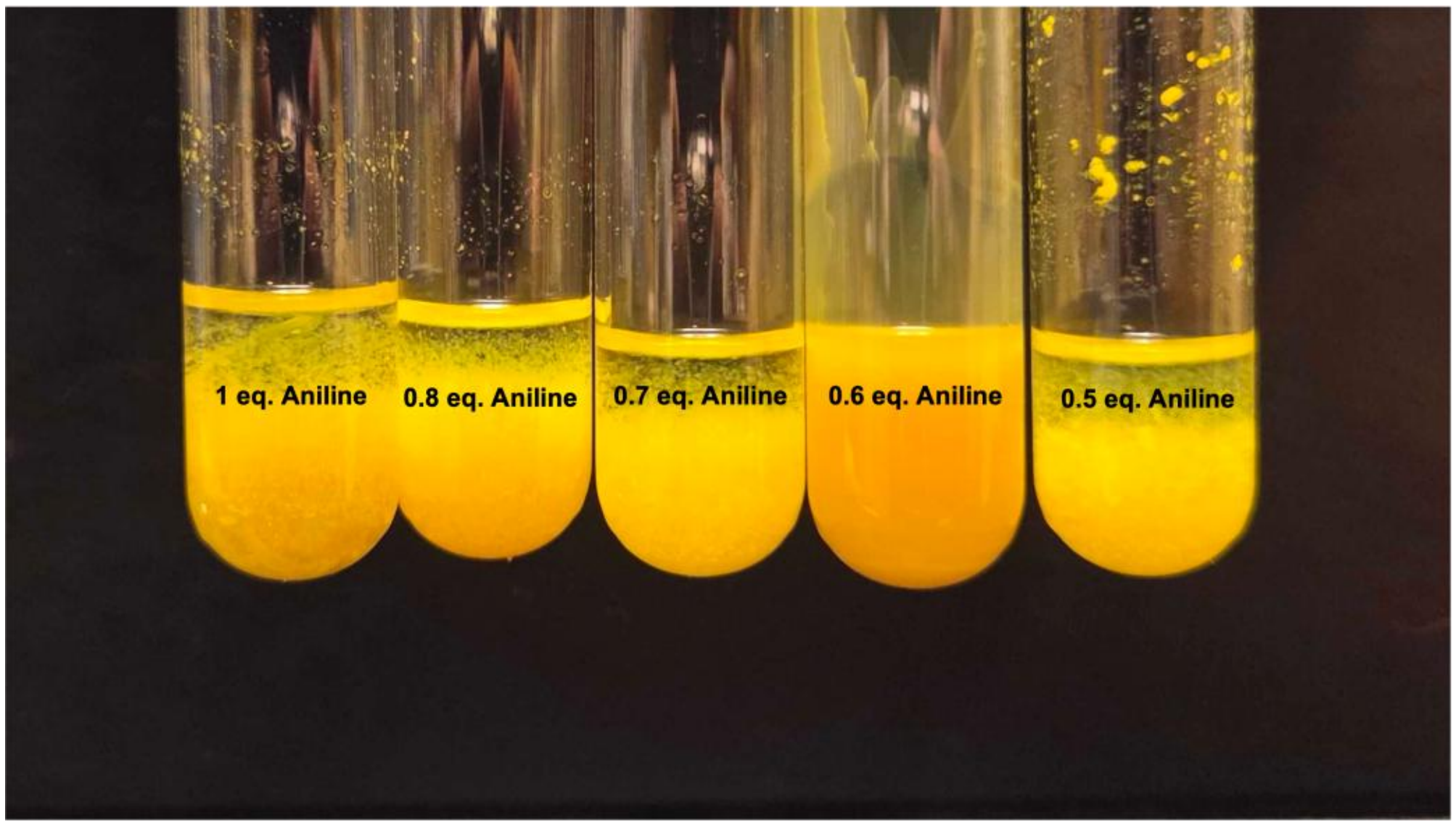

Figure S12 Photographs of DCl-COF suspension in acetonitrile reacting with varying aniline equivalents (1.0 to 0.5 equivalent per imine bond of DCBDA linker), which is prepared with the Tz-TBA:DCBDA ratios of 2:4.5.

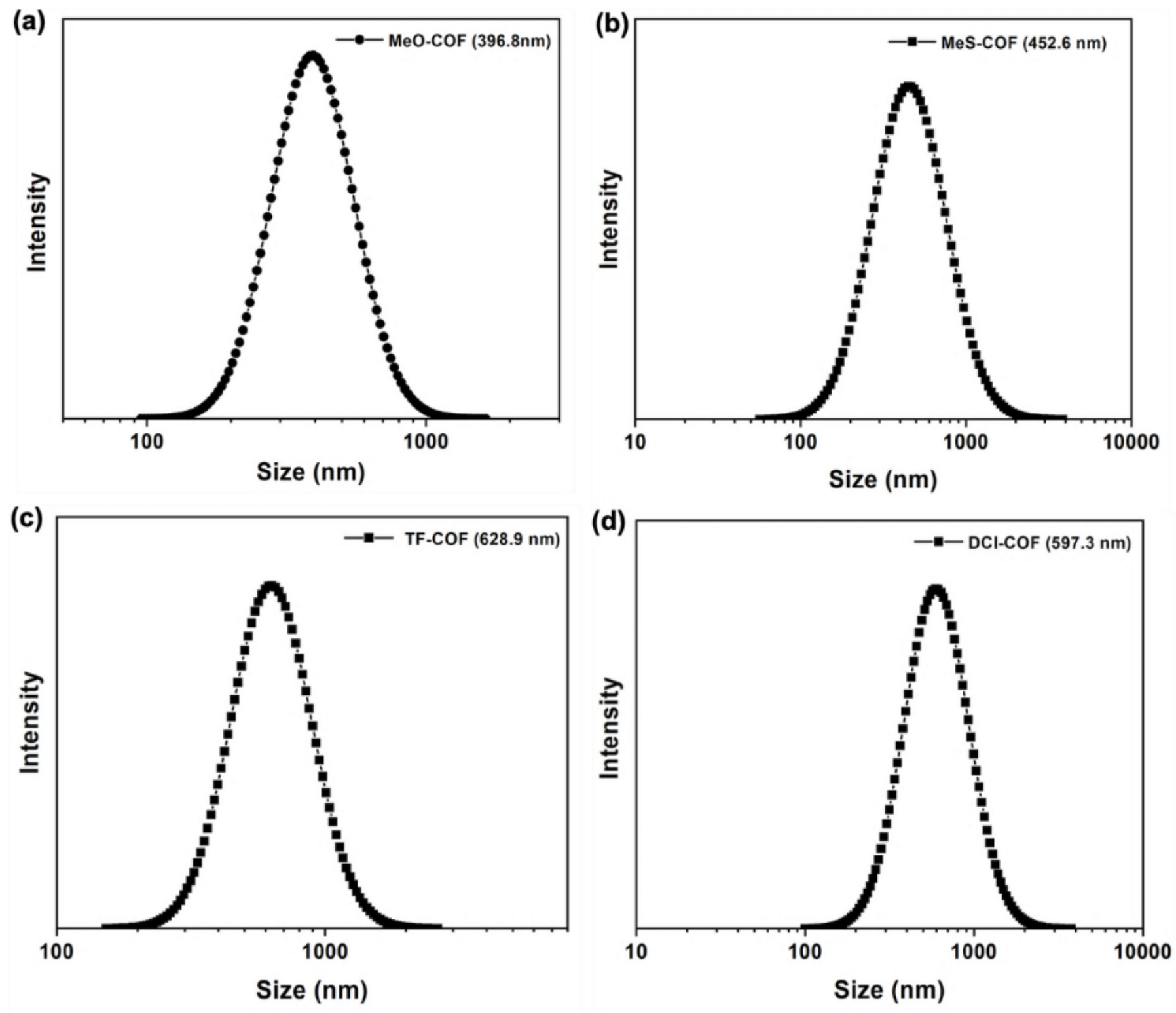

Figure S13 DLS profiles of MeO-COF, MeS-COF, TF-COF and DCl-COF.

The MeO-COF and MeS-COF particle sizes measured by DLS are in good agreement with those obtained from TEM (~386 nm and 434 nm, respectively, Figure 1). However, the size of TF-COF and DCl-COF from DLS showed ~200 nm larger than those from TEM (~420 nm and 435 nm, respectively), possibly due to partial aggregation of these particles.

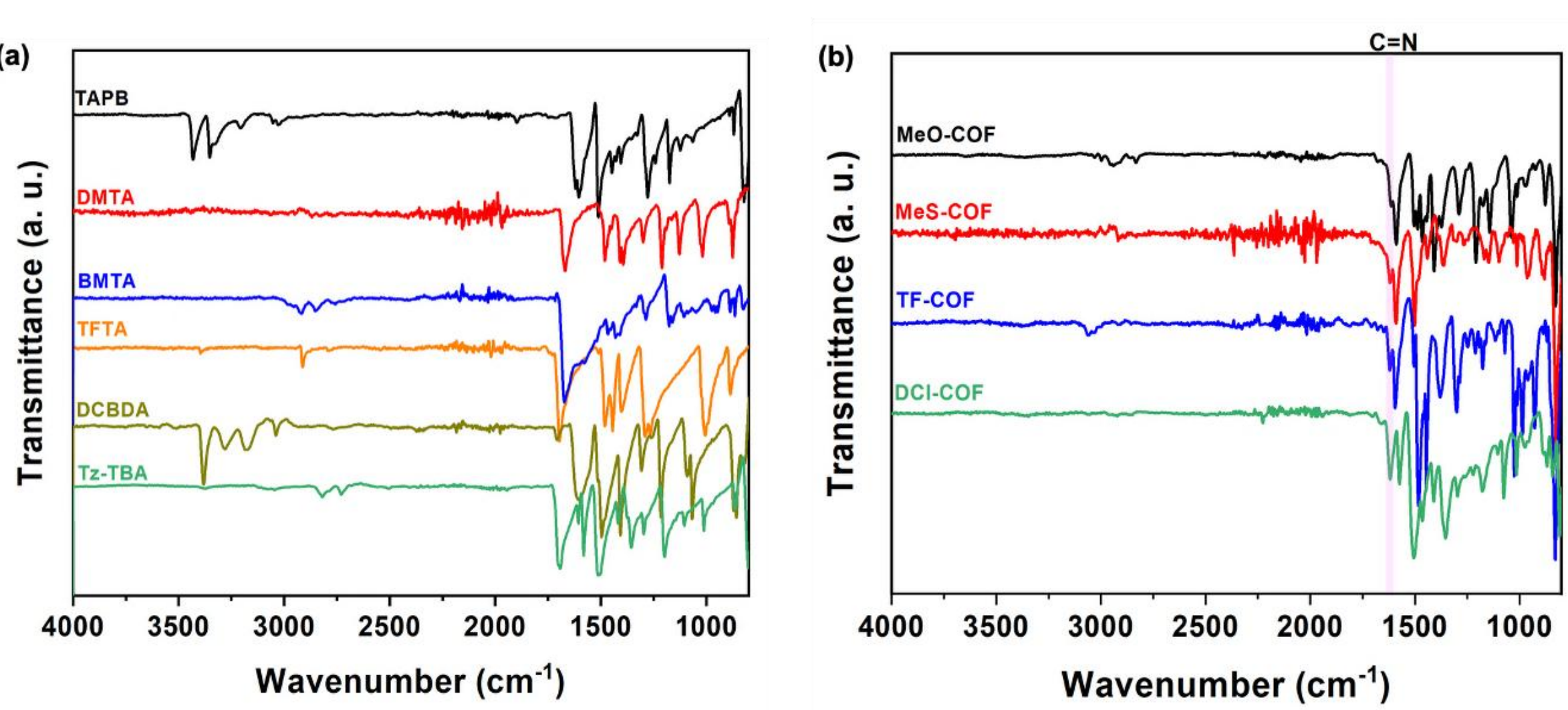

Figure S14 FT-IR spectra of TAPB, DMTA, BMTA, TFTA, DCBDA and Tz-TBA monomer (a) and their resulting polymers: MeO-COF, MeS-COF, TF-COF and DCl-COF (b).

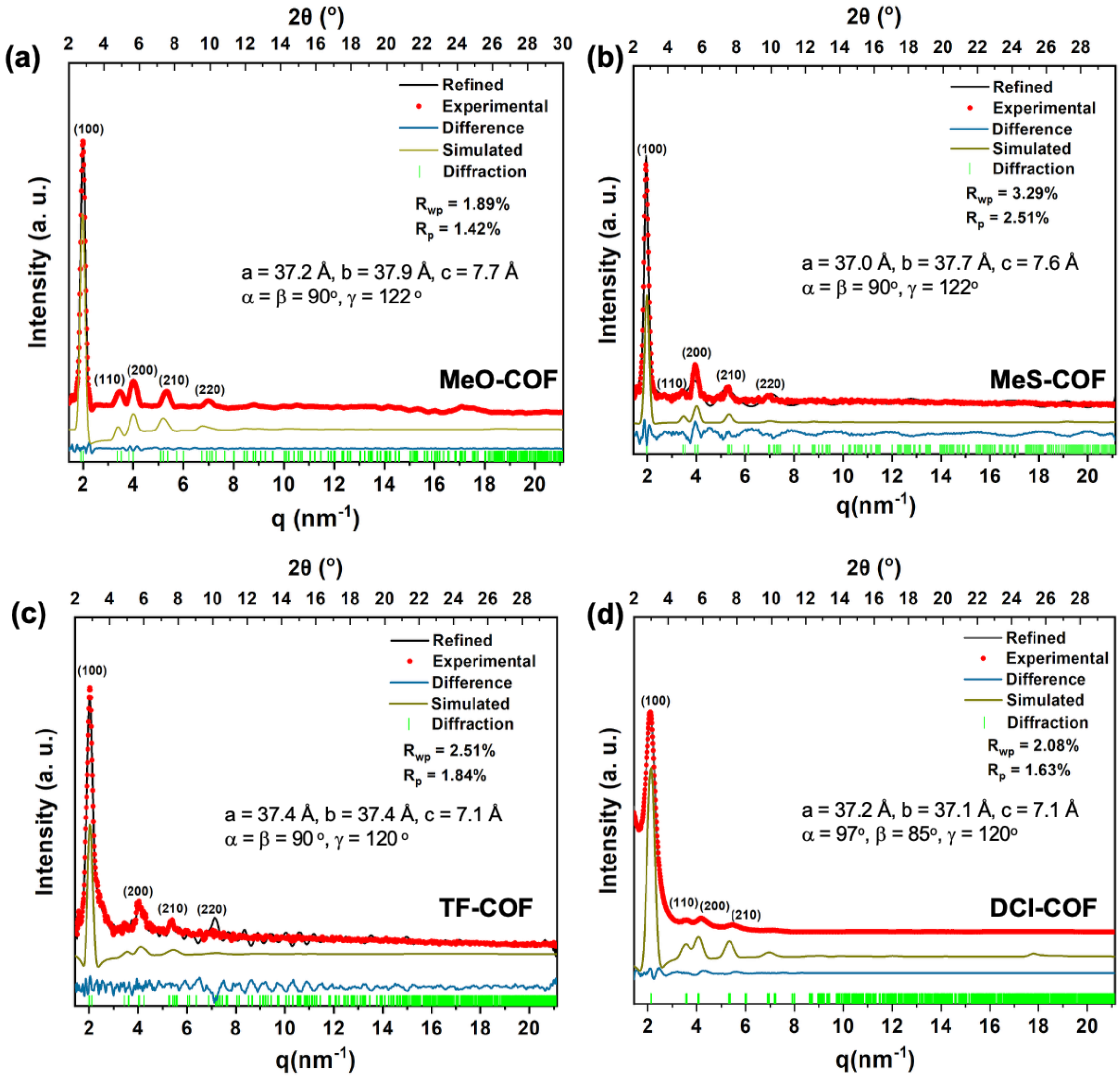

Figure S15 WAXS pattern with full-profile Pawley refinement of MeO-COF, MeS-COF, TF-COF and DCl-COF, where simulated pattern is from DFT-optimized unit cell of AA-stacked COFs.

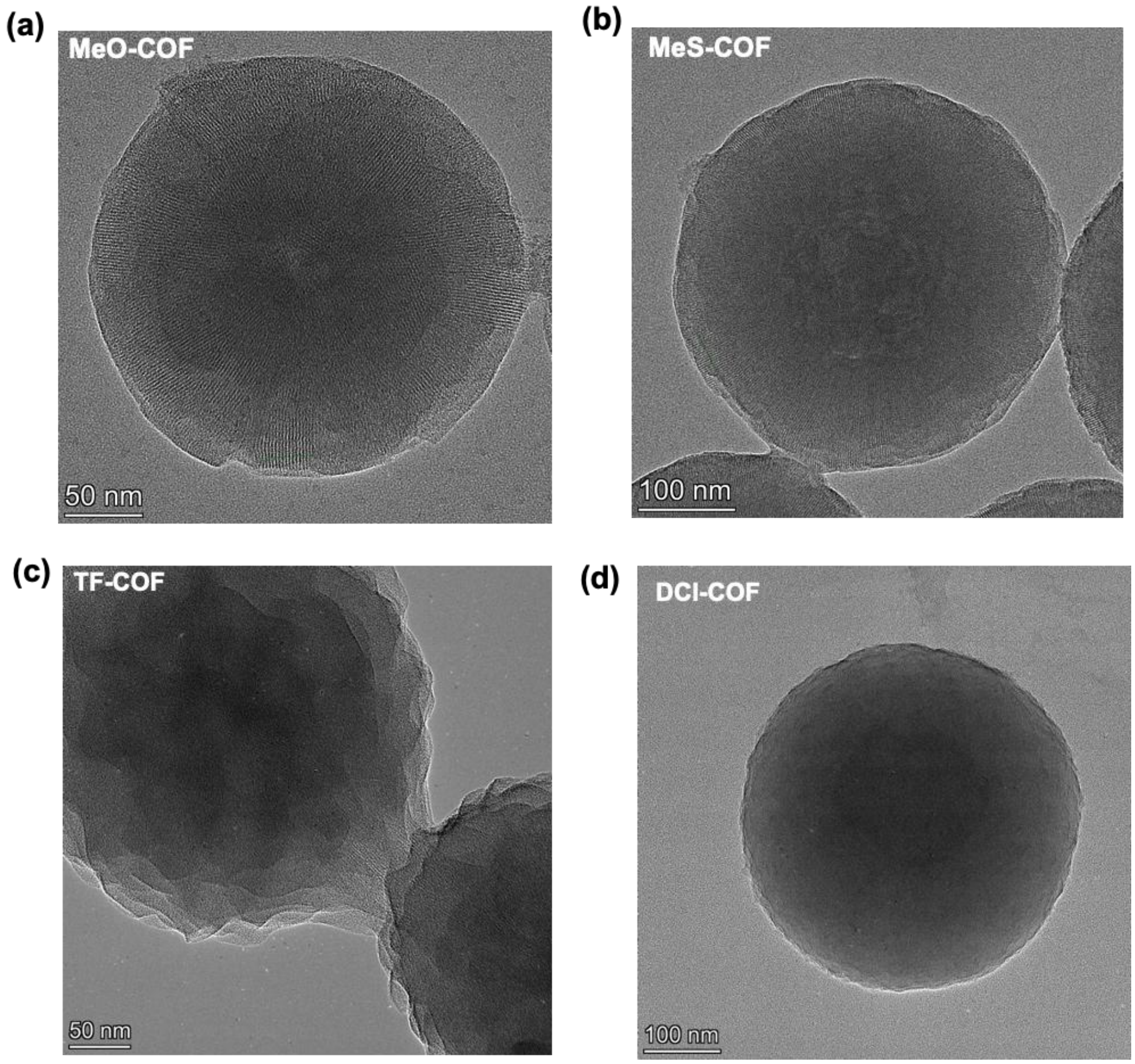

Figure S16 HR-TEM image of the (a) MeO-COF, (b) MeS-COF, (c) TF-COF and (d) DCl-COF.

4. DFT Calculations of NMR Chemical Shifts

NMR shielding tensors were calculated using the gauge-including atomic orbital (GIAO) method at the B3LYP/6-31G(d) level. Isotropic magnetic shielding constants (σ) were converted to chemical shifts (δ) using tetramethylsilane (TMS) as the reference according to:

$$\delta = \sigma_{\mathrm{TMS}} - \sigma_{\mathrm{calc}}$$

where $\sigma_{\mathrm{TMS}}$is the calculated isotropic shielding constant of the reference compound and $\sigma_{\mathrm{calc}}$is the shielding constant of the nucleus in the molecule of interest. A reference shielding value of 189.64 ppm was calculated for TMS.

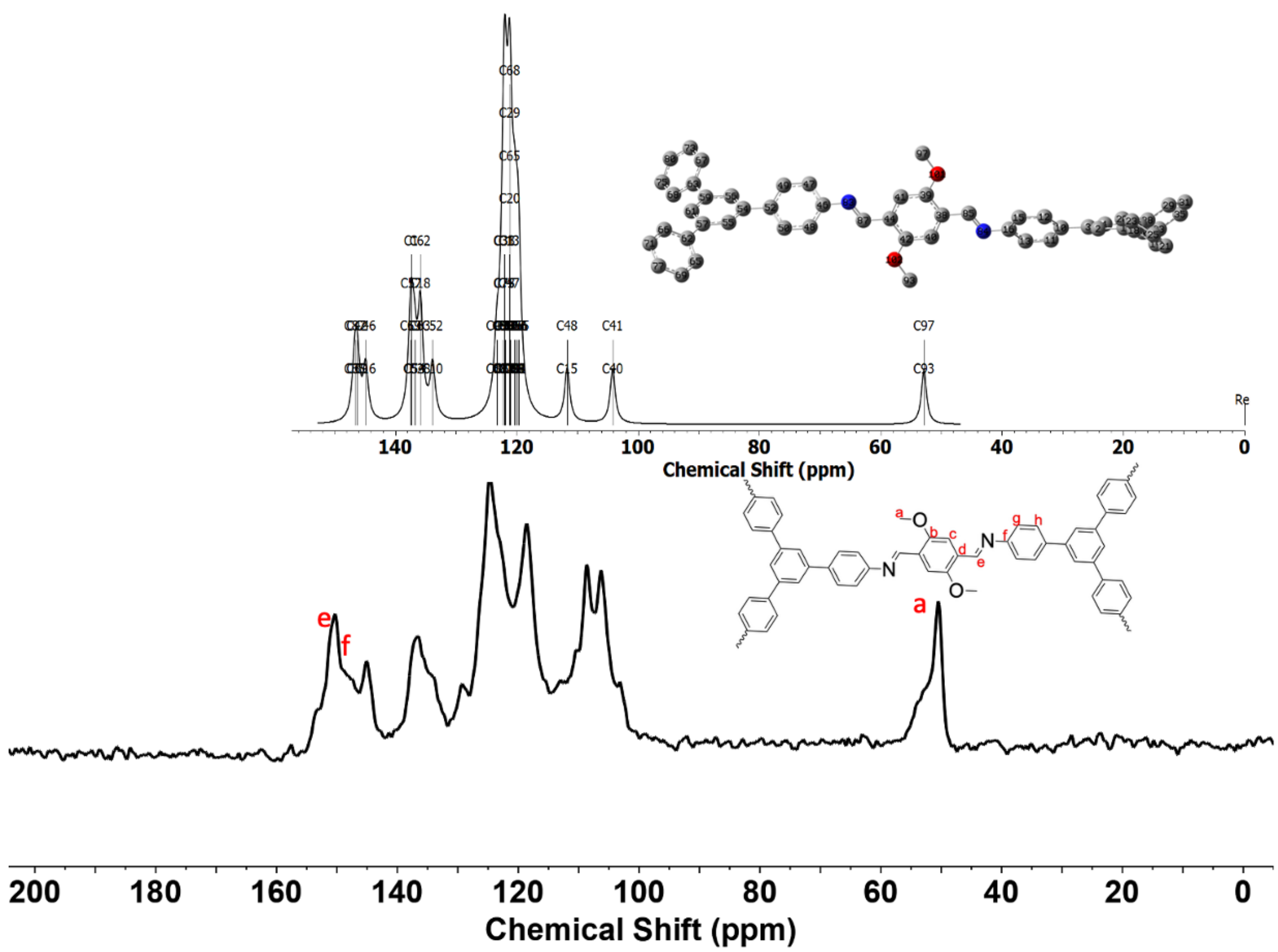

Figure S17 Solid state ^{13}C-NMR spectrum and DFT calculated spectrum of MeO-COF.

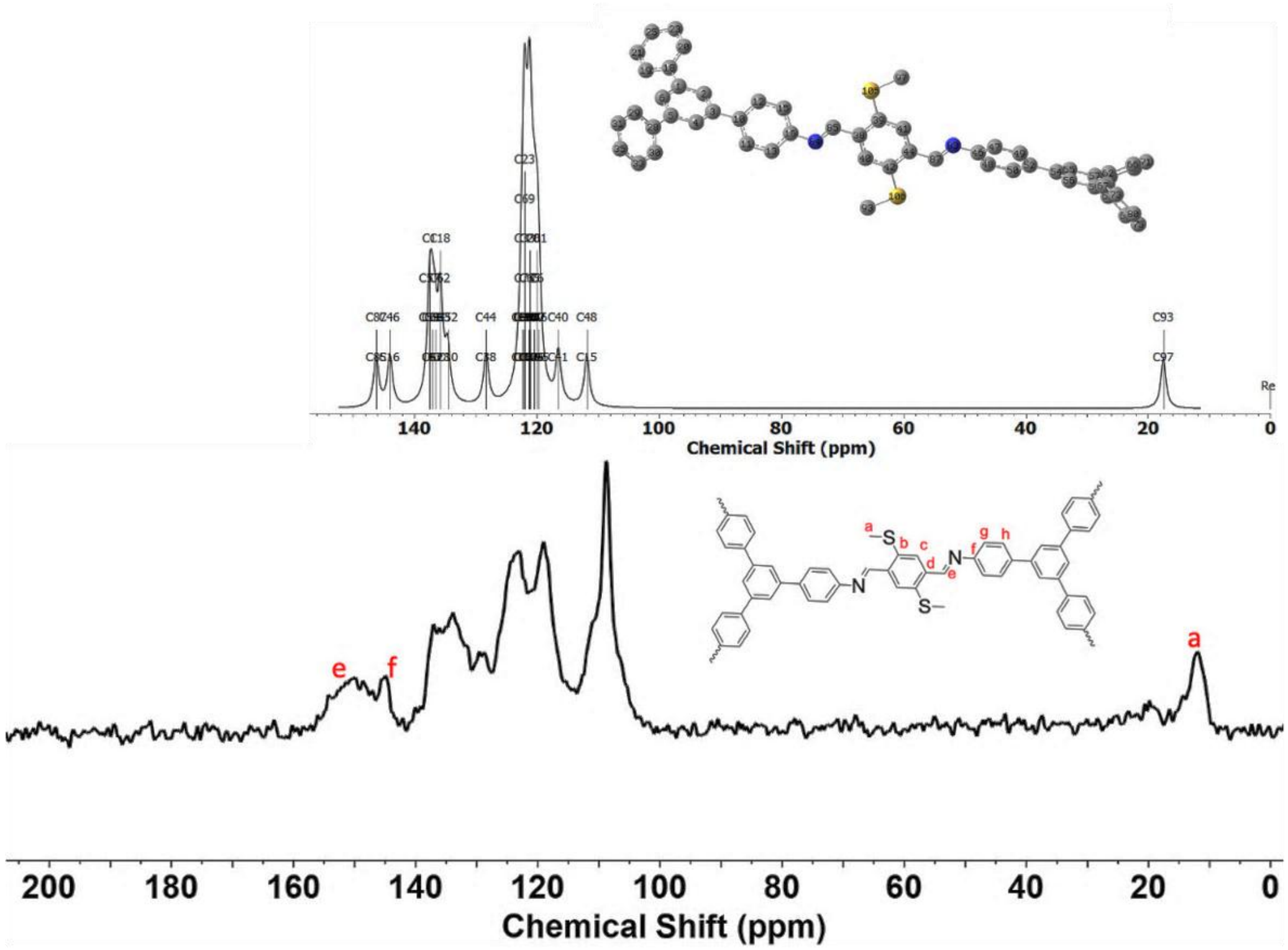

Figure S18 Solid state ^{13}C-NMR spectrum and DFT calculated spectrum of MeS-COF.

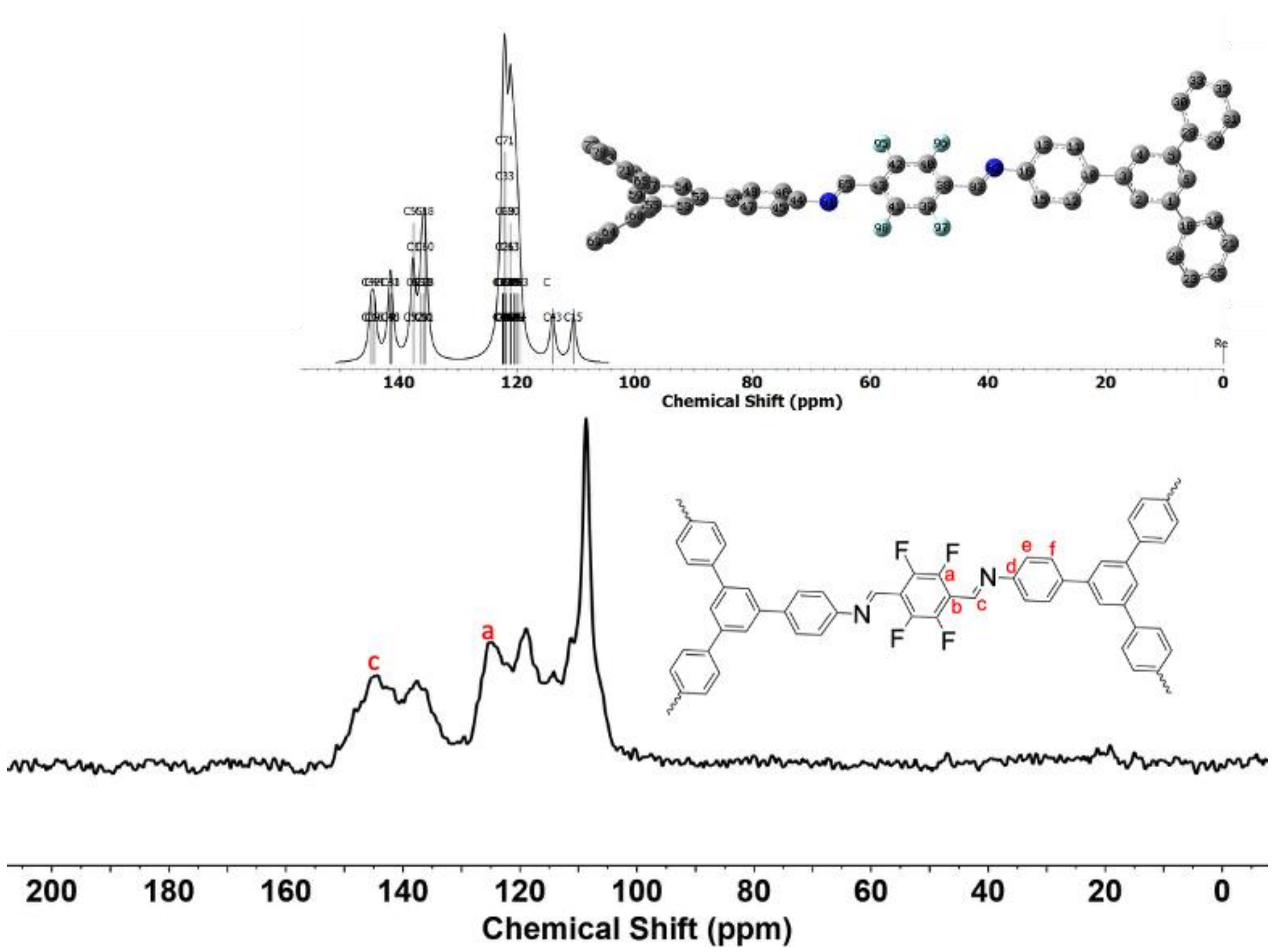

Figure S19 Solid state ^{13}C-NMR spectrum and DFT calculated spectrum of TF-COF.

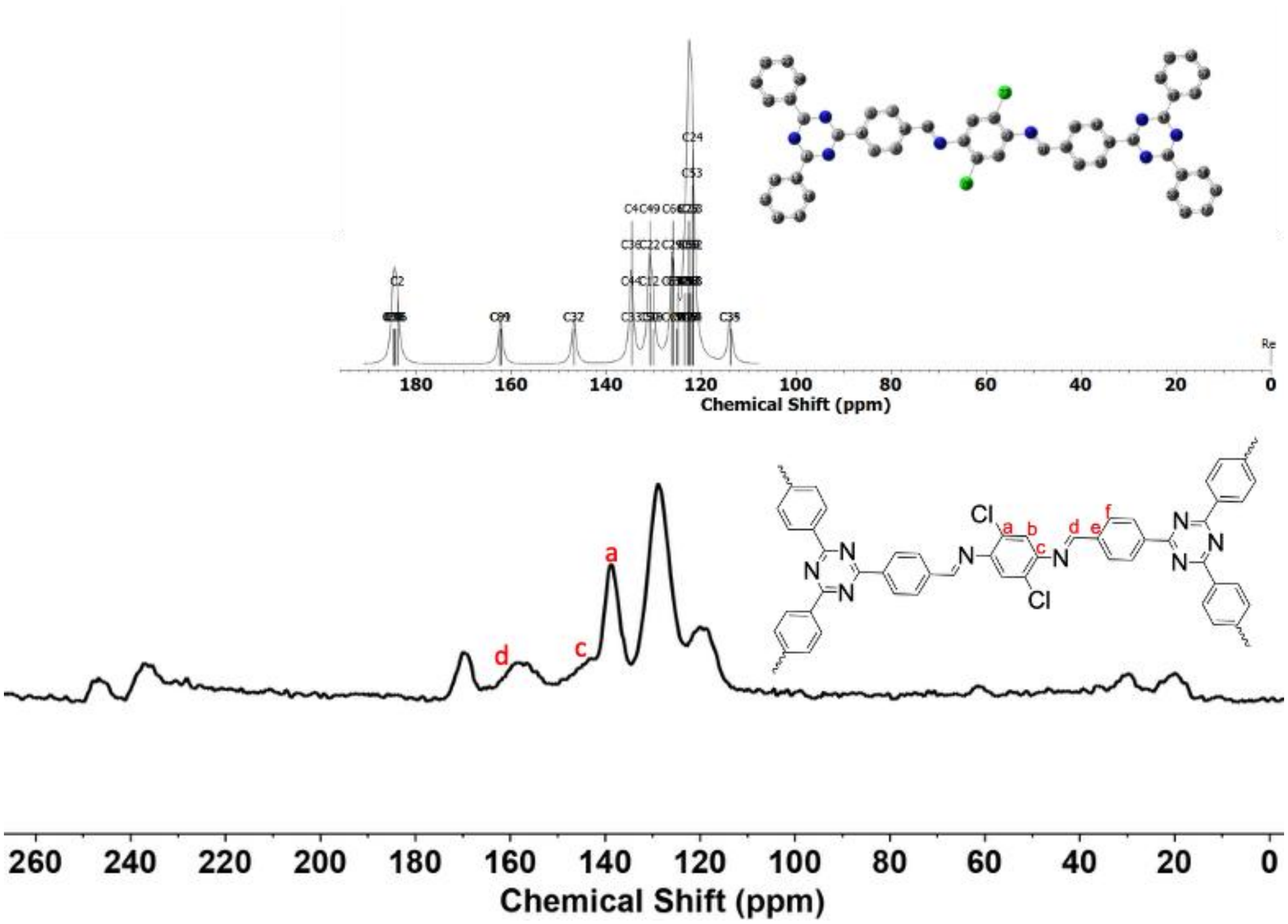

Figure S20 Solid state ^{13}C-NMR spectrum and DFT calculated spectrum of DCl-COF.

Table S1. The calculated C-NMR parameters of DFT-optimized COF structures.

MeO-COF		MeS-COF		TF-COF		DCI-COF	
Atoms	Chemical shift (ppm)	Atoms	Chemical shift (ppm)	Atoms	Chemical shift (ppm)	Atoms	Chemical shift (ppm)
C85	146.6768	C85	146.2516	C39	144.7828	C1	184.878
C87	146.6767	C87	146.2514	C42	144.7828	C47	184.7919
C39	146.2367	C16	144.0066	C16	144.2599	C48	184.5291
C42	146.2367	C46	144.0065	C44	144.2594	C3	184.4005
C16	144.8764	C5	137.4971	C40	141.5573	C46	183.9808
C46	144.8761	C59	137.4965	C41	141.5572	C2	183.9487
C5	137.4093	C57	137.4764	C85	141.4157	C89	162.2639
C59	137.4051	C1	137.4753	C83	141.4156	C91	162.1348
C57	137.3883	C42	137.0605	C57	137.6689	C37	146.7926
C1	137.3877	C39	137.0602	C5	137.668	C32	146.6899
C54	136.844	C3	136.6329	C1	137.6352	C33	134.6806
C3	136.8435	C54	136.6328	C55	137.6352	C44	134.6391
C28	135.8603	C28	135.7647	C3	136.2543	C36	134.6375
C63	135.8563	C63	135.7639	C52	136.2533	C4	134.6115
C18	135.8431	C62	135.7544	C50	135.9156	C50	130.835
C62	135.8431	C18	135.7537	C10	135.9151	C12	130.8181
C10	133.836	C10	134.5683	C61	135.6409	C22	130.8036
C52	133.836	C52	134.567	C28	135.6395	C49	130.8028
C38	123.241	C38	128.2825	C60	135.629	C38	130.2012
C44	123.2409	C44	128.2816	C18	135.6284	C10	130.1179
C12	122.2119	C12	122.3756	C45	122.4887	C19	126.0557
C50	122.2115	C50	122.375	C13	122.4879	C63	126.0433
C11	122.0146	C11	122.1106	C47	122.3746	C29	125.9943
C49	122.0145	C49	122.11	C11	122.3743	C66	125.9834
C75	122.0088	C31	122.0488	C48	122.2948	C7	125.072
C31	122.0082	C75	122.0488	C12	122.2947	C39	125.0292
C21	121.9467	C21	121.9691	C31	122.0815	C42	123.5394
C71	121.9467	C71	121.9691	C73	122.0814	C6	123.5297
C73	121.9416	C73	121.9622	C21	122.0422	C40	122.8245
C33	121.9408	C33	121.9621	C69	122.0421	C9	122.8118
C69	121.8719	C69	121.9139	C33	122.0188	C17	122.6611
C23	121.8718	C23	121.9138	C71	122.0185	C55	122.6457
C19	121.2078	C13	121.2808	C23	121.9472	C61	122.6199
C66	121.2077	C47	121.2805	C67	121.9471	C25	122.608
C47	121.1632	C66	121.2114	C64	121.2184	C15	122.52
C13	121.1631	C19	121.2112	C19	121.2182	C57	122.5177
C20	121.1257	C29	121.1317	C66	121.1557	C59	122.4816
C65	121.1256	C68	121.1312	C29	121.1549	C27	122.4574
C29	121.1239	C65	121.1234	C63	121.1063	C5	122.281
C68	121.1219	C20	121.123	C20	121.1062	C41	122.2523
C30	121.0609	C30	121.0462	C65	121.0416	C14	121.9202
C67	121.0591	C67	121.0459	C30	121.0409	C51	121.9185
C80	120.406	C35	120.4733	C35	120.5817	C54	121.8338
C35	120.4057	C80	120.4731	C78	120.5815	C13	121.8306
C25	120.3092	C25	120.3733	C75	120.5028	C52	121.801
C77	120.3092	C77	120.3733	C25	120.5026	C23	121.7975
C4	119.8989	C4	119.9148	C59	120.2512	C53	121.7607
C56	119.8968	C56	119.9139	C6	120.2505	C24	121.7133
C6	119.6852	C6	119.8891	C54	119.9545	C35	113.9646
C61	119.6832	C61	119.8891	C4	119.9539	C34	113.7711
C2	119.6293	C55	119.6499	C2	119.6359		
C55	119.6293	C2	119.6492	C53	119.6352		
C15	111.654	C41	116.4495	C43	113.9583		
C48	111.654	C40	116.4483	C38	113.9579		
C40	104.15	C15	111.8116	C15	110.3972		
C41	104.15	C48	111.8106	C46	110.3972		
C93	52.8358	C97	17.5045				
C97	52.8358	C93	17.503				

5. XPS/UPS Data

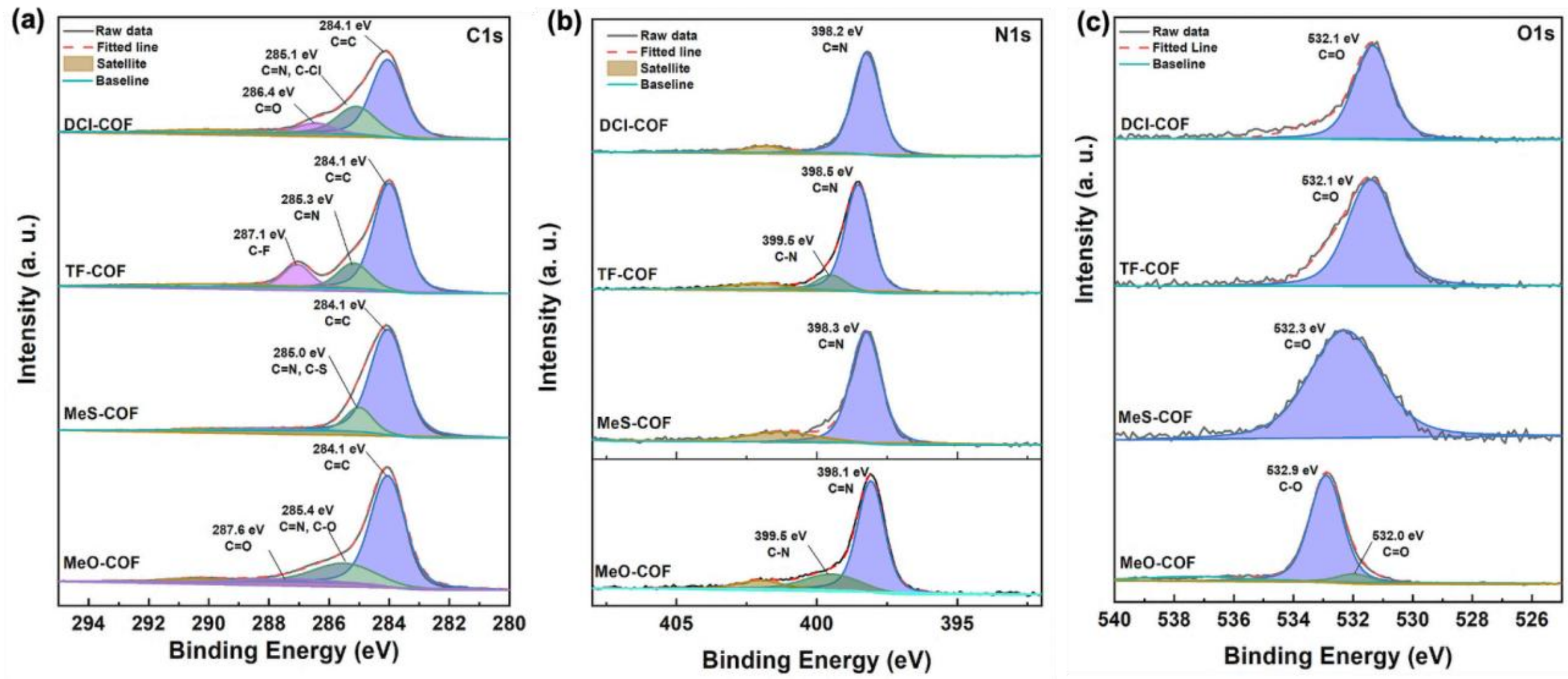

Figure S21 High-resolution XPS (a) C 1s, (b) N 1s, and (c) O 1s spectra of MeO-COF, MeS-COF, TF-COF and DCl-COF.

Table S2. XPS parameters of MeO-COF

	**Expected**		**XPS Survey***				**Binding Energy (eV)**	**FWHM (eV)**	**Atomic Ratio**	
	Atomic %	Atomic ratio	Atomic %	Atomic ratio					Expected	XPS Deconvolution
**C**	86.6	13	85.5±0.5	17.1±2.8	**C**	$_{sp2}$**C=C**	284.1	1.4	3.3	3.0
**N**	6.7	1	5.0±0.8	1		$_{sp3/2}$**C–O** $_{sp2}$**C=N**	285.4	2.5	1	1.0
**O**	6.7	1	9.5±1.2	1.9±0.5		$_{sp2}$**C=O**	287.6	3.4	0	0.2
					**N**	$_{sp2}$**C–N**	399.7	2.2	0	0.3
						$_{sp2}$**C=N**	398.1	1.2	1	1.0
					**O**	$_{sp2}$**C=O**	532.0	1.4	0	0.1
						$_{sp2}$**C–O**	532.9	1.3	1	1.0

*Averaged over 4 spots, standard deviation as uncertainty

Table S3. XPS parameters of MeS-COF

	Expected		XPS Survey*				Binding Energy (eV)	FWHM (eV)	Atomic Ratio	
	Atomic %	Atomic ratio	Atomic %	Atomic ratio					Expected	XPS Deconvolution
**C**	86.6	13	86.0±0.1	14.1±0.4	**C**	$_{sp2}$**C=C**	284.1	1.4	5.5	5.6
**N**	6.7	1	6.1±0.2	1		$_{sp3}$**C–S** $_{sp2}$**C=N**	285.0	1.1	1	1.0
**O**	0	0	1.7±0.2	0.3±0.04		$_{sp2}$**C=O**	288.0	3.0	0	0.2
**S**	6.7	1	6.1±0.1	1.0±0.03	**N**	$_{sp2}$**C=N**	398.3	1.3	1	1
					**O**	$_{sp2}$**C=O**	532.3	2.8	0	1

*Averaged over 4 spots, standard deviation as uncertainty

Table S4. XPS parameters of TF-COF

	Expected		XPS Survey				Binding Energy (eV)	FWHM (eV)	Atomic Ratio	
	Atomic %	Atomic ratio	Atomic %	Atomic ratio					Expected	XPS Deconvolution
**C**	80.0	12	78.8±0.5	13.6±0.8	**C**	$_{sp2}$**C=C**	284.1	1.2	4	4.0
**F**	13.3	2	13.5±0.5	2.3±0.2		$_{sp2}$**C=N**	285.3	1.3	1	1.0
**N**	6.7	1	5.8±0.3	1		$_{sp2}$**C–F**	287.1	1.2	1	1.0
**O**	0	0	1.9±0.4	0.3±0.08	**N**	$_{sp2}$**C–N**	399.5	1.2	0	0.2
						$_{sp2}$**C=N**	398.5	1.1	1	1
					**O**	$_{sp2}$**C=O**	532.1	2.5	0	1

*Averaged over 4 spots, standard deviation as uncertainty

Table S5. XPS parameters of DCl-COF

	Expected		XPS Survey*				Binding Energy (eV)	FWHM (eV)	Atomic Ratio	
	Atomic %	Atomic ratio	Atomic %	Atomic ratio					Expected	XPS Deconvolution
**C**	78.6	5.5	76.8±0.7	5.9±0.1	**C**	$_{sp2}$**C=C**	284.1	1.3	2.6	2.3
**N**	14.3	1	13.1±0.3	1		$_{sp2}$**C–Cl** $_{sp2}$**C=N**	285.1	1.5	1	1.0
**O**	0	0	4.1±0.7	0.2±0.05		$_{sp2}$**C=O**	286.4	1.4	0	0.3
**Cl**	7.1	0.5	6.0±0.2	0.5±0.02	**N**	$_{sp2}$**C=N**	398.2	1.2	1	1.0
					**O**	$_{sp2}$**C=O**	532.1	1.5	0	0.2

*Averaged over 3 spots, standard deviation as uncertainty

Table S6 DFT-predicted binding energy of MeO-COF fragment (B3LYP/6-31G(d)).

Element	No.	Energy (Ha)	Energy (eV)	Element	No.	Energy (Ha)	Energy (eV)	Element	No.	Energy (Ha)	Energy (eV)
C	2	–10.267	–279.58	N	10	–14.336	–389.83	O	1	–19.134	–520.12
	3	–10.207	–277.97		19	–14.330	–389.66		6	–19.192	–521.77
	4	–10.208	–278.00		27	–14.330	–389.66		24	–19.184	–521.56
	5	–10.261	–279.42		29	–14.344	–389.89		36	–19.137	–520.20
	7	–10.253	–279.21		31	–14.336	–389.83				
	8	–10.212	–278.07		38	–14.344	–389.89				
	9	–10.236	–278.56								
	11	–10.220	–278.19								
	12	–10.187	–277.30								
	13	–10.189	–277.35								
	14	–10.198	–277.61								
	15	–10.197	–277.58								
	16	–10.184	–277.22								
	17	–10.196	–277.55								
	18	–10.217	–278.11								
	20	–10.231	–278.30								
	21	–10.204	–277.91								
	22	–10.192	–277.41								
	23	–10.254	–279.04								
	25	–10.245	–278.81								
	26	–10.231	–278.30								
	28	–10.229	–278.24								
	30	–10.220	–278.19								
	32	–10.235	–278.53								
	33	–10.211	–278.05								
	34	–10.203	–277.73								
	35	–10.266	–279.56								

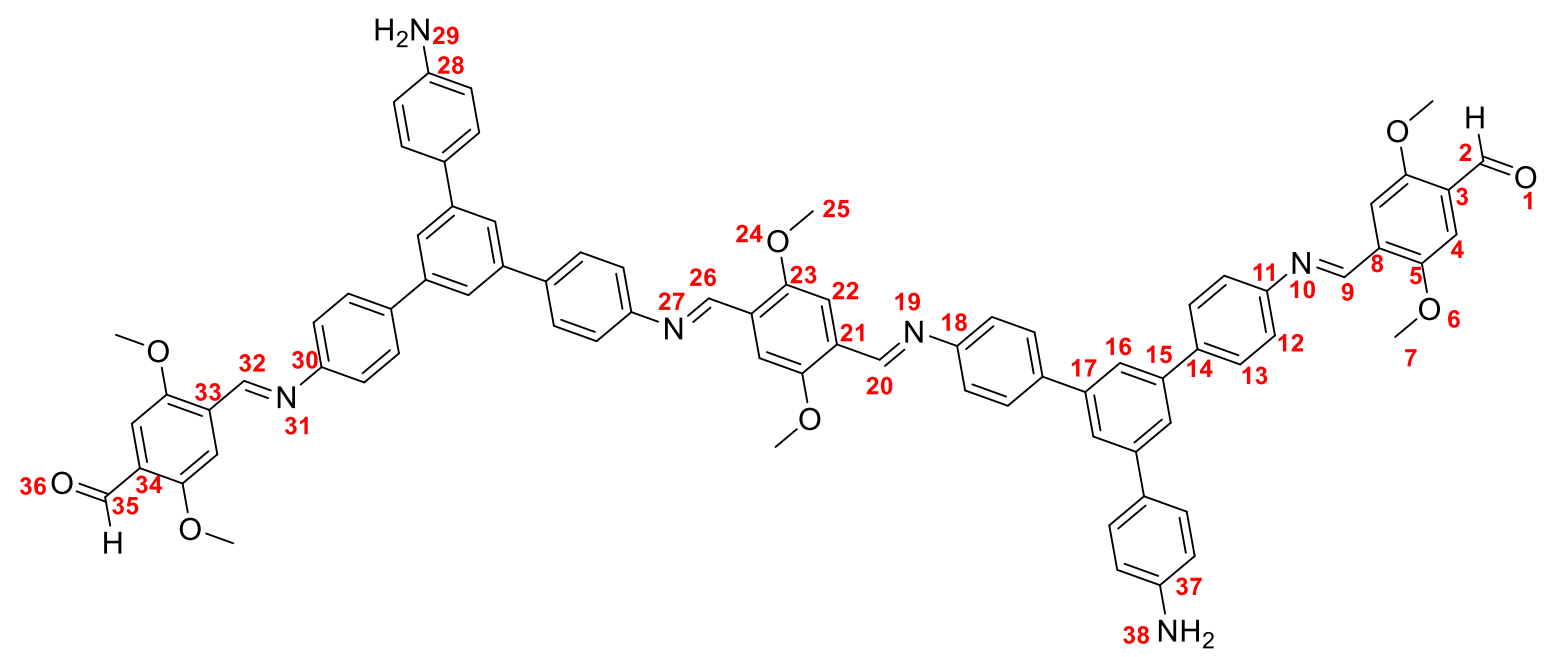

Table S7 DFT-predicted binding energy of MeS-COF fragment (B3LYP/6-31G(d)).

Element	No.	Energy (Ha)	Energy (eV)	Element	No.	Energy (Ha)	Energy (eV)	Element	No.	Energy (Ha)	Energy (eV)
C	2	–10.276	–279.54	N	10	–14.343	–389.99	O	1	–19.147	–520.41
	3	–10.213	–277.99		19	–14.338	–389.86		36	–19.147	–520.41
	4	–10.208	–277.86		27	–14.338	–389.86				
	5	–10.241	–278.66		29	–14.346	–390.06				
	7	–10.256	–279.06		31	–14.343	–389.99				
	8	–10.221	–278.19		38	–14.346	–390.06				
	9	–10.244	–278.74								
	11	–10.222	–278.21								
	12	–10.192	–277.41								
	13	–10.193	–277.44								
	14	–10.202	–277.71								
	15	–10.199	–277.63								
	16	–10.187	–277.29								
	17	–10.198	–277.61								
	18	–10.222	–278.21								
	20	–10.239	–278.60								
	21	–10.213	–277.99								
	22	–10.199	–277.63								
	23	–10.234	–278.41								
	25	–10.223	–278.24								
	26	–10.239	–278.60								
	28	–10.230	–278.30								
	30	–10.221	–278.19								
	32	–10.243	–278.71								
	33	–10.221	–278.19								
	34	–10.213	–277.99								
	35	–10.276	–279.54								
	37	–10.231	–278.33								

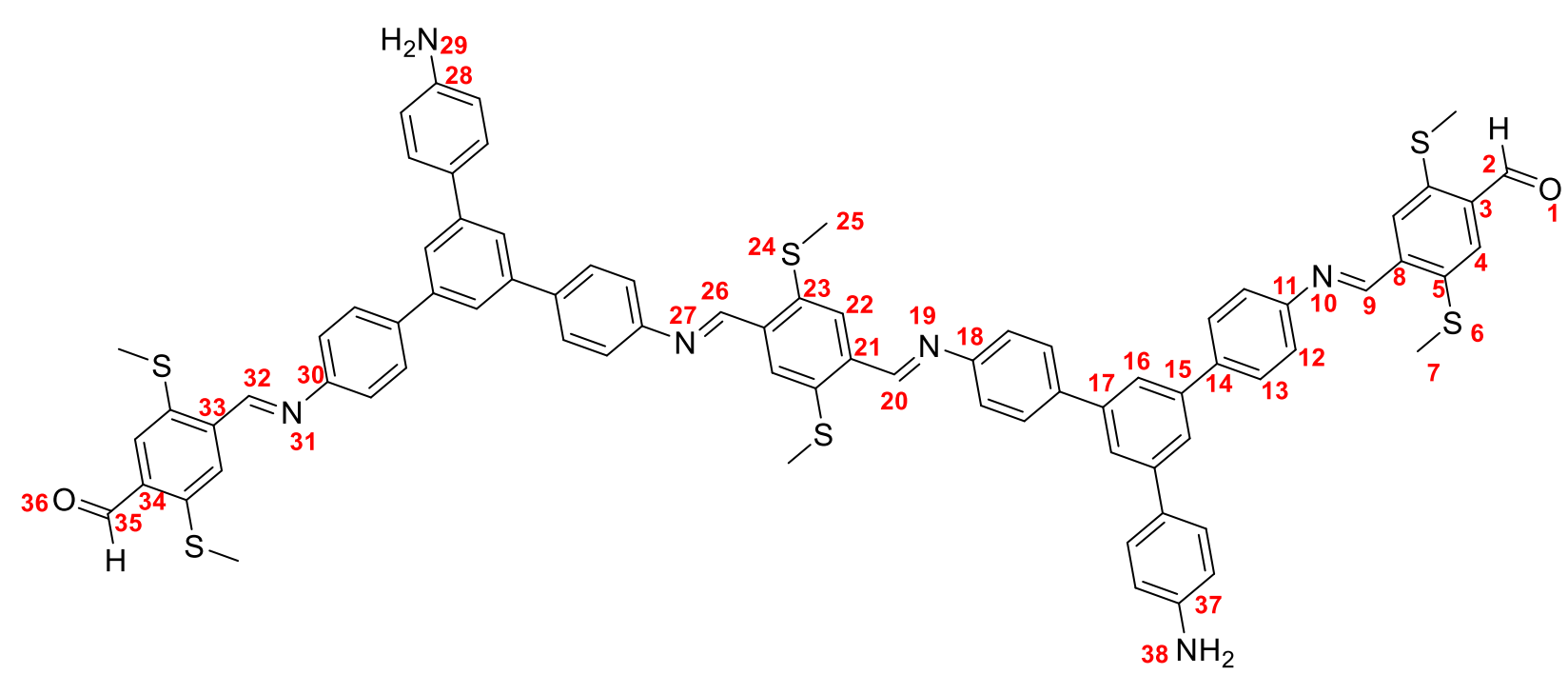

Table S8 DFT-predicted binding energy of TF-COF fragment (B3LYP/6-31G(d))

Element	No.	Energy (Ha)	Energy (eV)	Element	No.	Energy (Ha)	Energy (eV)	Element	No.	Energy (Ha)	Energy (eV)
C	2	–10.296	–280.64	N	8	–14.354	–390.33	O	1	–19.162	–520.82
	3	–10.244	–278.74		17	–14.348	–390.17		32	–19.161	–520.79
	4	–10.313	–280.99		23	–14.348	–390.17				
	5	–10.313	–280.99		25	–14.349	–390.19				
	6	–10.252	–278.96		27	–14.355	–390.36				
	7	–10.259	–279.15		34	–14.348	–390.17				
	9	–10.232	–278.32								
	10	–10.203	–277.73								
	11	–10.201	–277.68								
	12	–10.209	–277.90								
	13	–10.192	–277.41								
	14	–10.192	–277.41								
	15	–10.192	–277.41								
	16	–10.223	–278.24								
	18	–10.254	–279.01								
	19	–10.252	–278.96								
	20	–10.305	–280.78								
	21	–10.252	–278.96								
	22	–10.254	–279.01								
	24	–10.234	–278.38								
	26	–10.232	–278.32								
	28	–10.259	–279.15								
	29	–10.252	–278.96								
	30	–10.244	–278.74								
	31	–10.296	–280.64								
	33	–10.234	–278.38								

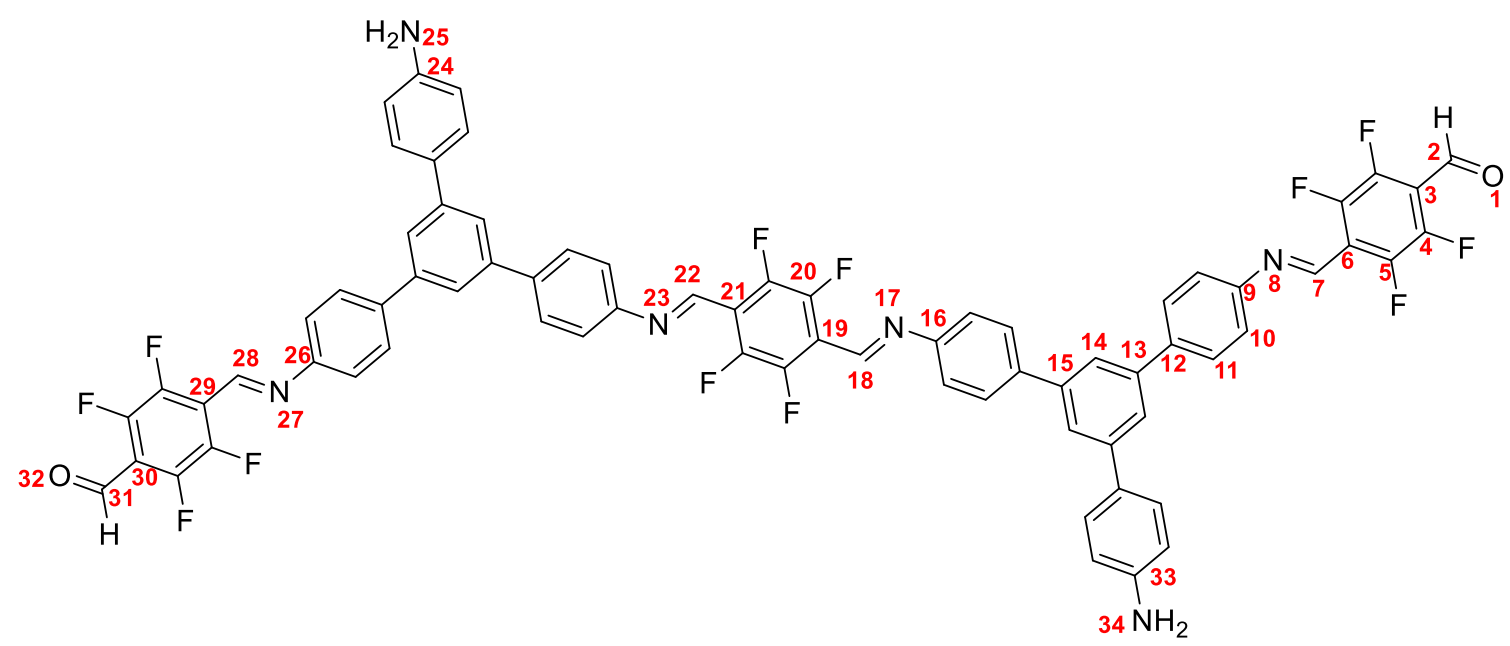

Table S9 DFT-predicted binding energy of DCl-COF fragment (B3LYP/6-31G(d)).

Element	No.	Energy (Ha)	Energy (eV)	Element	No.	Energy (Ha)	Energy (eV)	Element	No.	Energy (Ha)	Energy (eV)
C	2	–10.261	–279.28	N	1	–14.365	–390.91	O	25	–19.151	–520.90
	3	–10.211	–277.93		6	–14.347	–390.40		34	–19.151	–520.90
	4	–10.260	–279.25		13	–14.351	–390.51				
	5	–10.239	–278.67		17	–14.351	–390.51				
	7	–10.247	–278.88		21	–14.351	–390.51				
	8	–10.215	–277.89		28	–14.345	–390.35				
	9	–10.209	–277.77		31	–14.351	–390.51				
	10	–10.206	–277.69								
	11	–10.215	–277.89								
	12	–10.289	–280.36								
	14	–10.289	–280.36								
	15	–10.217	–277.94								
	16	–10.254	–278.99								
	18	–10.248	–278.90								
	19	–10.212	–277.91								
	20	–10.264	–279.25								
	22	–10.254	–278.99								
	23	–10.213	–277.94								
	24	–10.280	–279.65								
	26	–10.214	–277.97								
	27	–10.246	–278.88								
	29	–10.239	–278.67								
	30	–10.257	–279.12								
	32	–10.214	–277.97								
	33	–10.280	–279.65								

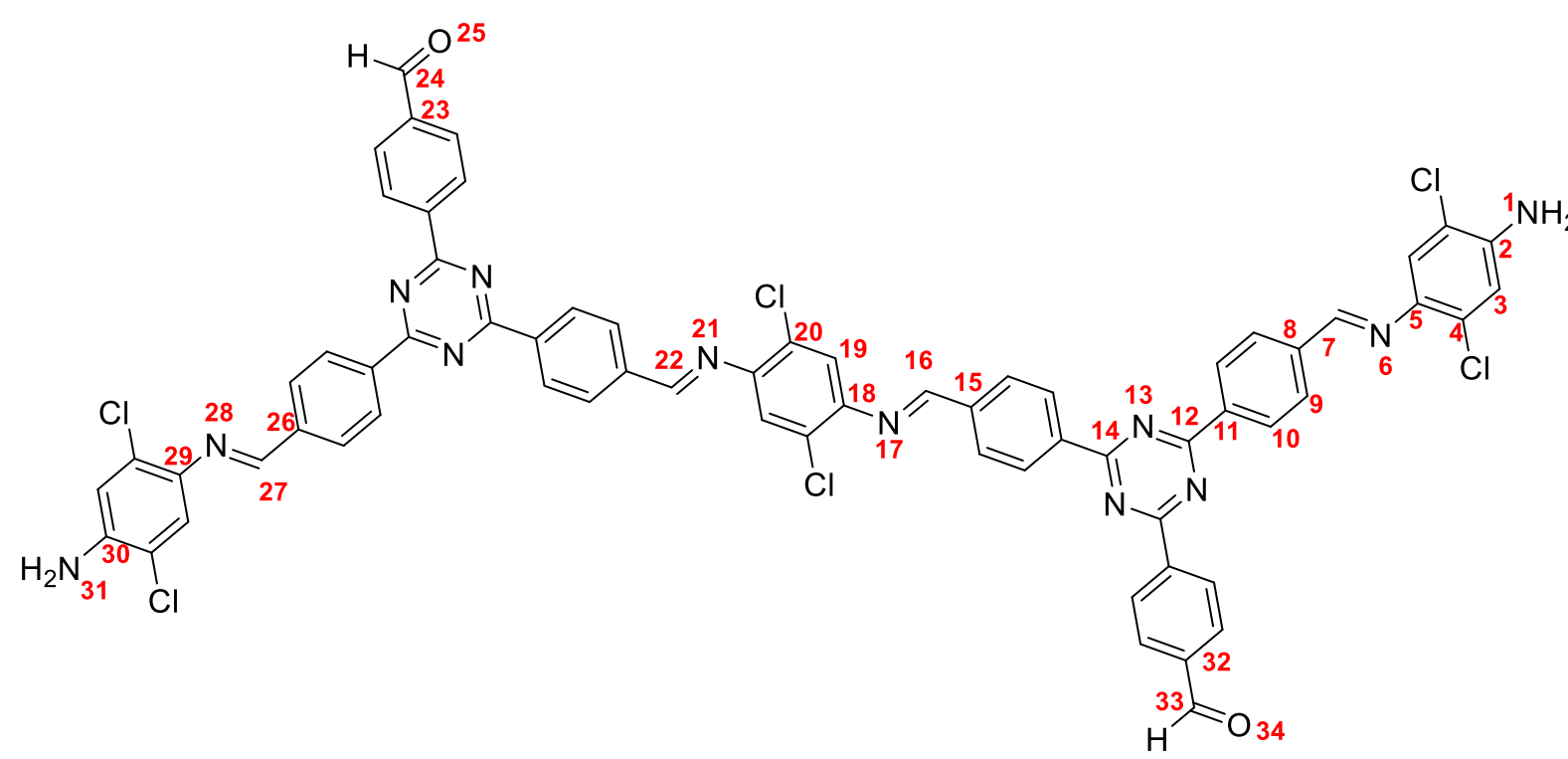

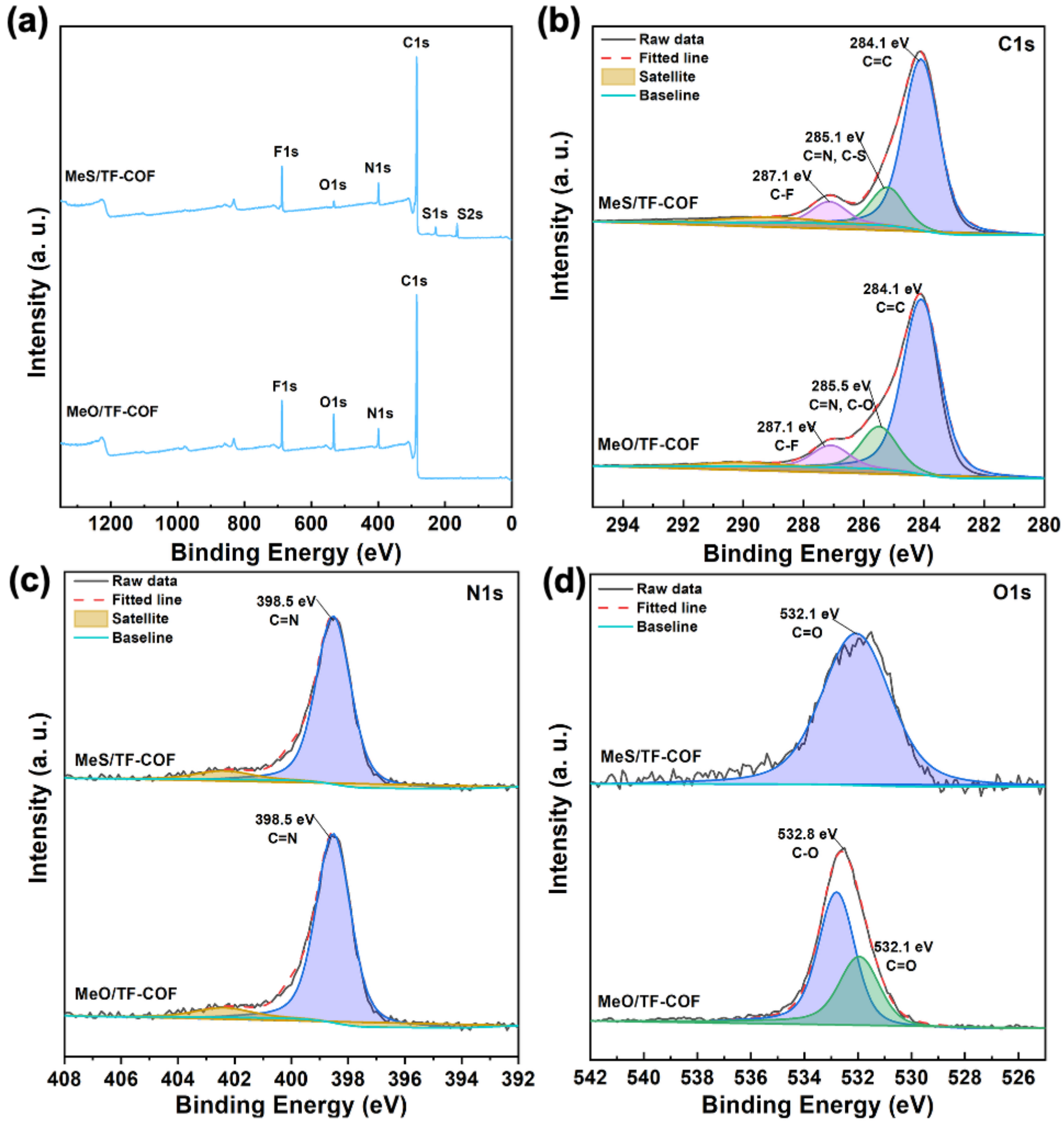

Figure S22 High-resolution XPS (a) survey, (b) C 1s, (c) N 1s, and (d) O 1s spectra of MeO/TF-COF and MeS/TF-COF.

Table S10. XPS parameters of MeO/TF-COF

	**Expected** if aldehyde 50-50% mixed (C76F4N6O4 + C74F8N6O2 = C150F12N12O6)		**XPS Survey***				**Binding Energy (eV)**	**FWHM (eV)**	**Atomic Ratio**	
	Atomic %	Atomic ratio	Atomic %	Atomic ratio					Expected	XPS Deconvolution
**C**	83.3	12.6	82.5±2.0	14.2±3.9	**C**	**sp2C=C**	284.1	1.5	9.5	8.3
**N**	6.6	1	4.9±1.2	0.84±0.31		**sp2C–O**<br>**sp2C=N**	285.5	1.4	2	2
**O**	3.3	0.5	6.8±0.8	1.17±0.35		**sp2C=O**<br>**sp2C–F**	287.1	1.5	1	1
**F**	6.6	1	5.8±1.6	1	**N**	**sp2C–N**			0	0
						**sp2C=N**	398.5	1.6	1	1
					**O**	**sp2C=O**	532.1	1.6	0	0.6
						**sp2C–O**	532.8	1.8	1	1

*Averaged over 4 spots, standard deviation as uncertainty.

Table S11. XPS parameters of MeS/TF-COF

	**Expected** if aldehyde 50-50% mixed ($C_{76}F_4N_6S_4$ + $C_{74}F_8N_6S_2$ = $C_{150}F_{12}N_{12}S_6$)		**XPS Survey***				**Binding Energy (eV)**	**FWHM (eV)**	**Atomic Ratio**	
	Atomic %	Atomic ratio	Atomic %	Atomic ratio					Expected	XPS Deconvolution
**C**	83.3	12.6	82.3±0.1	13.9±0.1	**C**	$_{sp2}$**C=C**	284.1	1.4	9.5	8.3
**N**	6.6	1	5.9±0.4	1		$_{sp2}$**C=N**<br>$_{sp3}$**C–S**	285.1	1.3	2.0	1.8
**O**	0	0	2.1±0.3	0.4±0.1		$_{sp2}$**C–F**<br>$_{sp2}$**C=O**	287.1	1.4	1.0	1.0
**F**	6.6	1	6.6±0.2	1.1±0.1	**N**	$_{sp2}$**C=N**	398.5	1.5	1	1
**S**	3.3	0.5	3.1±0.1	0.5±0.04	**O**	$_{sp2}$**C=O**	532.1	3.0	0	1.0

*Averaged over 4 spots, standard deviation as uncertainty.

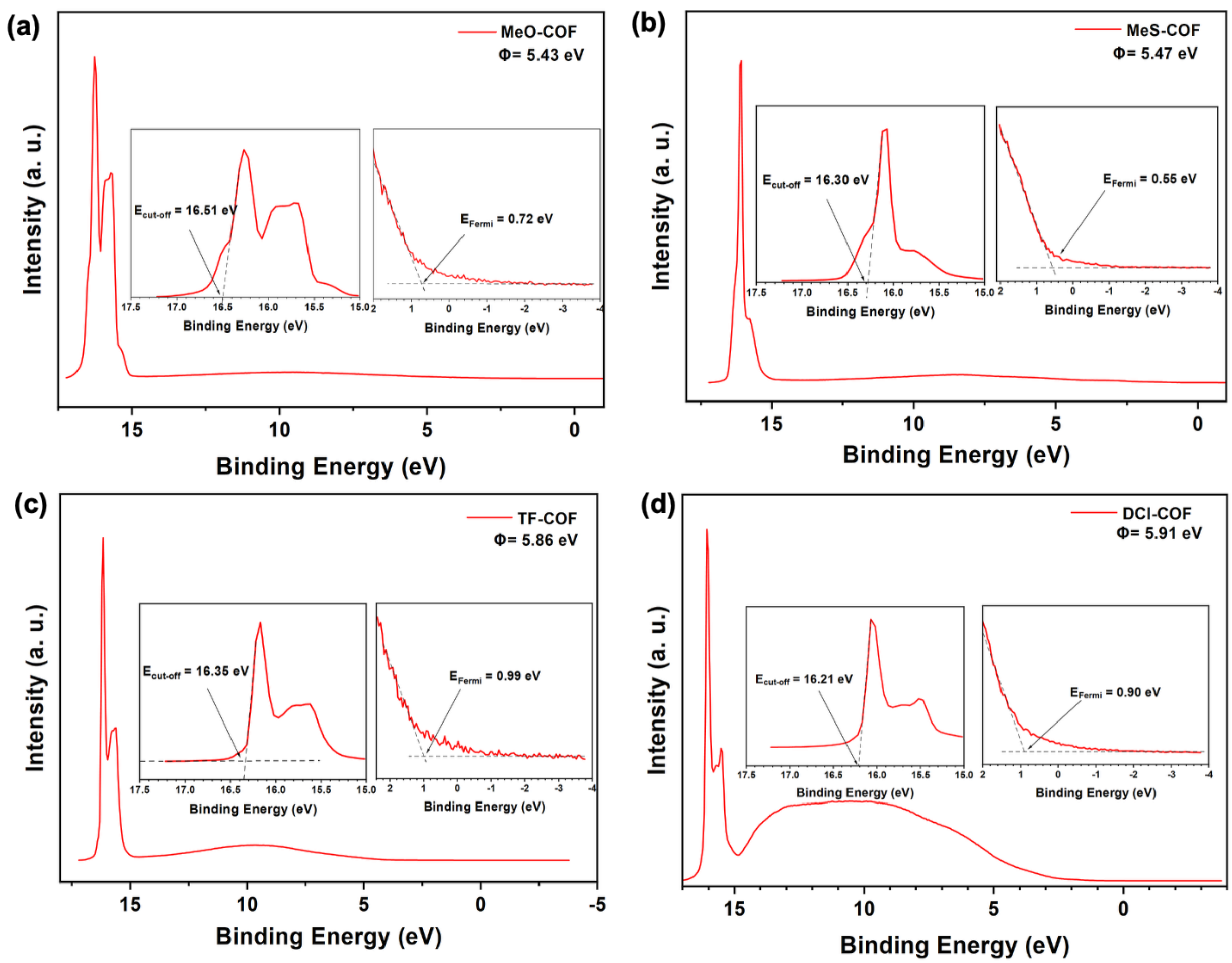

Figure S23 UPS spectra of MeO-COF, MeS-COF, TF-COF and DCl-COF.

6. Characterization of Core-Shell and Mixed D/A COFs

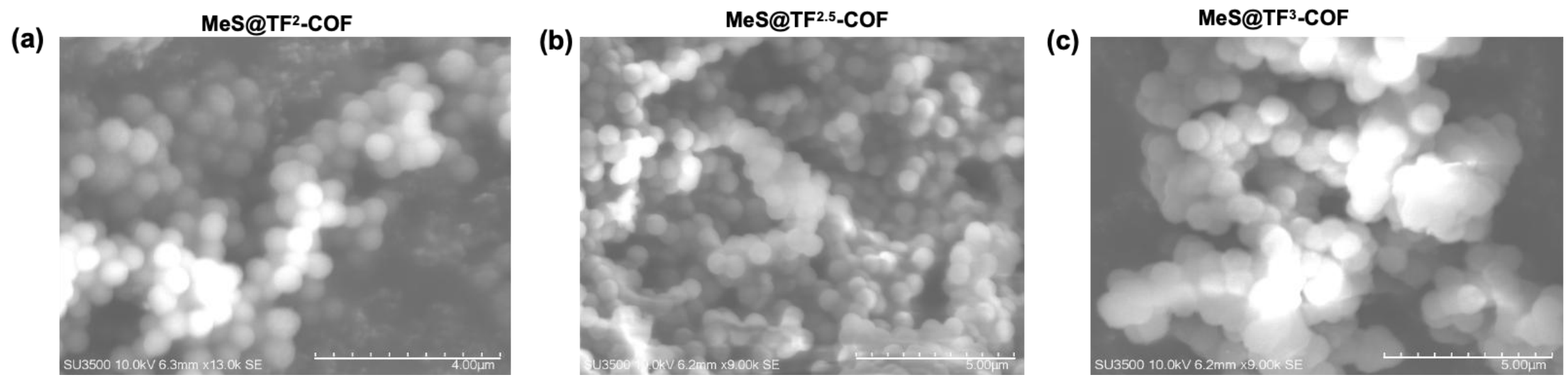

Figure S24 SEM image of (a) MeS@TF^{2}-COF, (b) MeS@TF$^{2.5}$-COF and (c) MeS@TF^{3}-COF

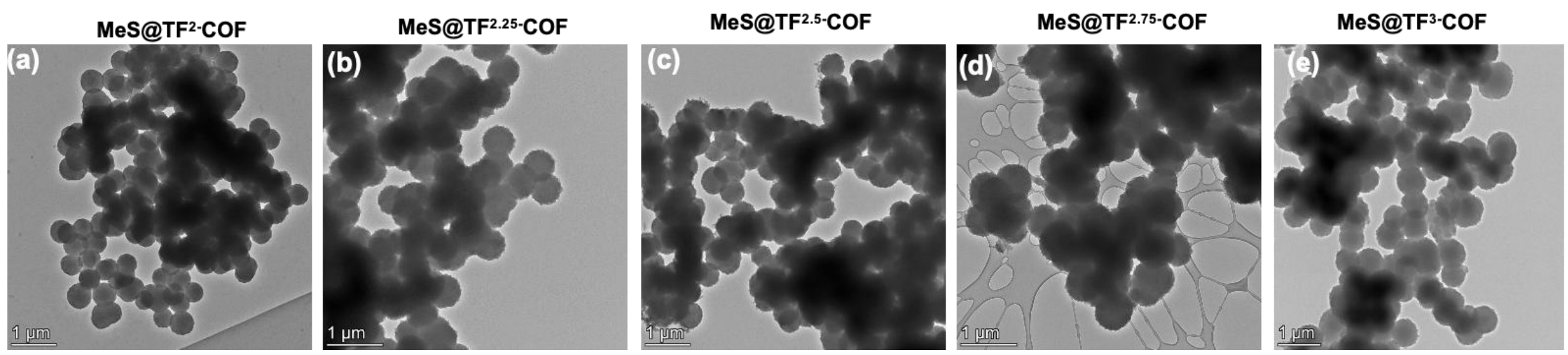

Figure S25 TEM image of (a) MeS@TF^{2}-COF, (b) MeS@TF$^{2.25}$-COF, (c) MeS@TF$^{2.5}$-COF (d) MeS@TF$^{2.75}$-COF, and (e) MeS@TF^{3}-COF.

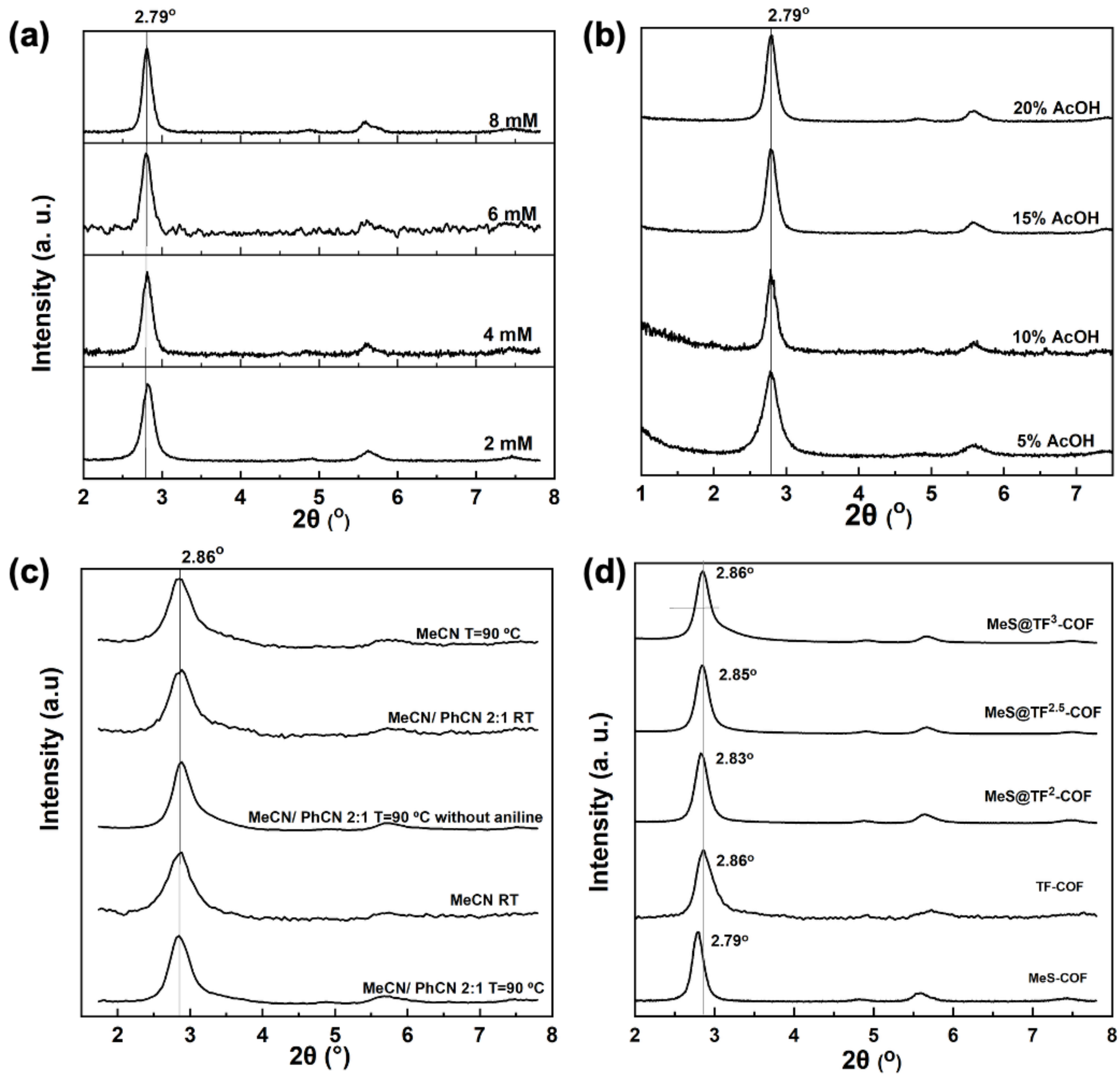

Figure S26 SAXS pattern of (a, b) MeS-COF with different concentration of TAPB monomer (a), and different amount of AcOH catalyst (b); (c) TF-COF with different solvents and temperatures; (d) MeS@TF-COFs with different shell thickness compared to pure MeS-COF and TF-COF.

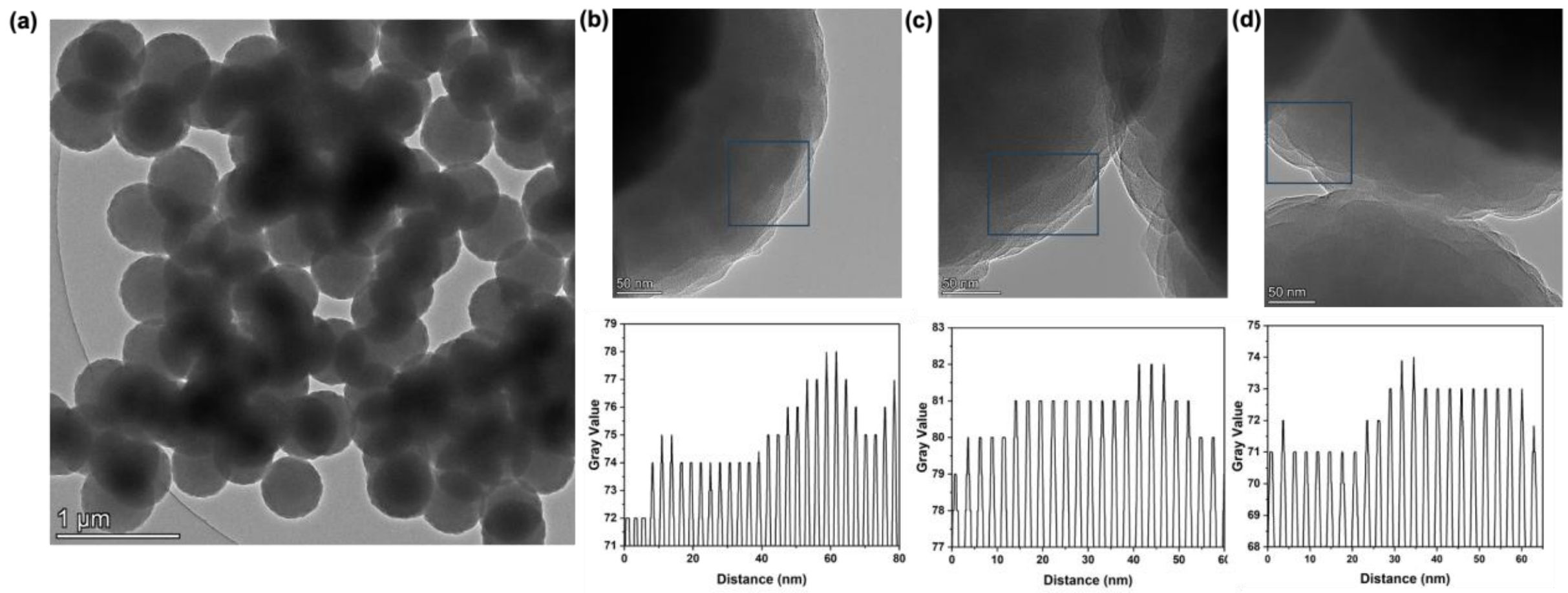

Figure S27 TEM image of MeS@TF$^{2.5}$-COF, and (b-d) HR-TEM image (top) and corresponding line intensity profile (bottom).

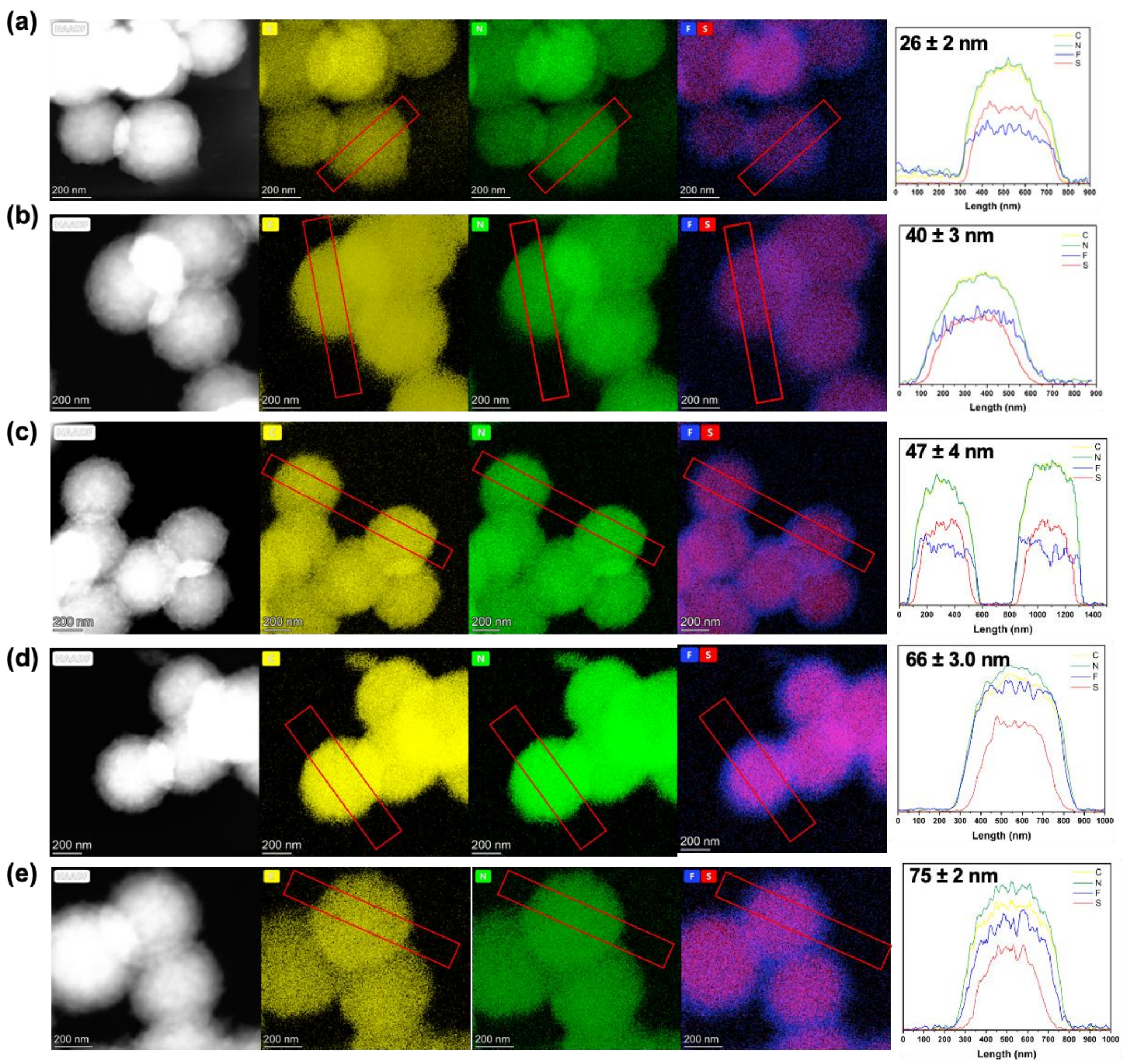

Figure S28 The HAADF-TEM image, EDS elemental mapping and related profile lines of (a) MeS@TF^{2}-COF, (b) MeS@TF$^{2.25}$-COF, (c) MeS@TF$^{2.5}$-COF (d) MeS@TF$^{2.75}$-COF, and (e) MeS@TF^{3}-COF.

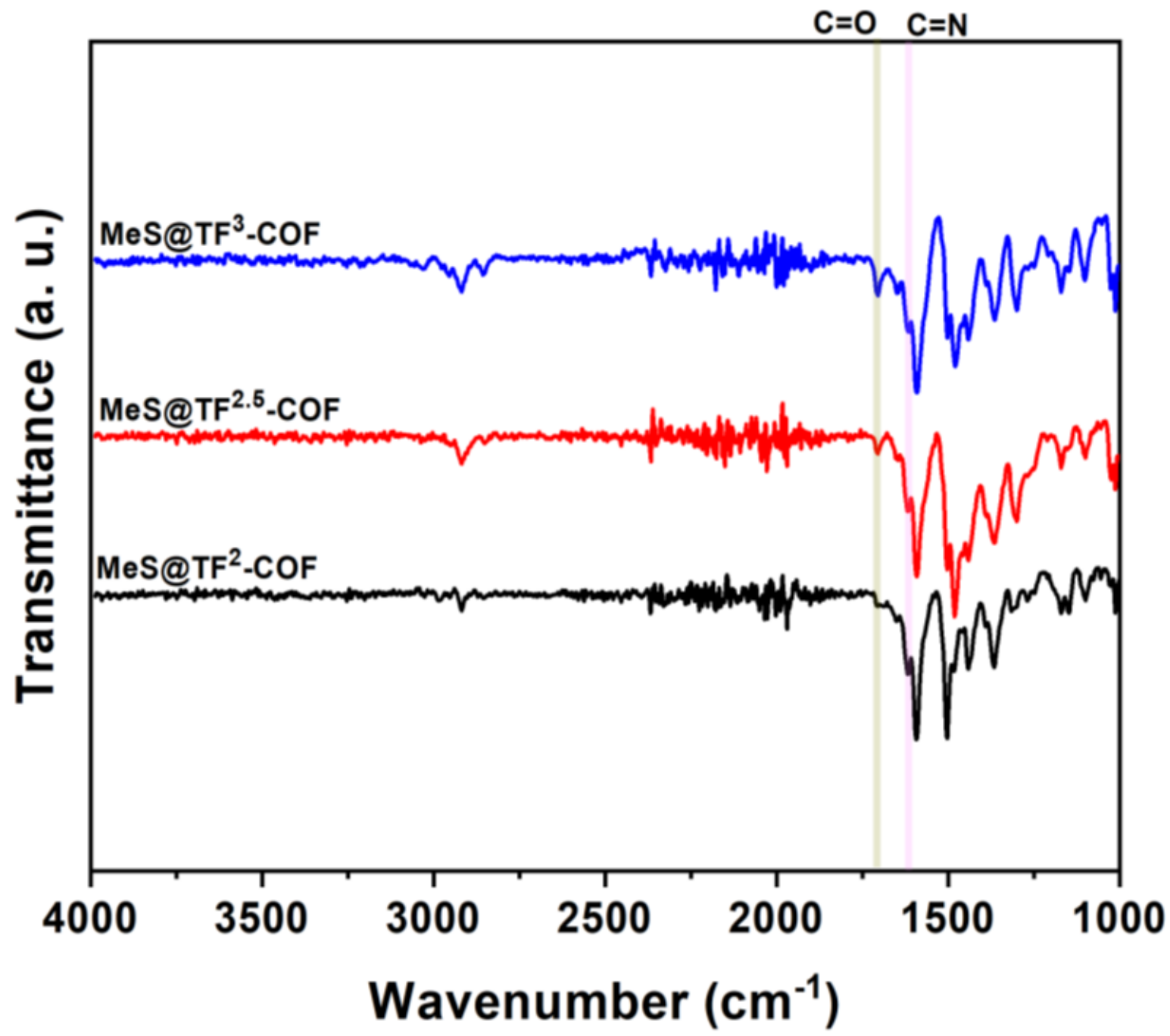

Figure S29 FTIR spectra of core-shell MeS@TF-COFs.

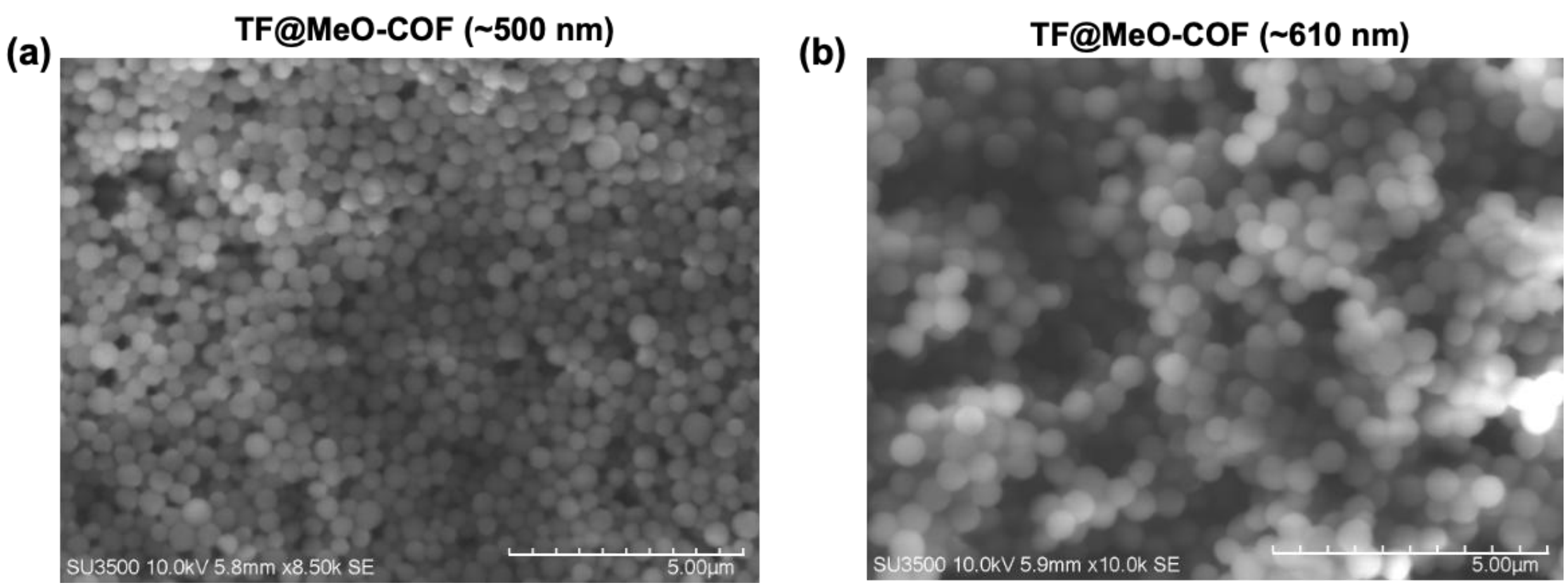

Figure S30 SEM images of MeO@TF^2-COF and MeO@TF^3-COF.

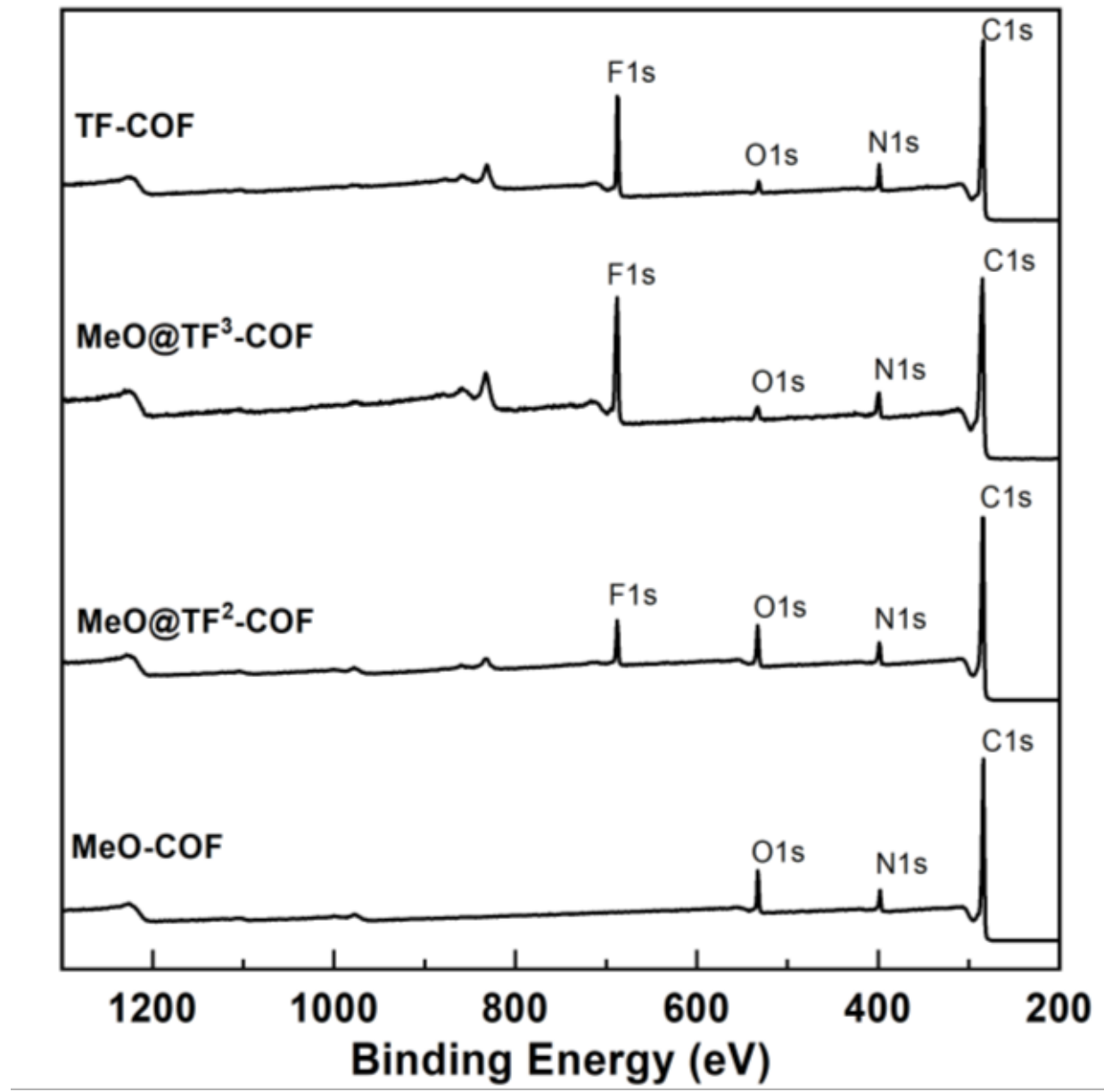

Figure S31 XPS spectra of MeO-COF, MeO@TF-COFs and TF-COF.

Table S12 XPS parameters of MeO-COF, MeO@TF-COFs and TF-COF.

XPS	MeO-COF		MeO@TF²-COF		MeO@TF³-COF		TF-COF	
	Atomic %	Atomic ratio	Atomic %	Atomic ratio	Atomic %	Atomic ratio	Atomic %	Atomic ratio
C	85.5±0.5	17.1±2.8	82.2±0.9	15.5±1.6	76.8±0.3	14.0±0.6	78.8±0.5	13.6±0.8
N	5.0±0.8	1	5.3±0.5	1	5.5±0.2	1	5.8±0.3	1
O	9.5±1.2	1.9±0.5	7.4±1.0	1.4±0.3	2.7±0.2	0.5±0.05	1.9±0.4	0.3±0.08
F			5.2±0.7	1.0±0.2	15.0±0.6	2.7±0.2	13.5±0.5	2.3±0.2

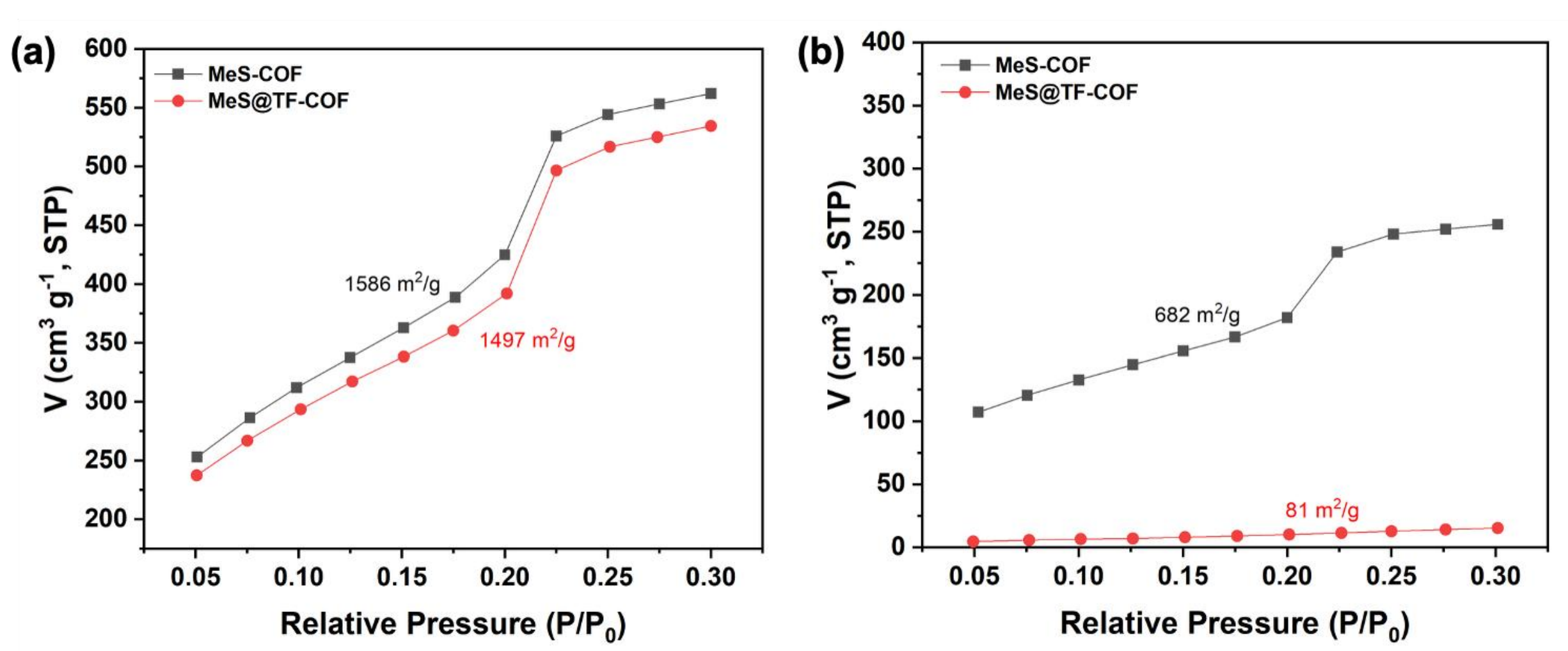

Figure S32 N_2 sorption isotherm curves of MeS-COF and MeS@TF$^{2.5}$-COF measured for samples activated after (a) and before (b) the solvent exchange, and at 77 K.

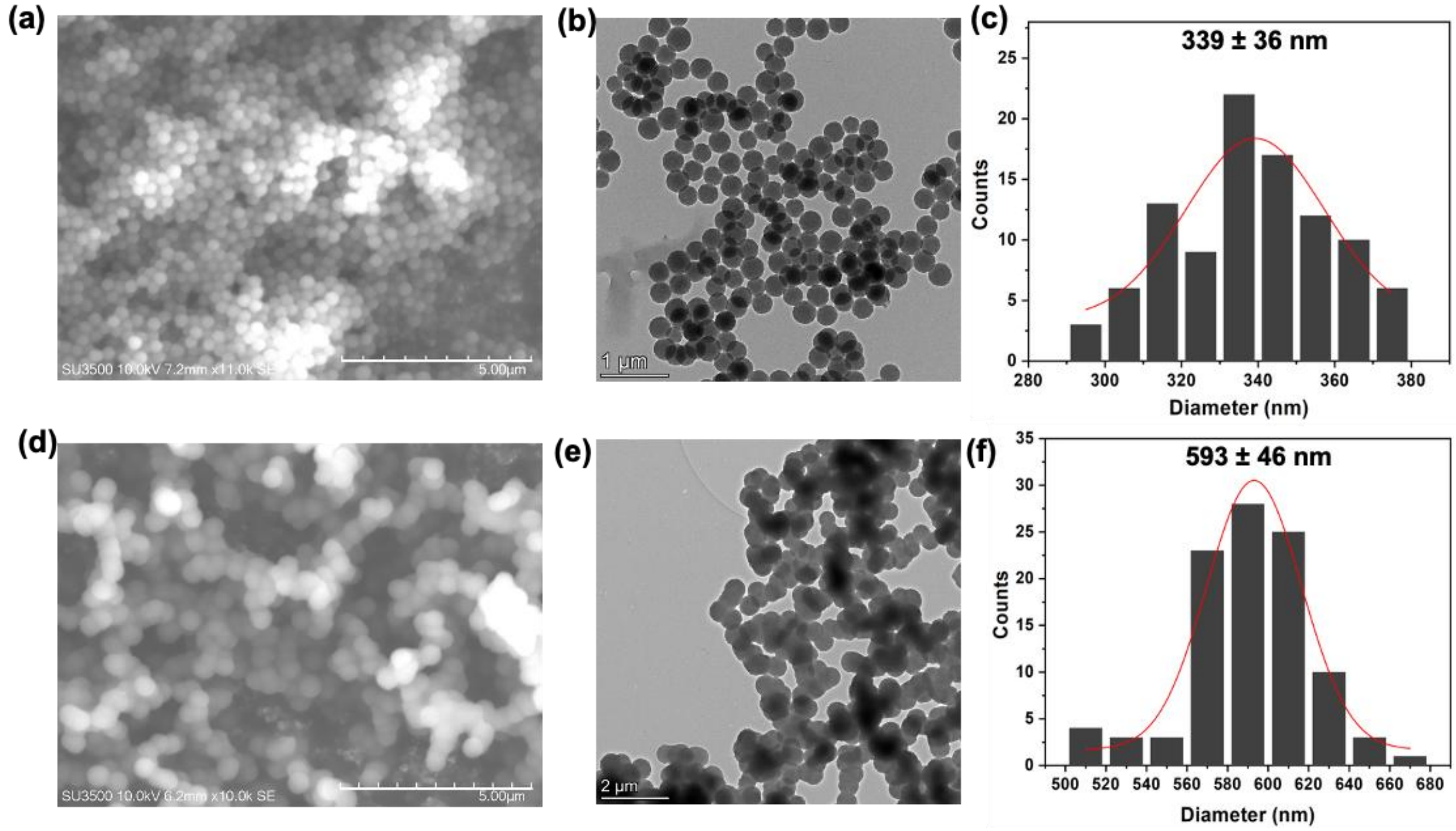

Figure S33 SEM image, TEM image and related particle size histogram of (a-c) MeO/TF-COF and (d-f) MeS/TF-COF.

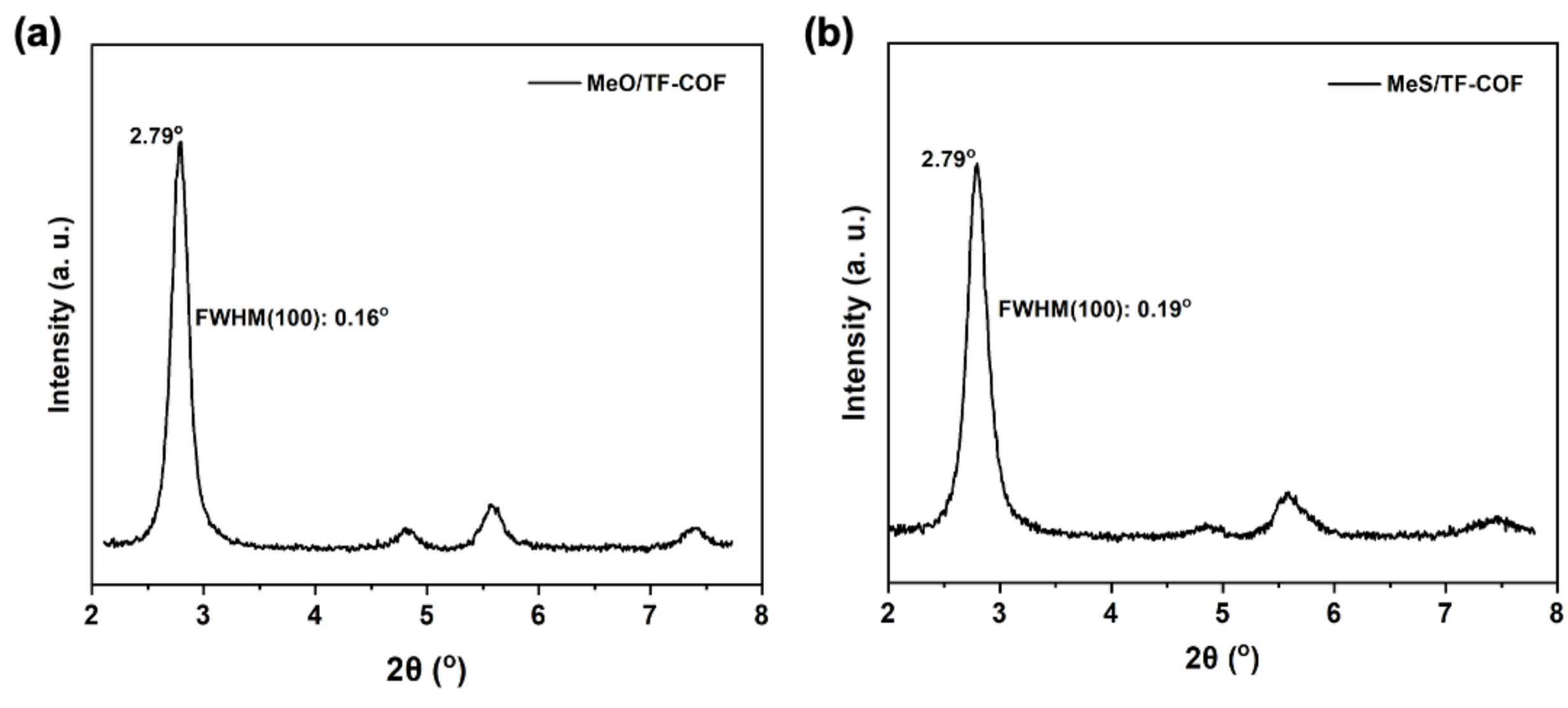

Figure S34 SAXS pattern of (a) MeO/TF-COF and (b) MeS/TF-COF.

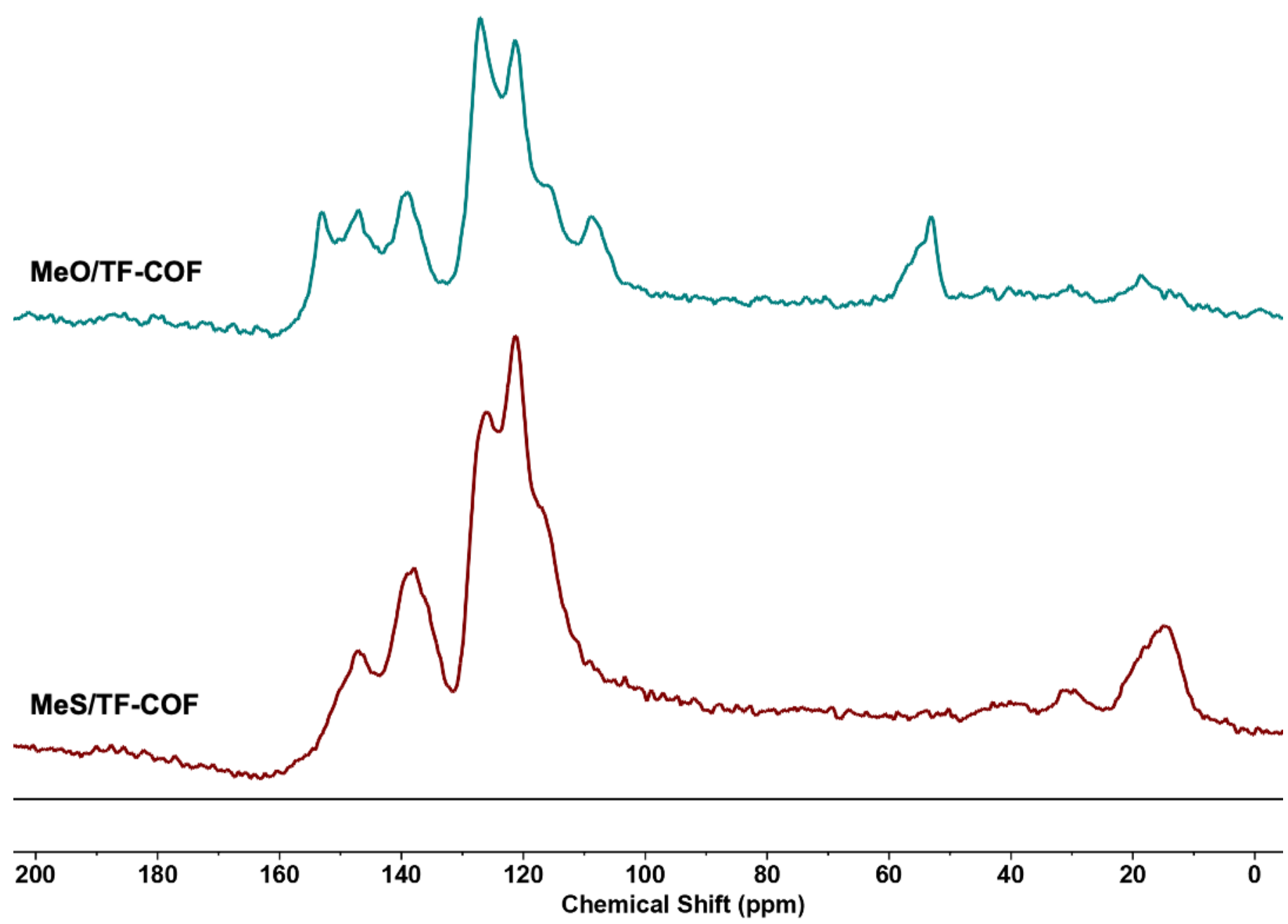

Figure S35 Solid-state ^{13}C-NMR spectra of MeO/TF-COF and MeS/TF-COF.

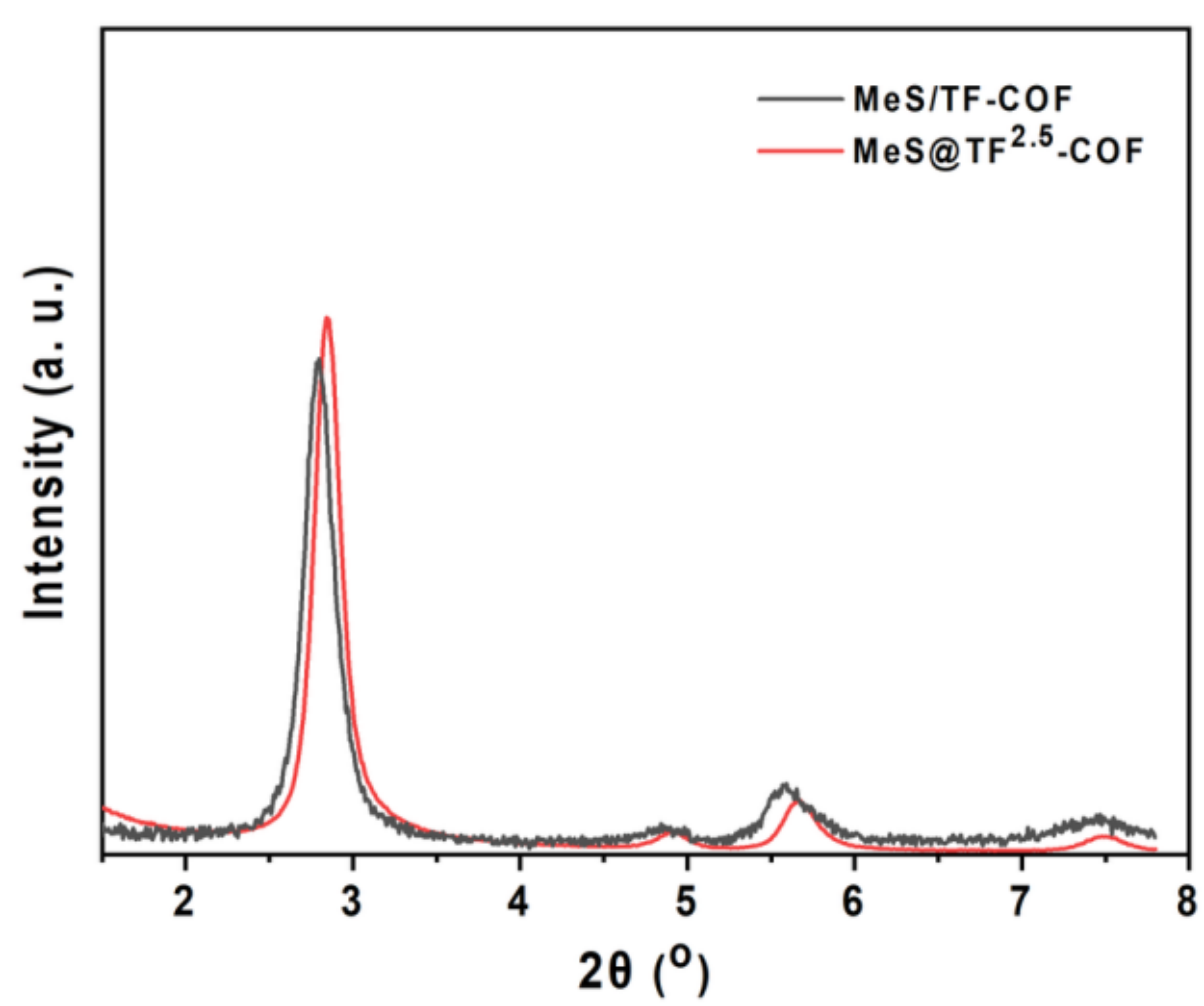

Figure S36. The comparison of the SAXS patterns of MeS/TF-COF and MeS@TF$^{2.5}$-COF.

7. Optical Characterization of COF NPs

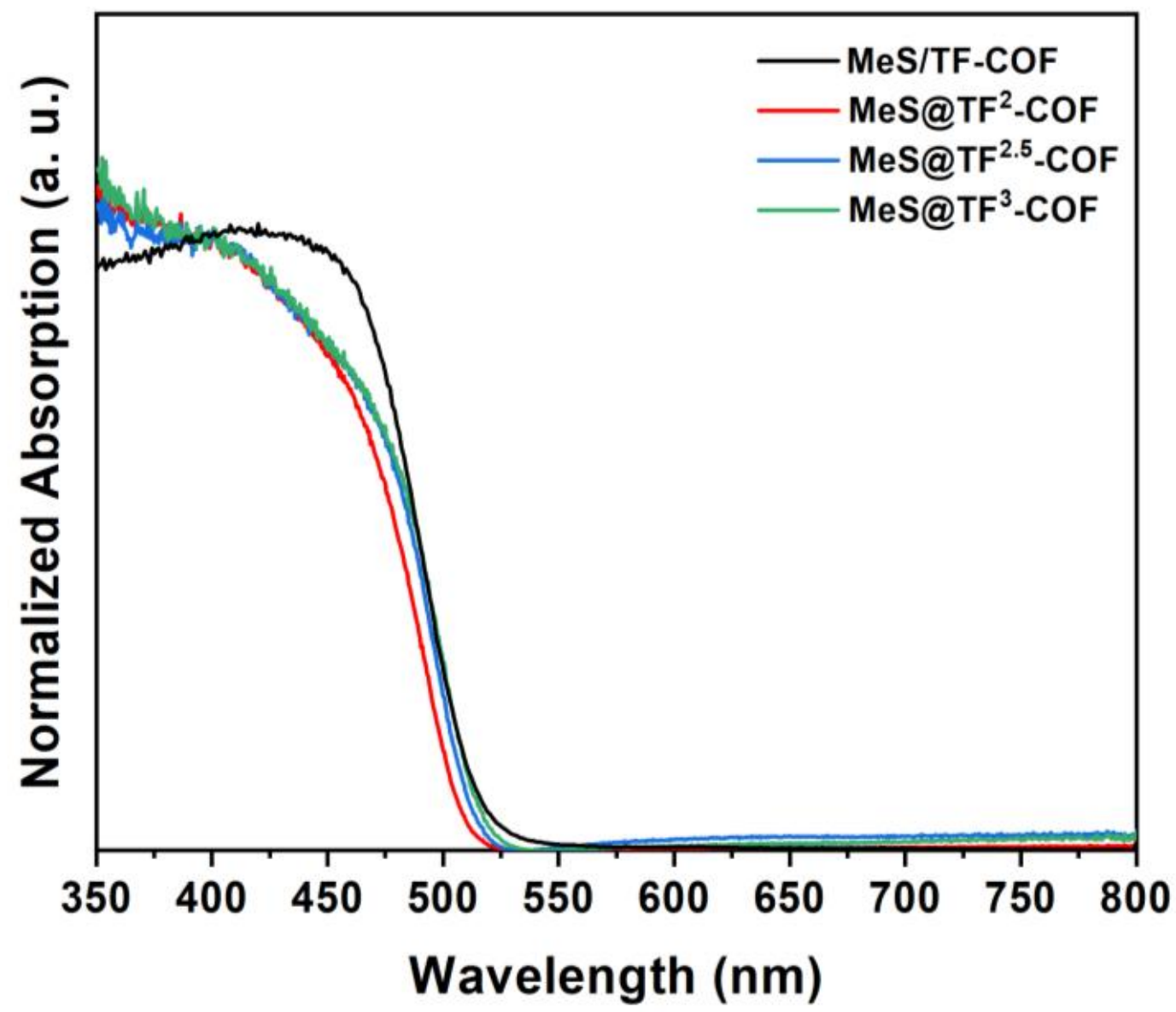

Figure S37 Normalized absorption from DRS spectra of MeS/TF-COF and MeS@TF-COFs.

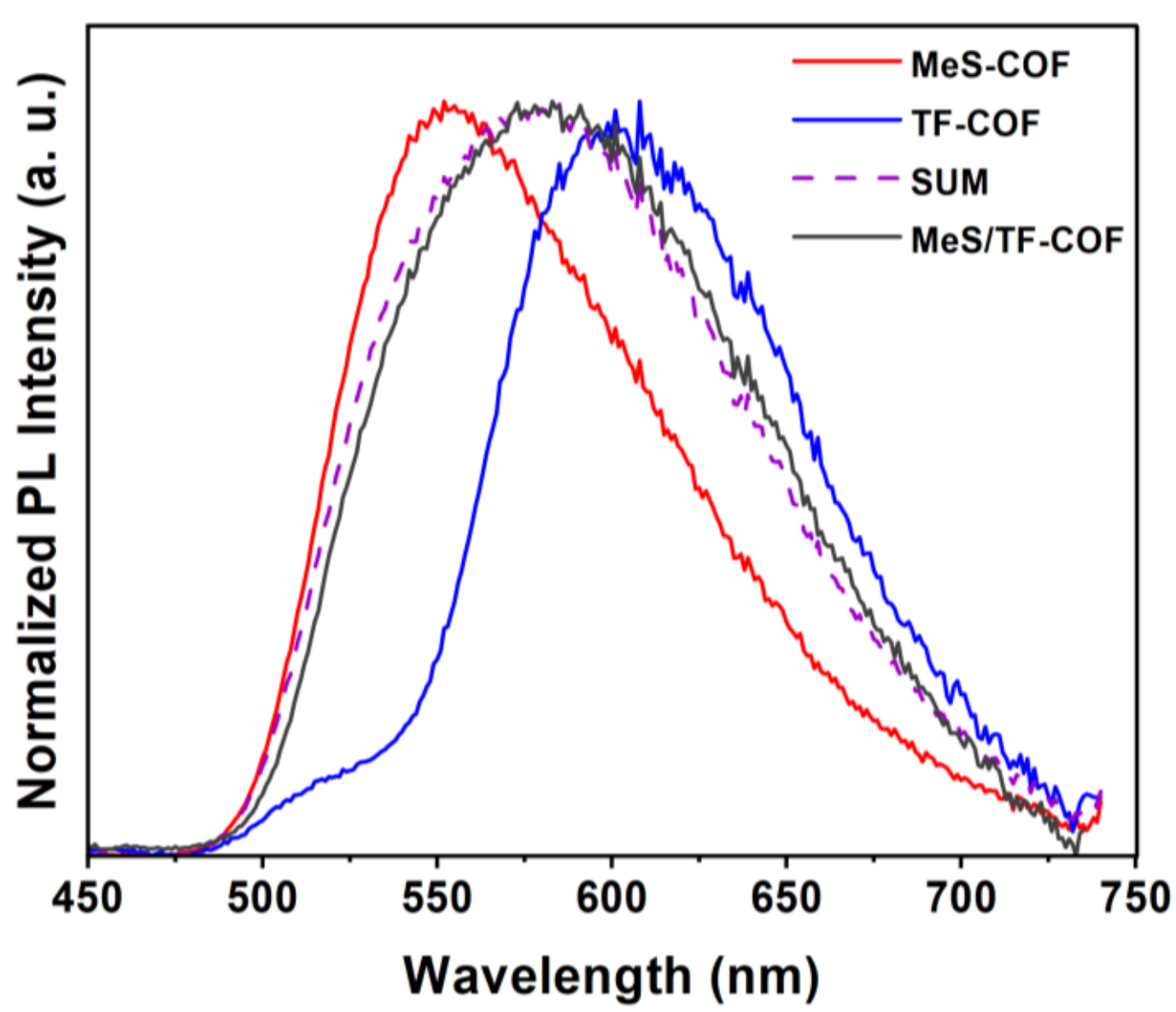

Figure S38 Normalized PL spectra of MeS-COF, TF-COF and MeS/TF-COF, and the sum of the individual MeS-COF and TF-COF PL spectra.

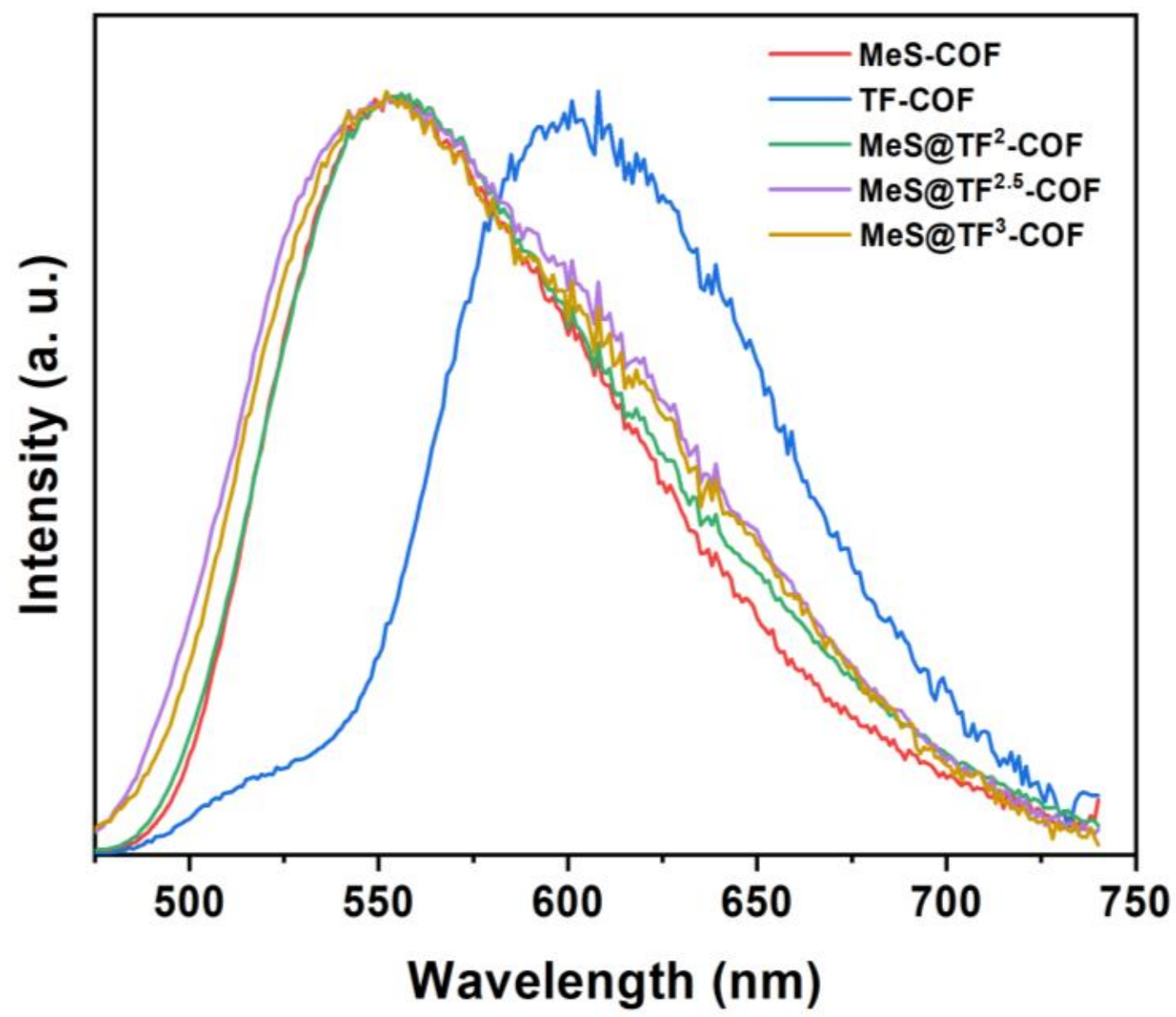

Figure S39 Normalized PL spectra of MeS-COF, TF-COF and MeS@TF-COF heterojunctions.

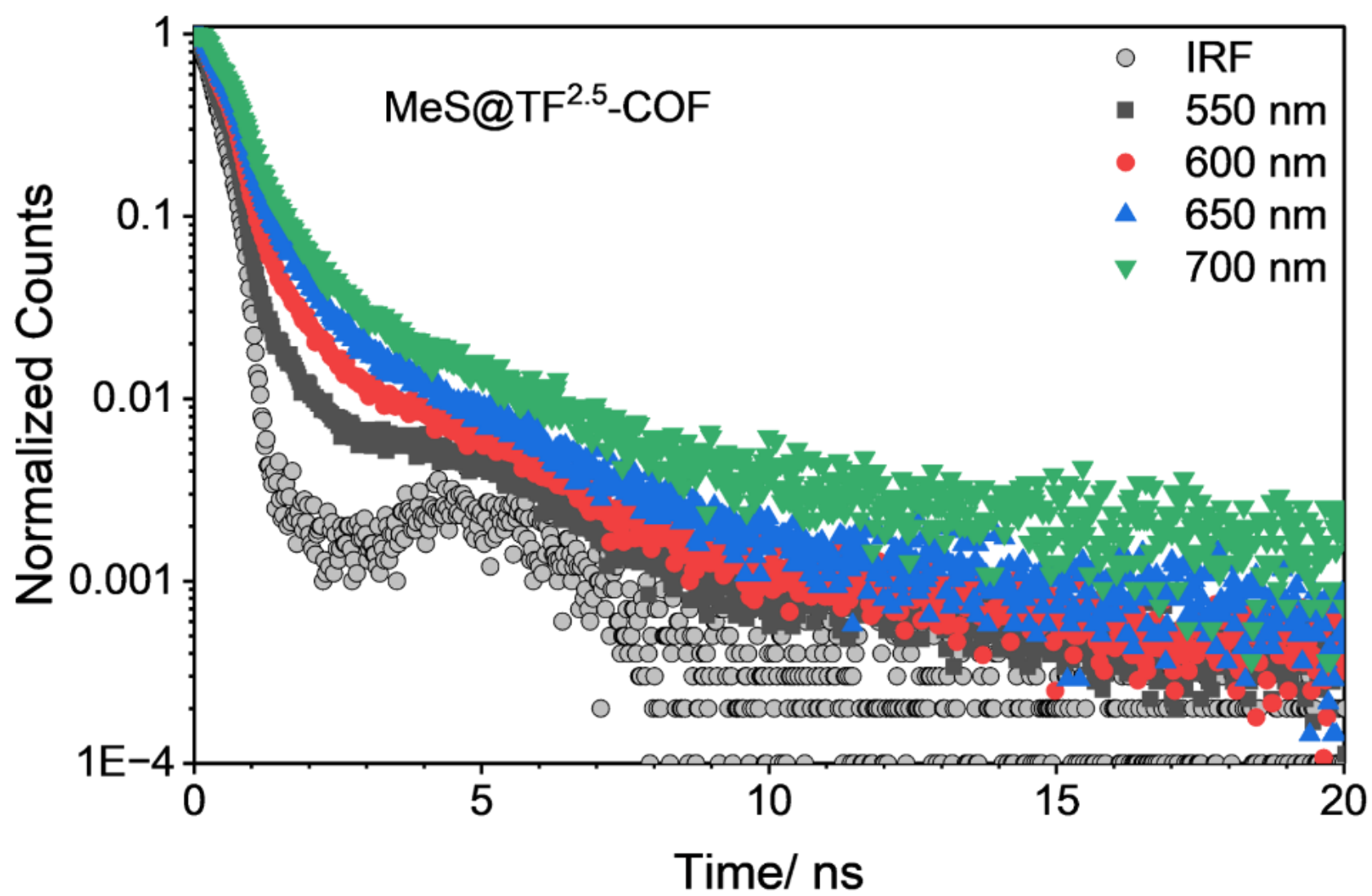

Figure S40 Normalized PL decay traces of MeS@TF$^{2.5}$-COF taken at wavelengths corresponding to contributions from MeS-COF (550 nm), TF-COF (700 nm) and in between.

Exciton Diffusion Length:

Excitation diffusion lengths were determined using the method detailed by Gommans and co-workers.[3] The quenched PL lifetimes of the core-shell COFs (Fig. 5b) relative to that of pure TF-COF were used to calculate a quenching constant ($Q = (\tau_0-\tau)/\tau_0$) which was seen to diminish with the increment of shell thickness. This behaviour was fit with a probabilistic function (**Equation S1**) meant to describe the probability of an exciton's quenching as a factor of diffusion length (L_D), intrinsic PL lifetime (τ_0) and distance from the quenching interface (x). As the shell thickness and absorptivity were low enough in all cases to show negligible extinction through the material, we assumed a uniform density of excitons being generated with an average position of halfway through the shell's thickness (L/2). [3, 4].

$$P(x) = 1 - \mathrm{erf}(x/\sqrt{2}L_D) \qquad \text{(S1)}$$

Here, $P(x)$ describes the probability that an exciton generated at a distance x from the absorbing interface will reach the interface by random diffusion and be quenched before it decays, erf is the error function, and L_D is the one-dimensional exciton diffusion length.

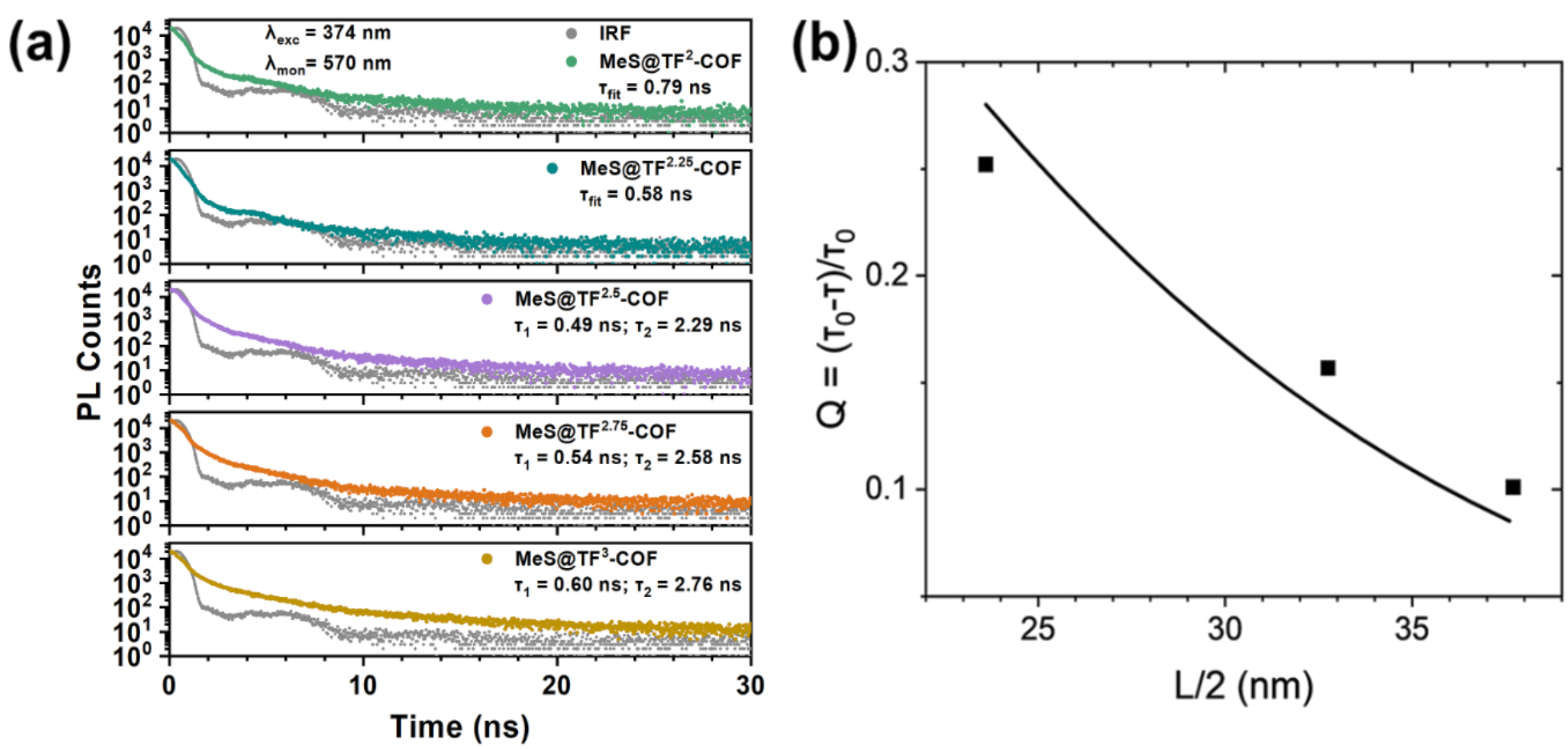

Figure S41 (a) PL decay traces for MeS@TF-COF NPs. (b) PL lifetime quenching efficiency (Q) as a factor of half-shell-thickness (L/2) for MeS@TF$^{2.5}$-COF, MeS@TF$^{2.75}$-COF and MeS@TF^{3}-COF, with probability fit from which the exciton diffusion length (L_D) is derived. The decays of MeS@TF^{2}-COF and MeS@TF$^{2.25}$-COF were too rapid to be reliably deconvolved from our instrument's response function (IRF) and were omitted.

8. EPR Characterization of COF NPs

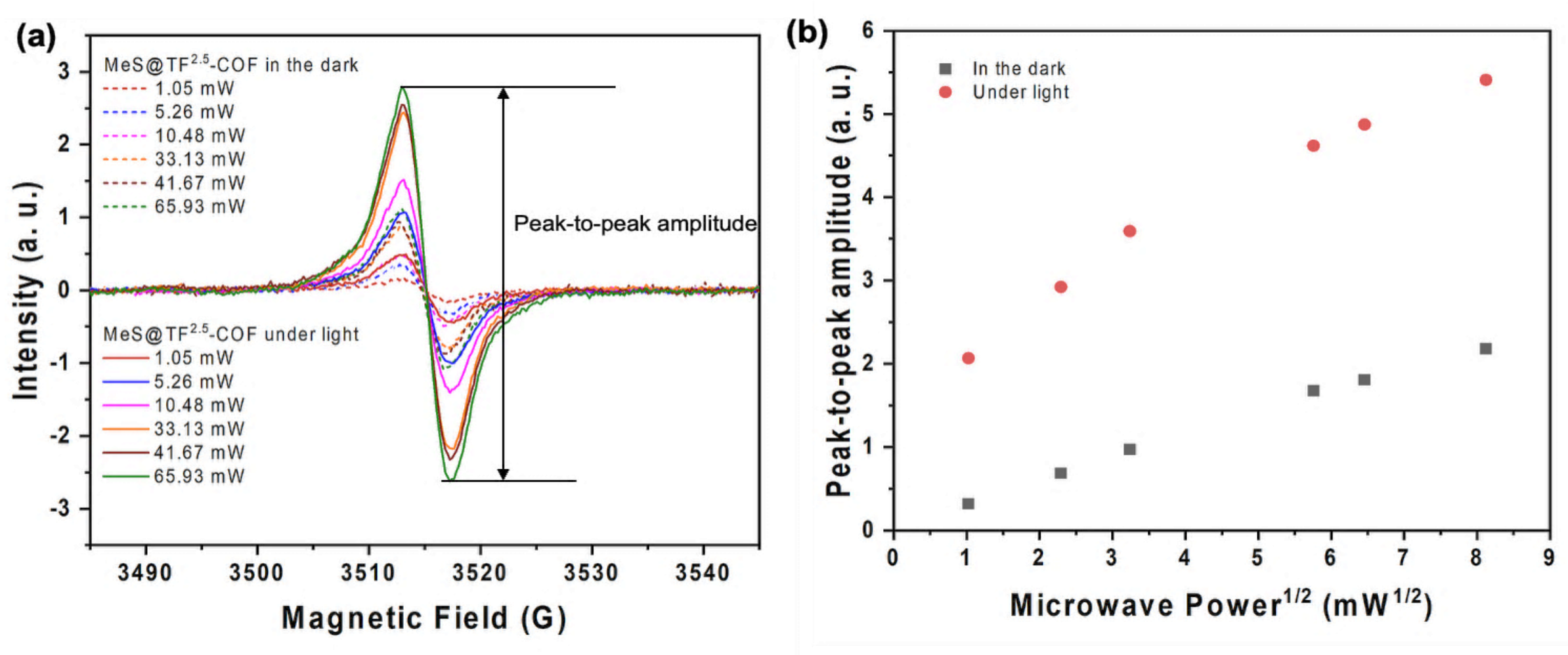

Figure S42 (a) Microwave power dependence of the EPR spectra of MeS@TF$^{2.5}$-COF recorded in the dark and under illumination. (b) Corresponding peak-to-peak amplitudes as a function of the square root of microwave power.

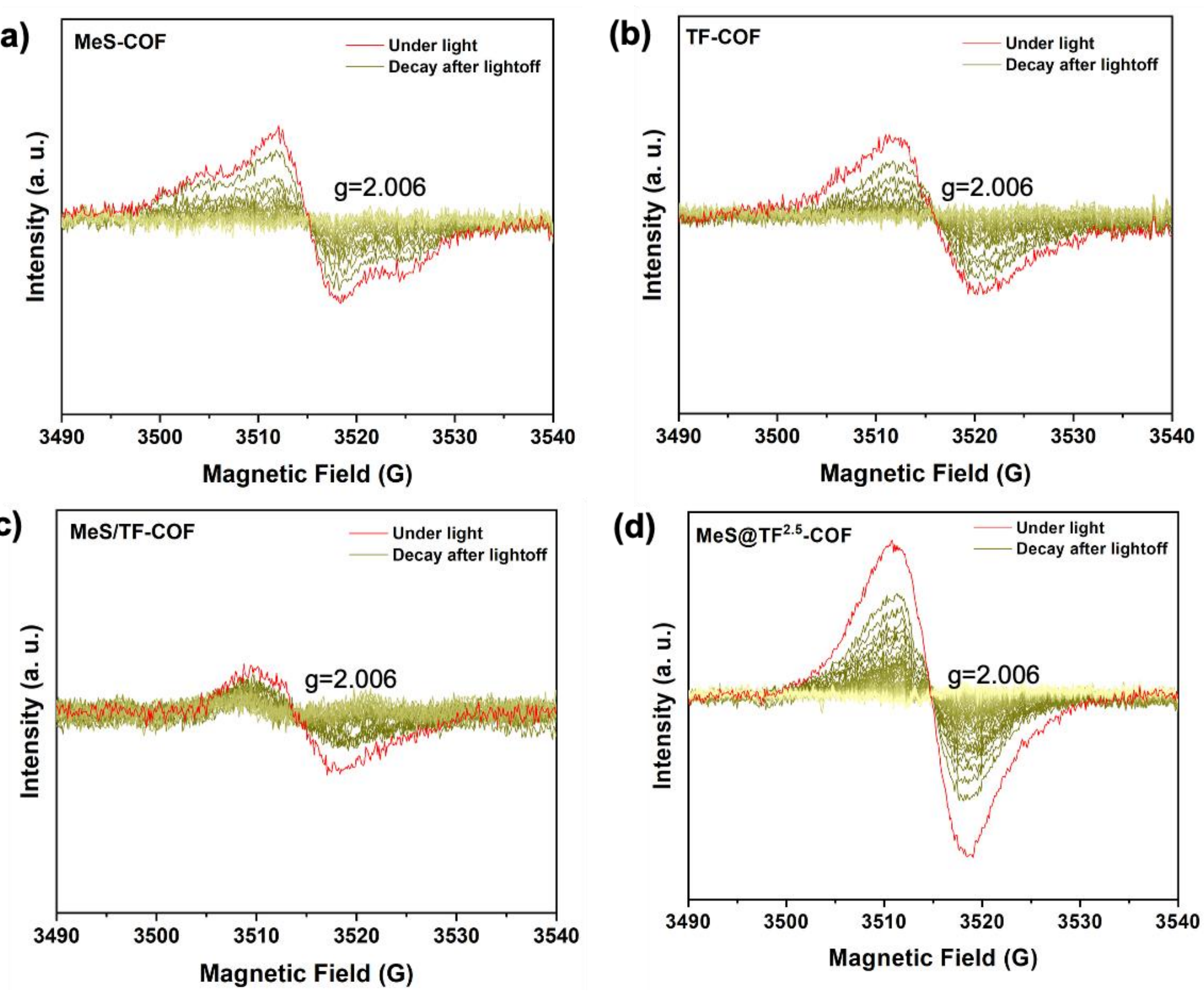

Figure S43 EPR spectra of MeS-COF, TF-COF, MeS/TF-COF and MeS@TF$^{2.5}$-COF under illumination and subsequent signal decay post-irradiation with the initial dark signal subtracted.

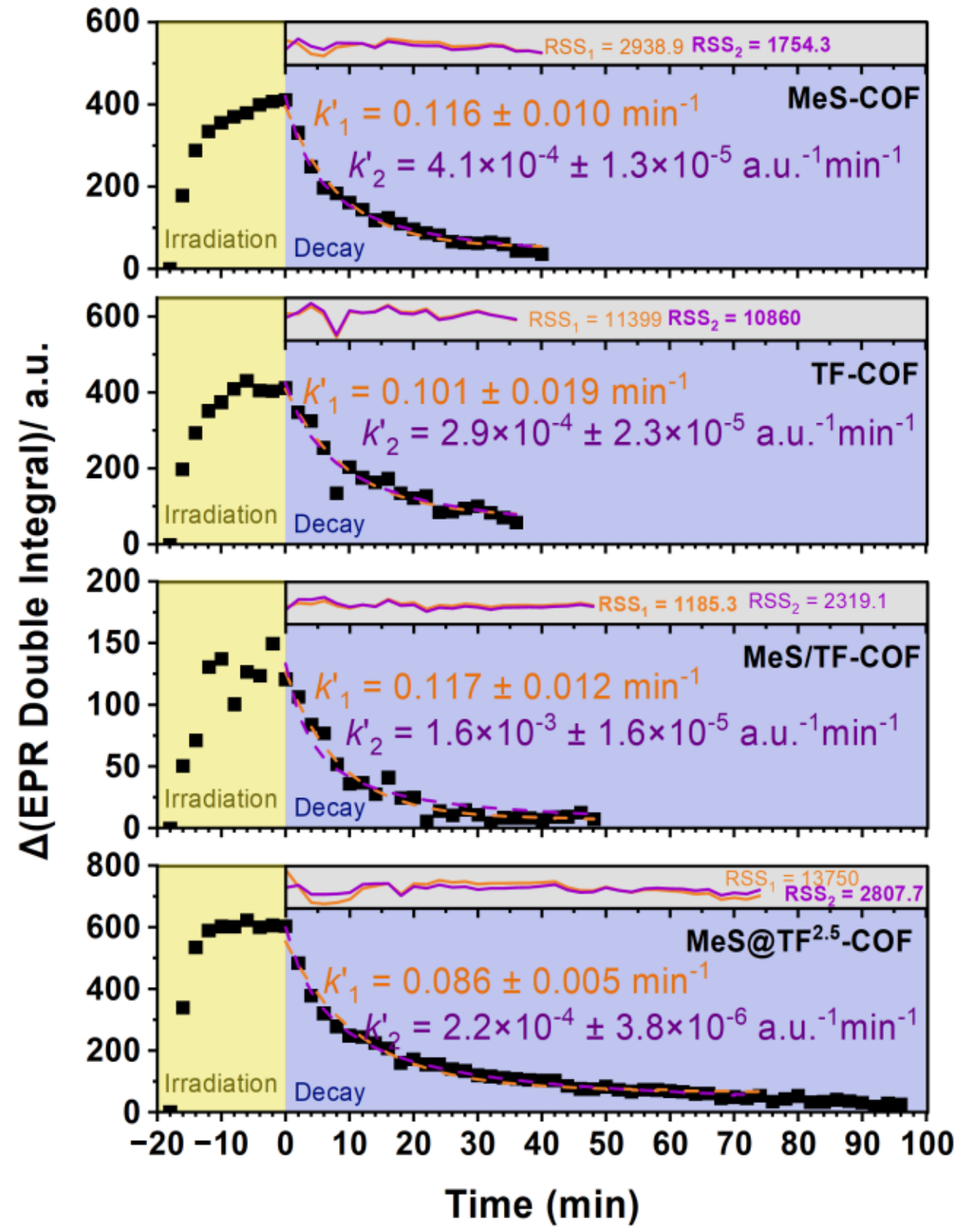

Figure S44 Time-resolved decay profiles obtained by first-order fit and second-order fit of double integration of EPR signals of (a) MeS-COF, (b) TF-COF, (c) MeS/TF-COF and (d) MeS@TF$^{2.5}$-COF.

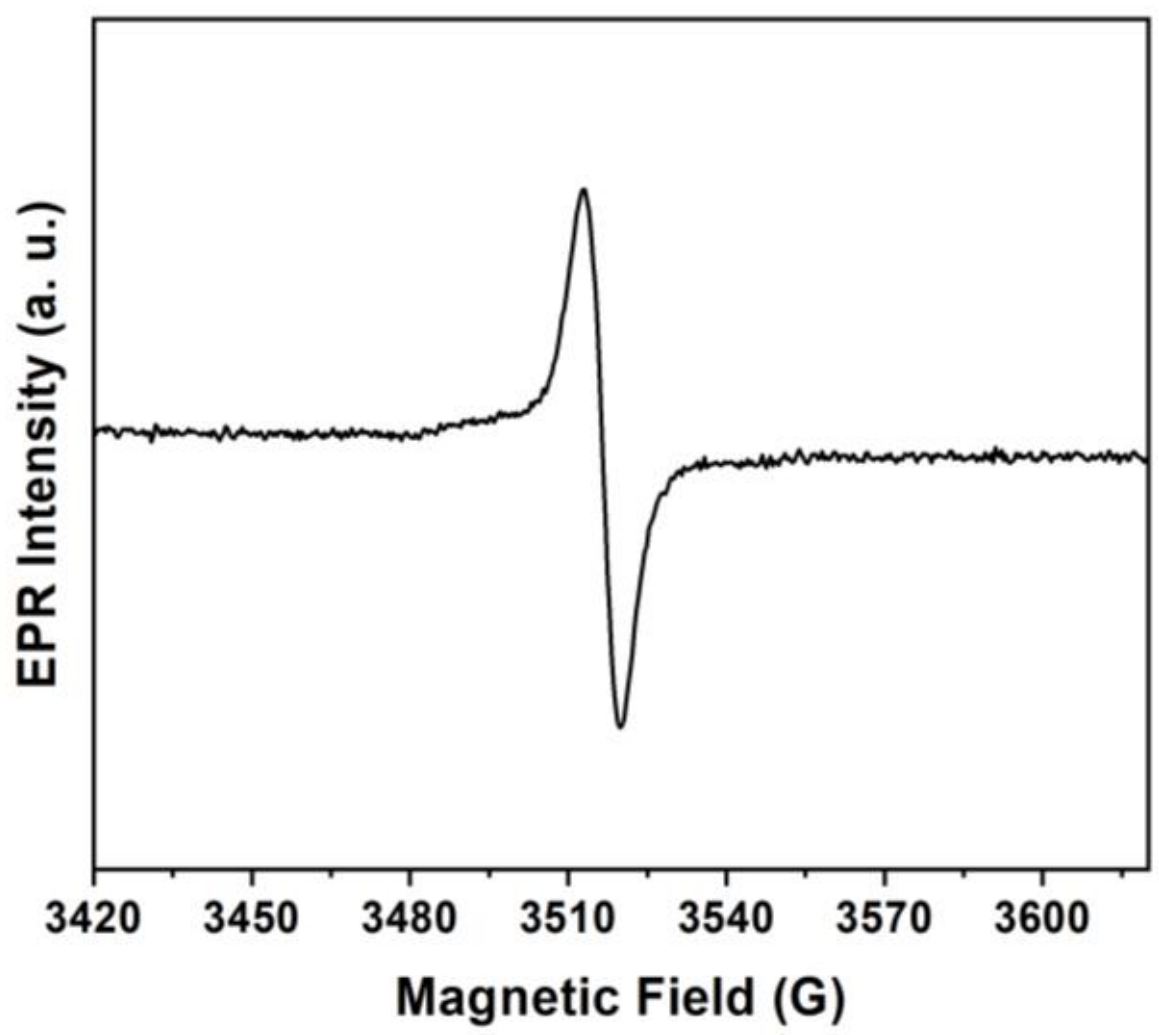

Figure S45 EPR spectrum of the commercial (95% purity) TAPB monomer. The signal likely originates from the air oxidation of the amino groups.

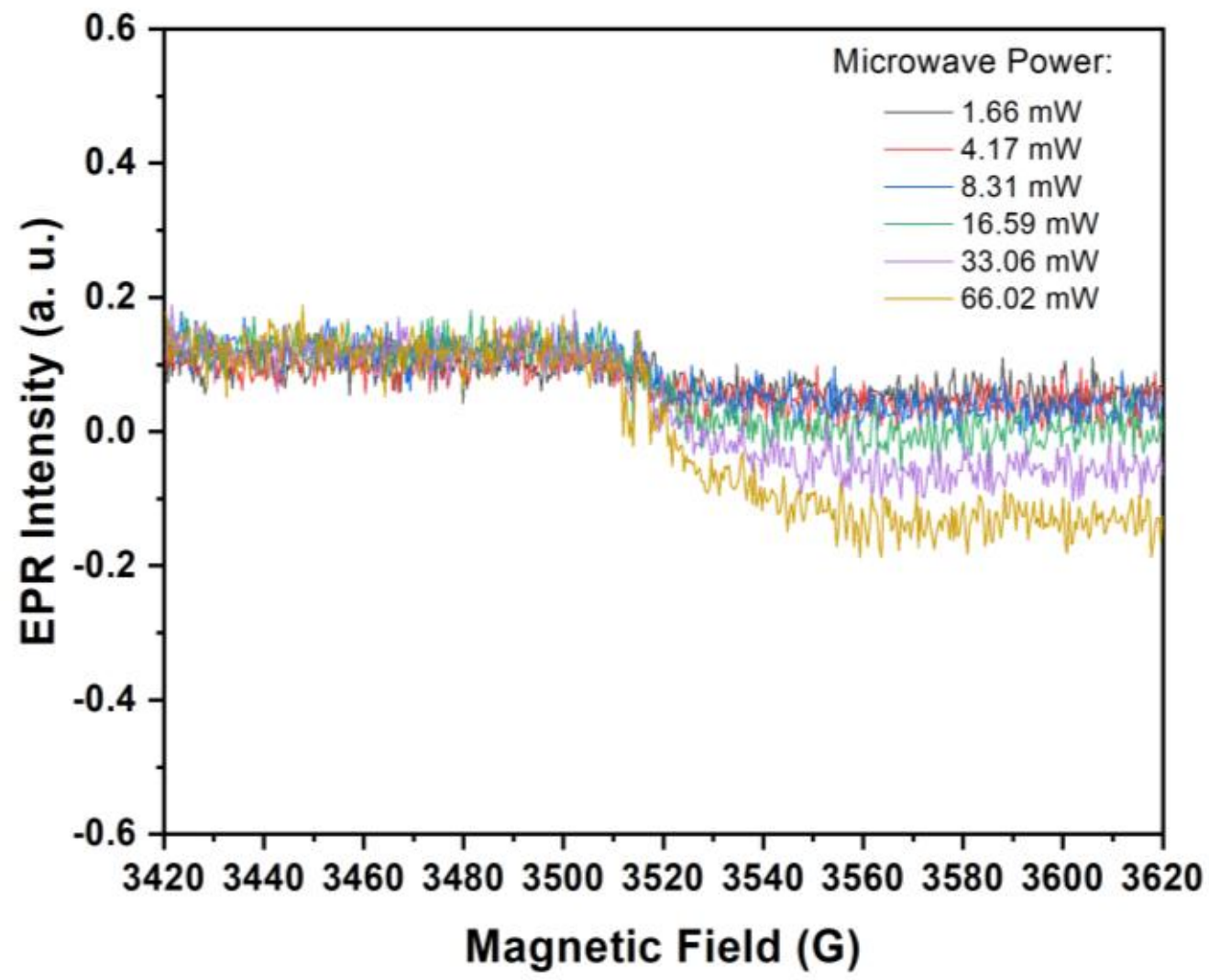

Figure S46 EPR spectrum of DCl-COF in the dark measured with different microwave power.

9. Photocatalytic CO_2 Reduction Experiments

$[Co(bpy)_3]Cl_2$ Complex: Cobalt(II) chloride hexahydrate ($CoCl_2 \cdot 6H_2O$, 118.9 mg, 0.5 mmol) and 2,2′-bipyridine (234.3 mg, 1.5 mmol) were dissolved in 10 mL of methanol under a N_2 atmosphere. The resulting pink solution was stirred at room temperature for 30 minutes in the dark. The reaction mixture was then stored under N_2 and in the dark until further use.

Synthesis of Co-doped COFs: The COF nanoparticles (5 mg) were dispersed in the above-described methanol solution of $[Co(bpy)_3]Cl_2$ complex (5 mL) under a N_2 atmosphere. The suspension was stirred at room temperature for 5 hours and the solid product was collected by centrifugation, washed twice with methanol, and dispersed in acetonitrile for subsequent photocatalytic studies.

Characterization of Co content in Co-doped COFs: The Co content in the COFs was determined by Inductively Coupled Plasma Optical Emission Spectrometry (ICP-OES, Agilent Technologies, 5100). A 1000 ppm Co standard solution was diluted by 5% aqueous nitric acid solution (HNO_3) to prepare a series of 10 mL calibration solutions with the concentration of 0.05, 0.1, 0.2, 0.5, 1, 2, 3 ppm. Then, the 1.2 mg of Co-doped MeS-COF, 2 mg of MeS/TF-COF and 1mg of MeS@$TF^{2.5}$-COF were dissolved in a 10 mL of 5% HNO_3 with 1 mL of H_2O_2 for ICP-OES measurement. The measured Co concentration of Co-loaded MeS-COF, MeS/TF-COF and MeS@$TF^{2.5}$-COF were 0.34, 0.72 and 0.32 ppm, corresponding to Co loading of 0.32 wt%, 0.39 wt% and 0.35 wt%.

Photocatalytic CO_2 Reduction: The COF/$[Co(bpy)_3]Cl_2$ composite (1 mg) was dispersed in a mixed solvent of acetonitrile and water (4 mL, v/v = 4:1) in a 50 mL sealed quartz reactor (see below). The suspension was purged with N_2 for 30 min, followed by CO_2 bubbling for another 30 min to achieve CO_2 saturation. The reaction mixture was subsequently irradiated with a 300 W Xe lamp (320-780 nm wavelength, average light intensity: 835 mW cm^{-2}). During illumination, the reactor was magnetically stirred.

The gaseous products were sampled using a gas-tight syringe (1mL) and analyzed by gas chromatography (Agilent, 8890 GC system). The average CO production rate was calculated by dividing the total amount of CO produced after 5 h of reaction by the reaction time (5 h). Liquid products were detected by 1H NMR using reaction mixture (500 µL). Benzaldehyde (10 µL, 52.8 mM) was added as internal standard to determine the amount of liquid products. The photocatalytic production rate of HCOOH was calculated to be 1.17 mmol g^{-1} h^{-1}.

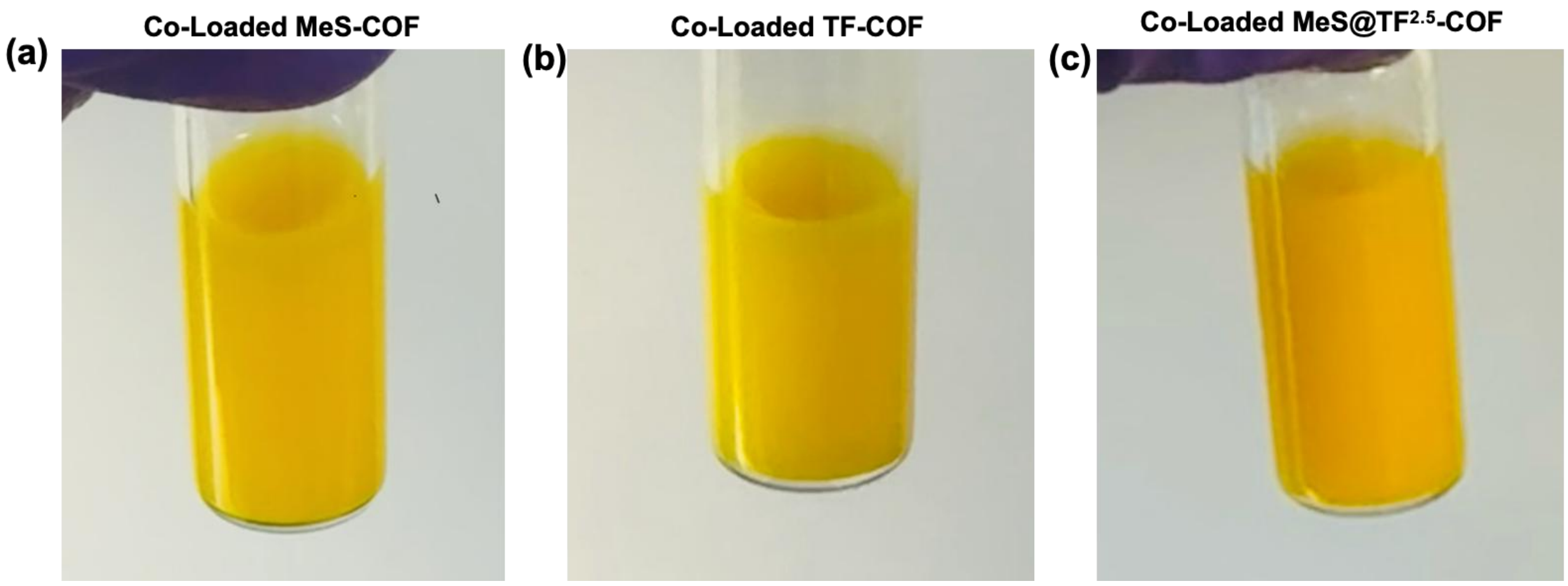

Figure S47 Photographs of Co-loaded (a) MeS-COF, (b) TF-COF, and (c) MeS@TF$^{2.5}$-COF stored in MeCN solvent for approximately 6 months.

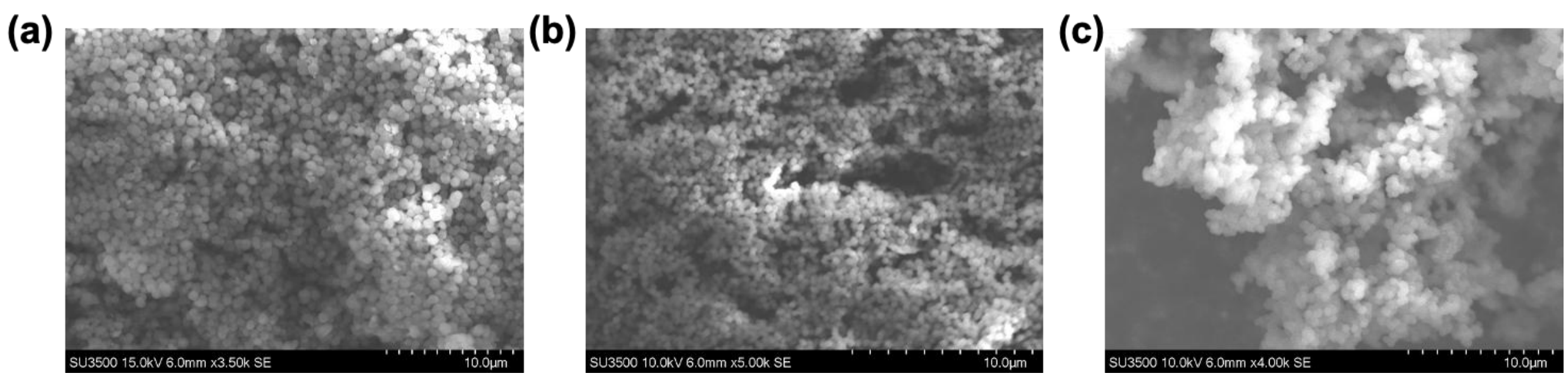

Figure S48 SEM images of Co-loaded (a) MeS-COF, (b) TF-COF, and (c) MeS@TF$^{2.5}$-COF after stored in MeCN solvent for approximately 6 months.

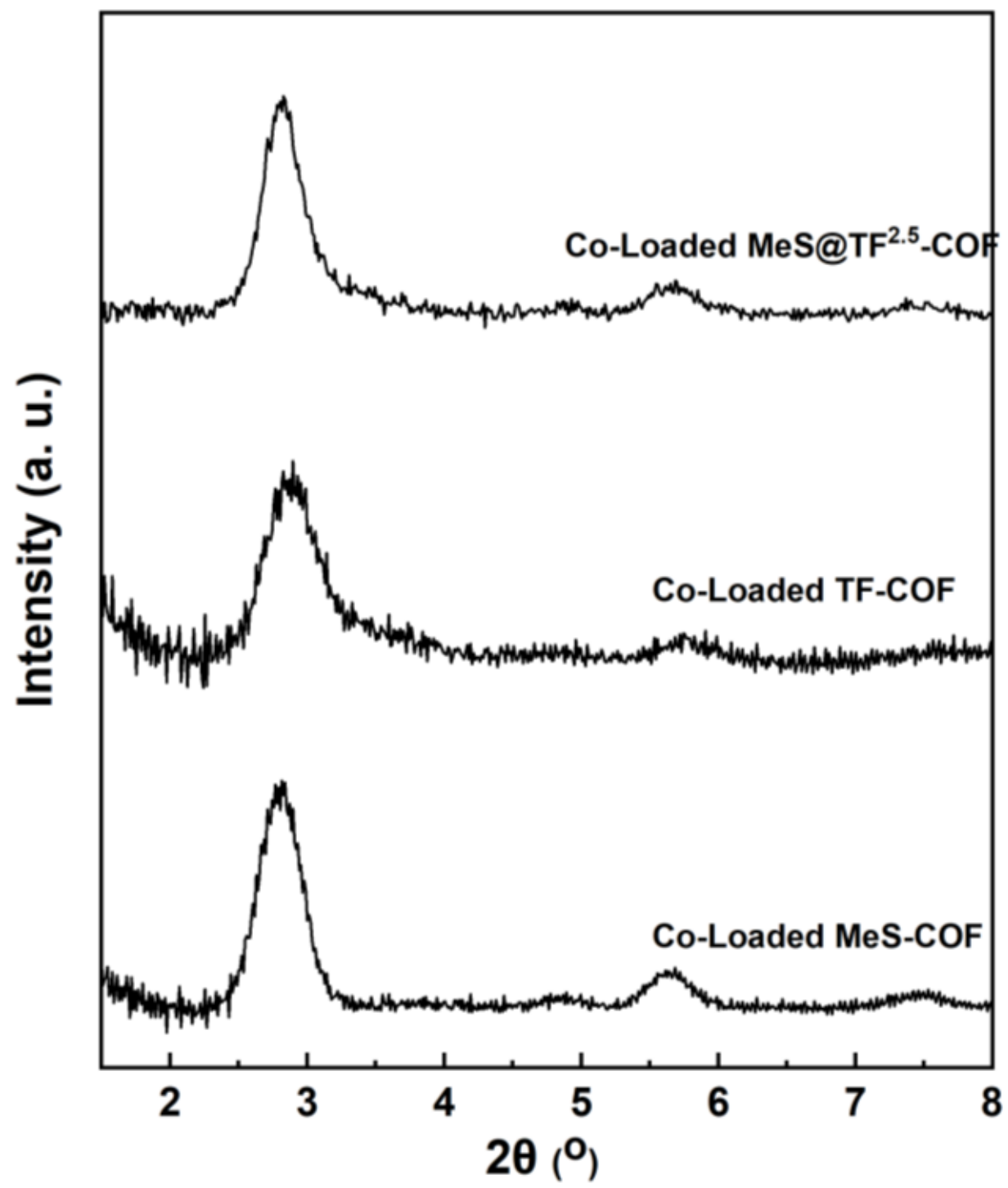

Figure S49 SAXS pattern of Co-loaded (a) MeS-COF, (b) TF-COF, and (c) MeS@TF$^{2.5}$-COF after stored in MeCN solvent for approximately 6 months.

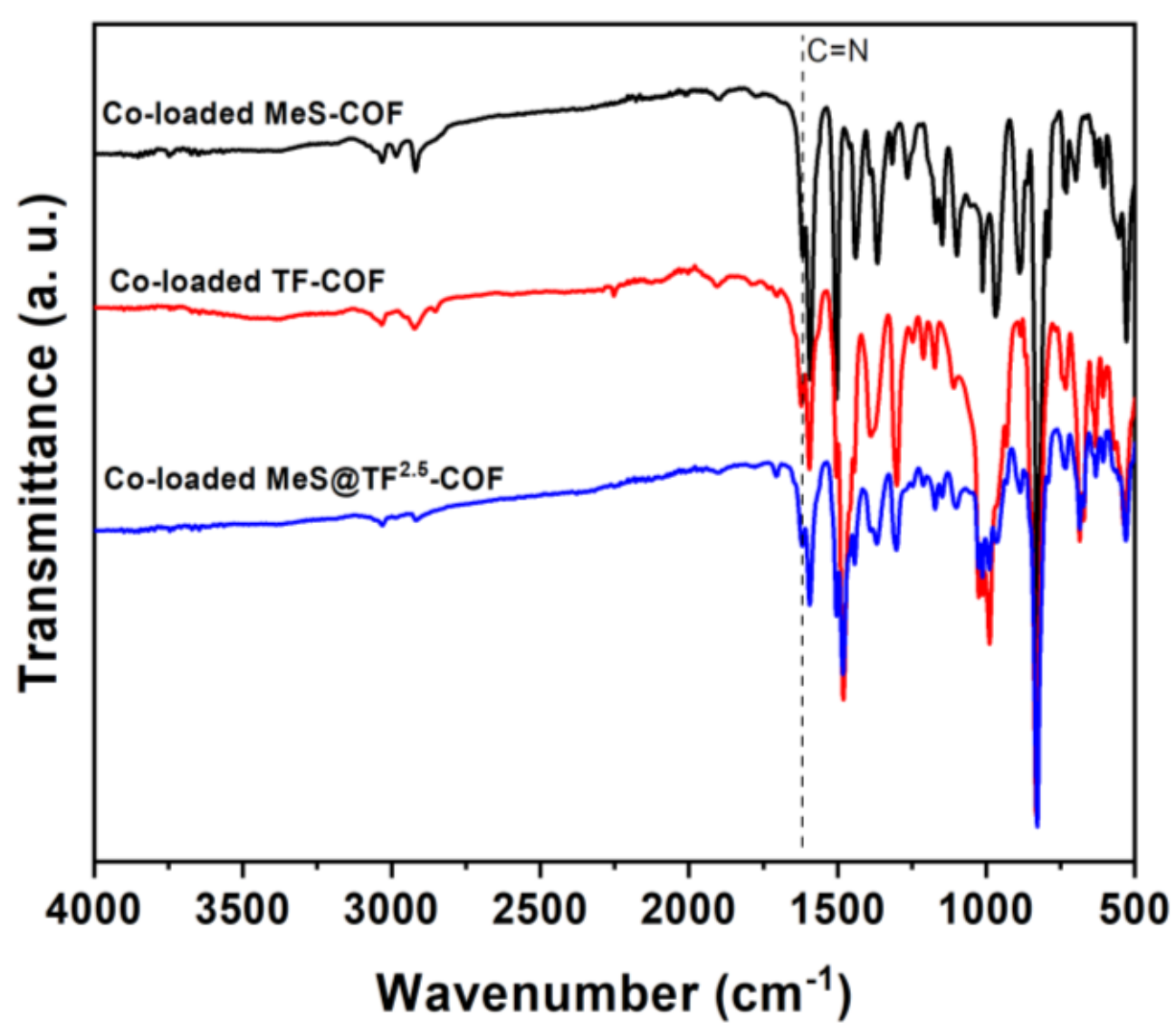

Figure S50 FTIR spectra of Co-loaded MeS-COF, Co-loaded TF-COF, and Co-loaded MeS@TF$^{2.5}$-COF after stored in MeCN solvent for approximately 6 months.

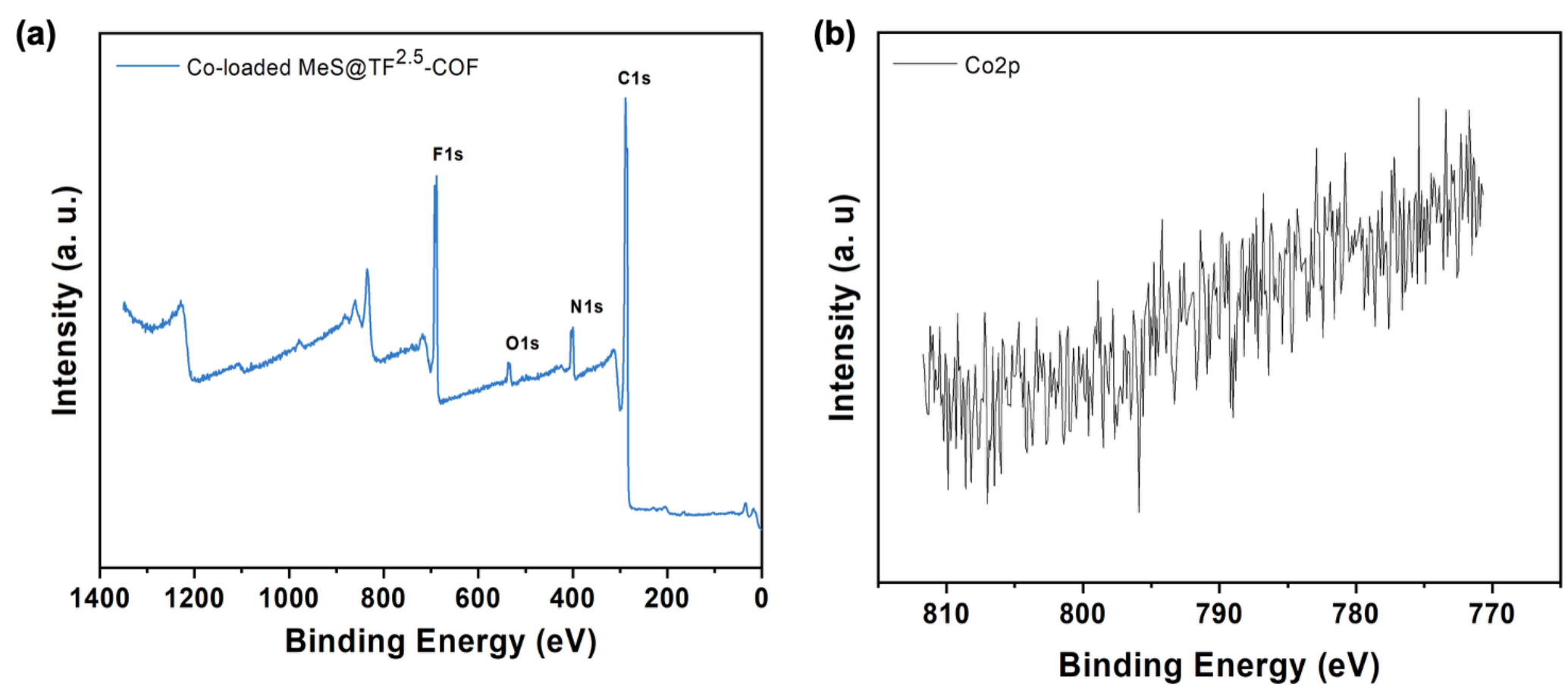

Figure S51 XPS (a) survey and Co2p spectra of Co-loaded MeS@TF$^{2.5}$-COF. The amount of Co on the surface of COF nanoparticles is below the detection limit (<0.1%)

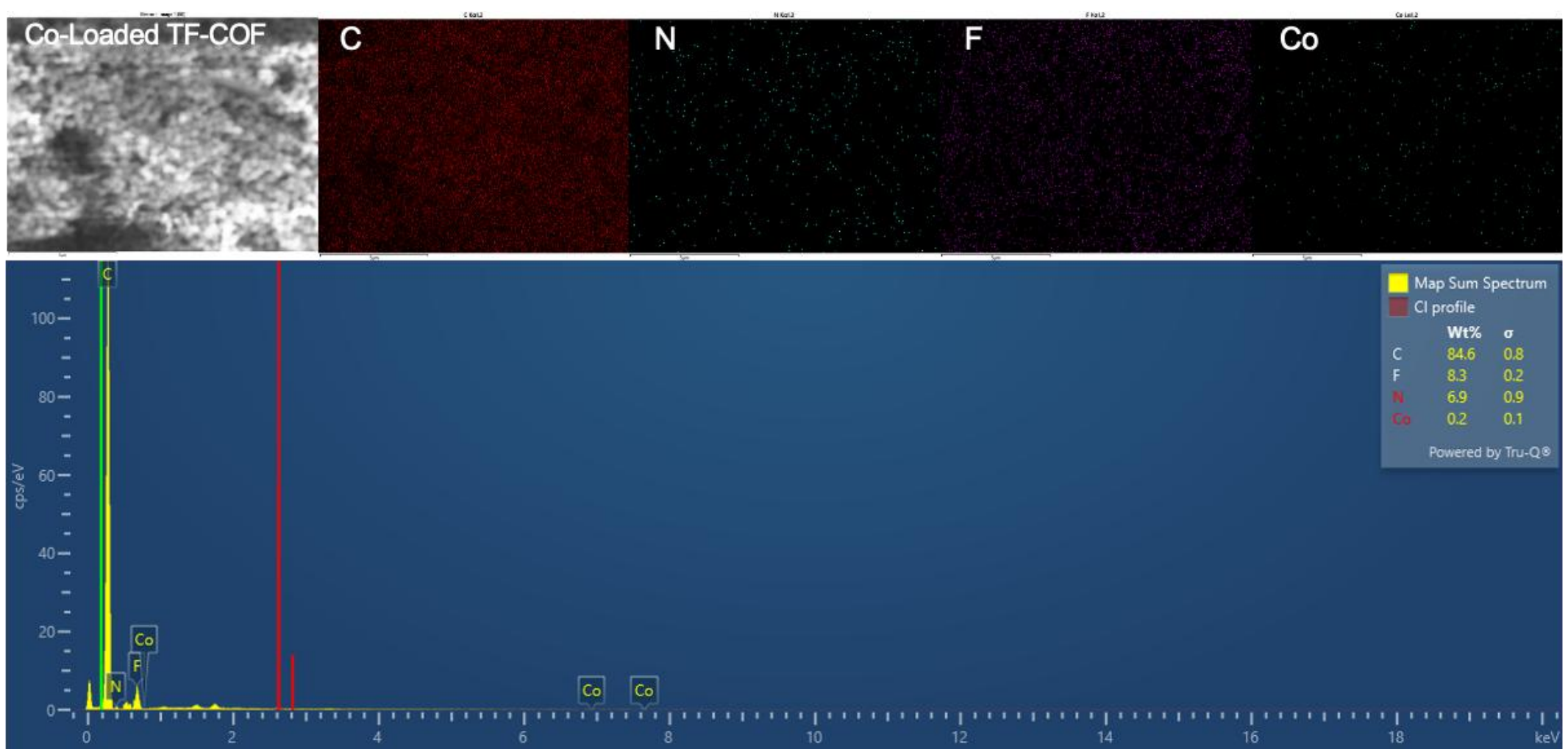

Figure S52 SEM-EDX mapping of Co-loaded TF-COF.

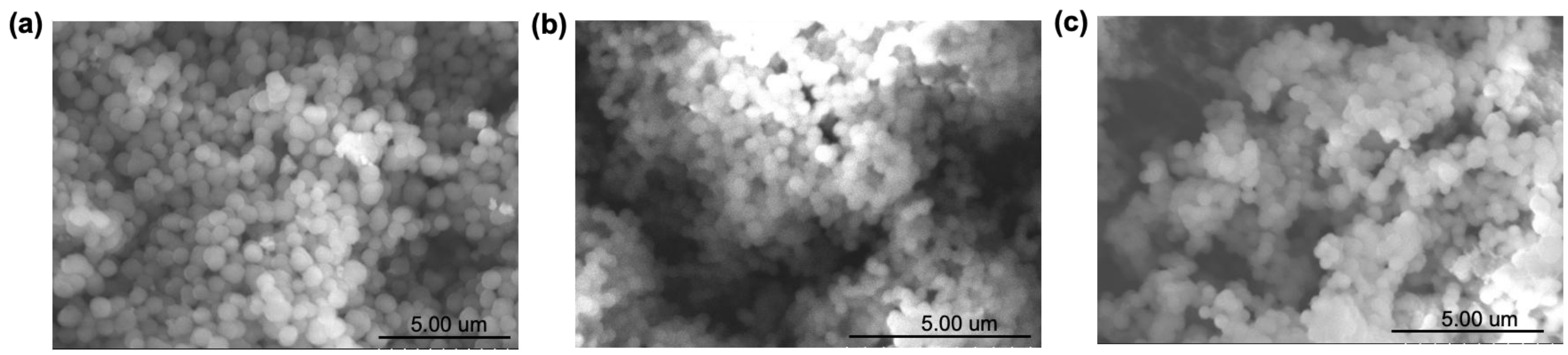

Figure S53 SEM images of Co-loaded (a) MeS-COF, (b) TF-COF, and (c) MeS@TF$^{2.5}$-COF after 5h of photocatalysis.

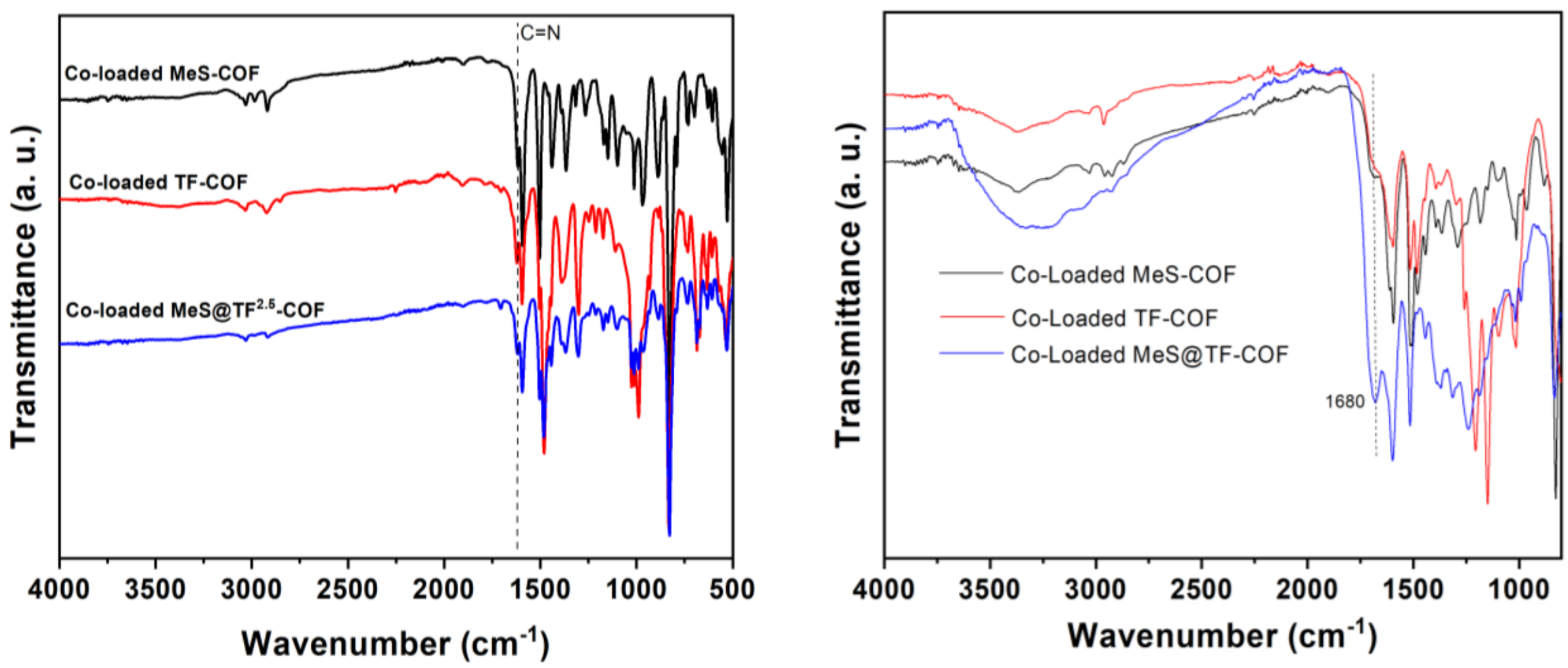

Figure S54 FTIR spectra of Co-loaded COFs before (left) and after (right) 5h of photocatalysis.

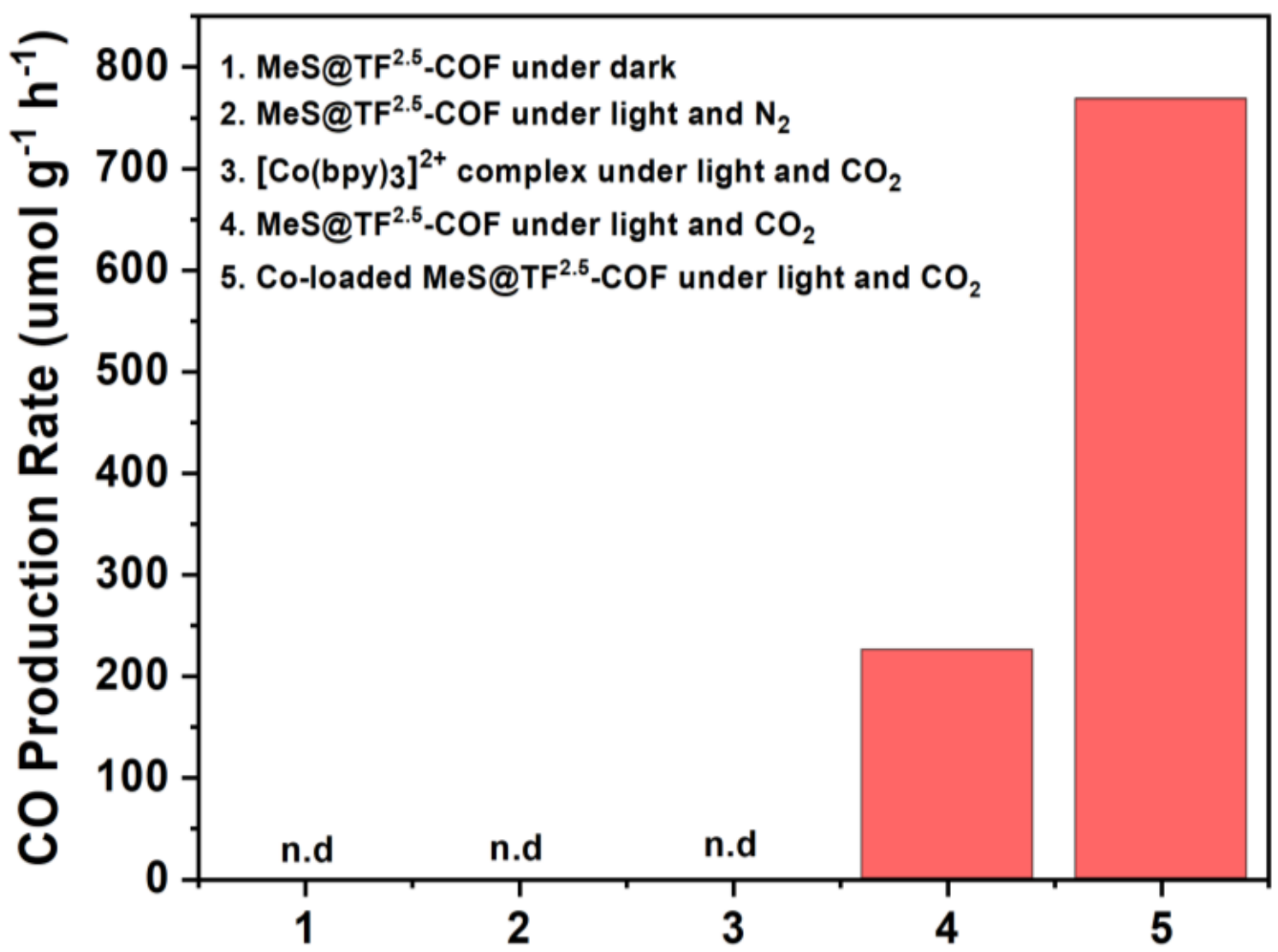

Figure S55 Control experiments with MeS@TF$^{2.5}$-COF catalyst showing CO formation under various reaction conditions.

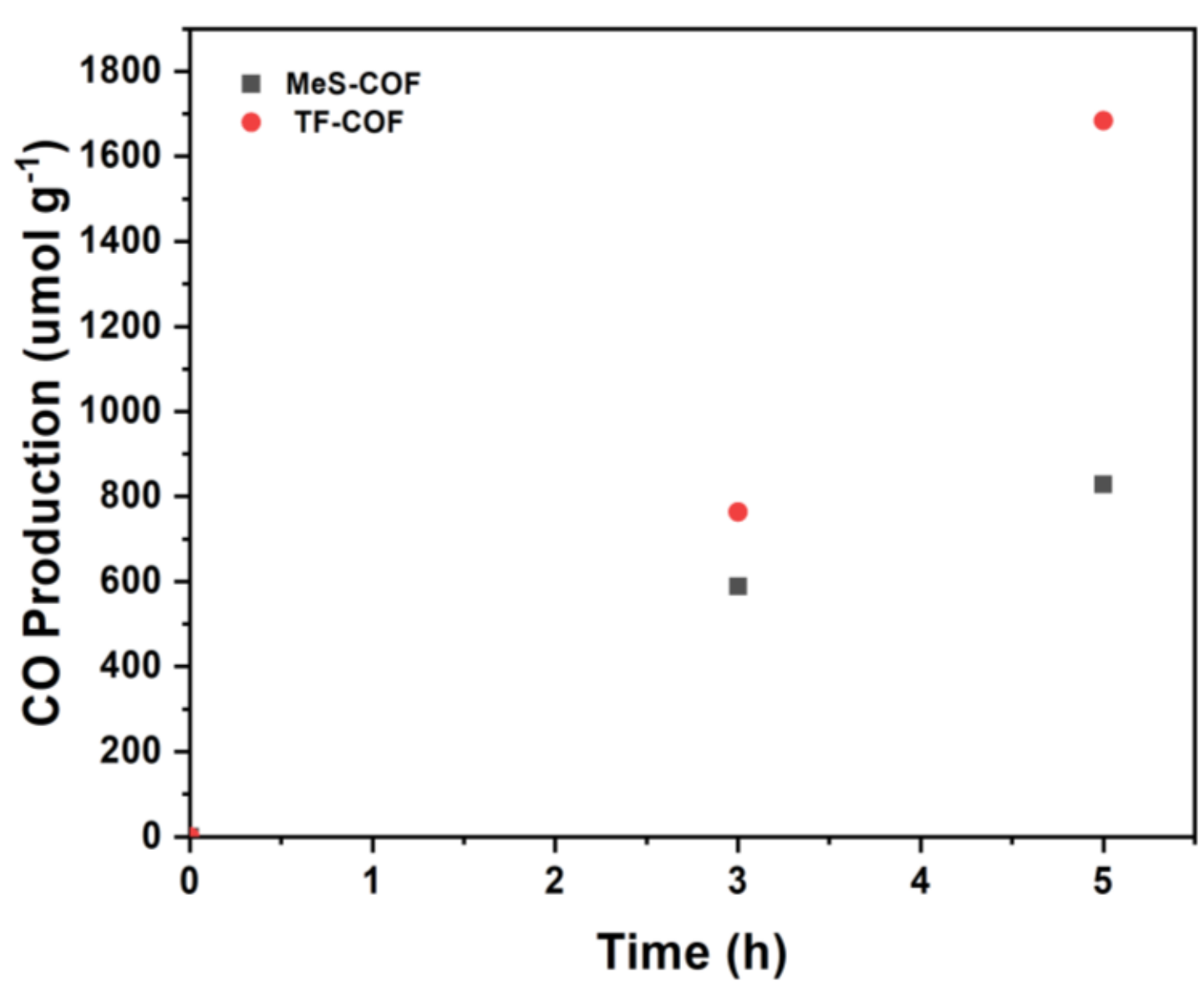

Figure S56 Irradiation time-dependent CO production of (cobalt-doped) MeS-COF and TF-COF.

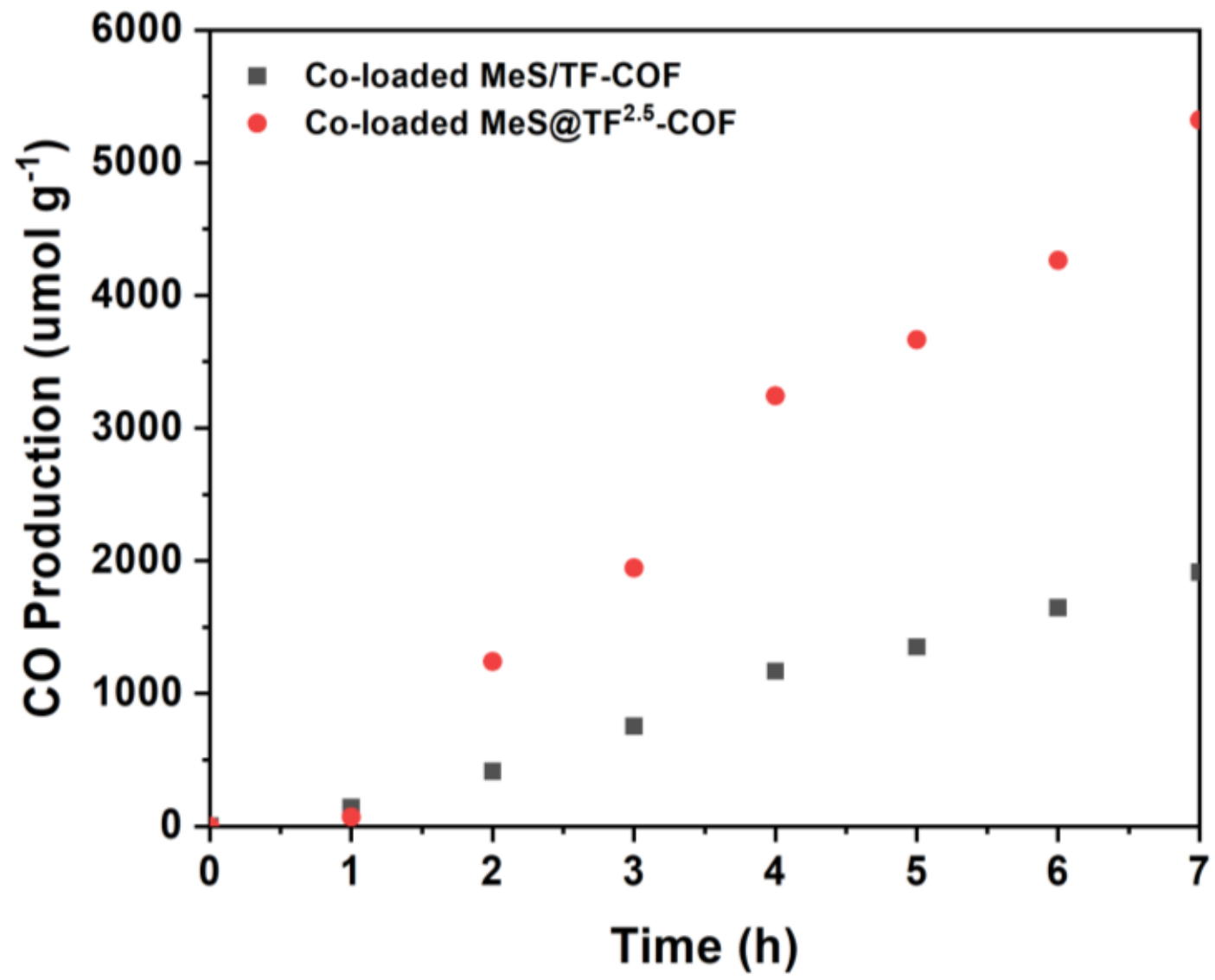

Figure S57 Irradiation time-dependent CO production of Co-loaded MeS/TF-COF and Co-loaded MeS@TF$^{2.5}$-COF.

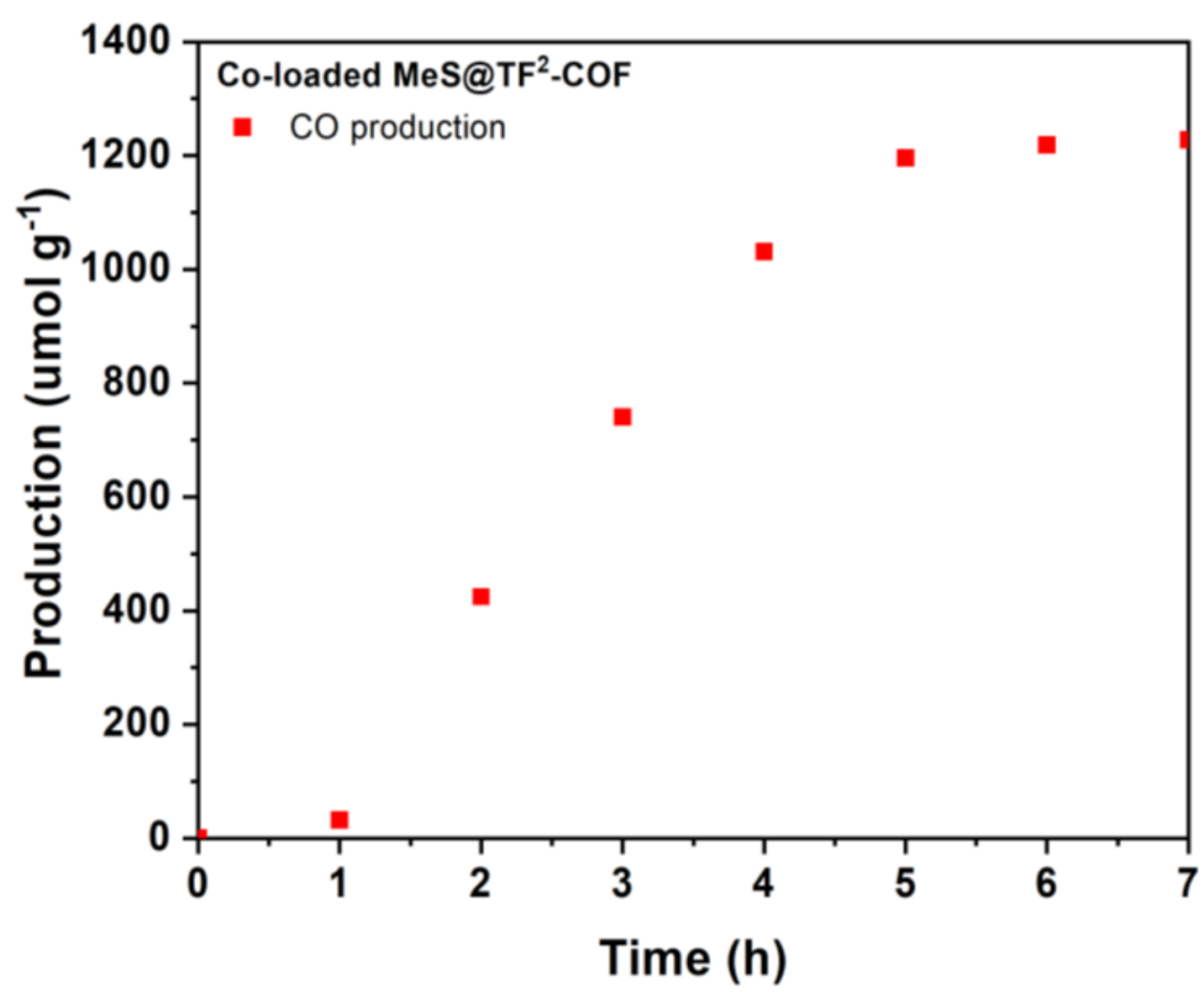

Figure S58 Irradiation time-dependent photocatalytic production of Co-loaded MeS@TF^{2}-COF.

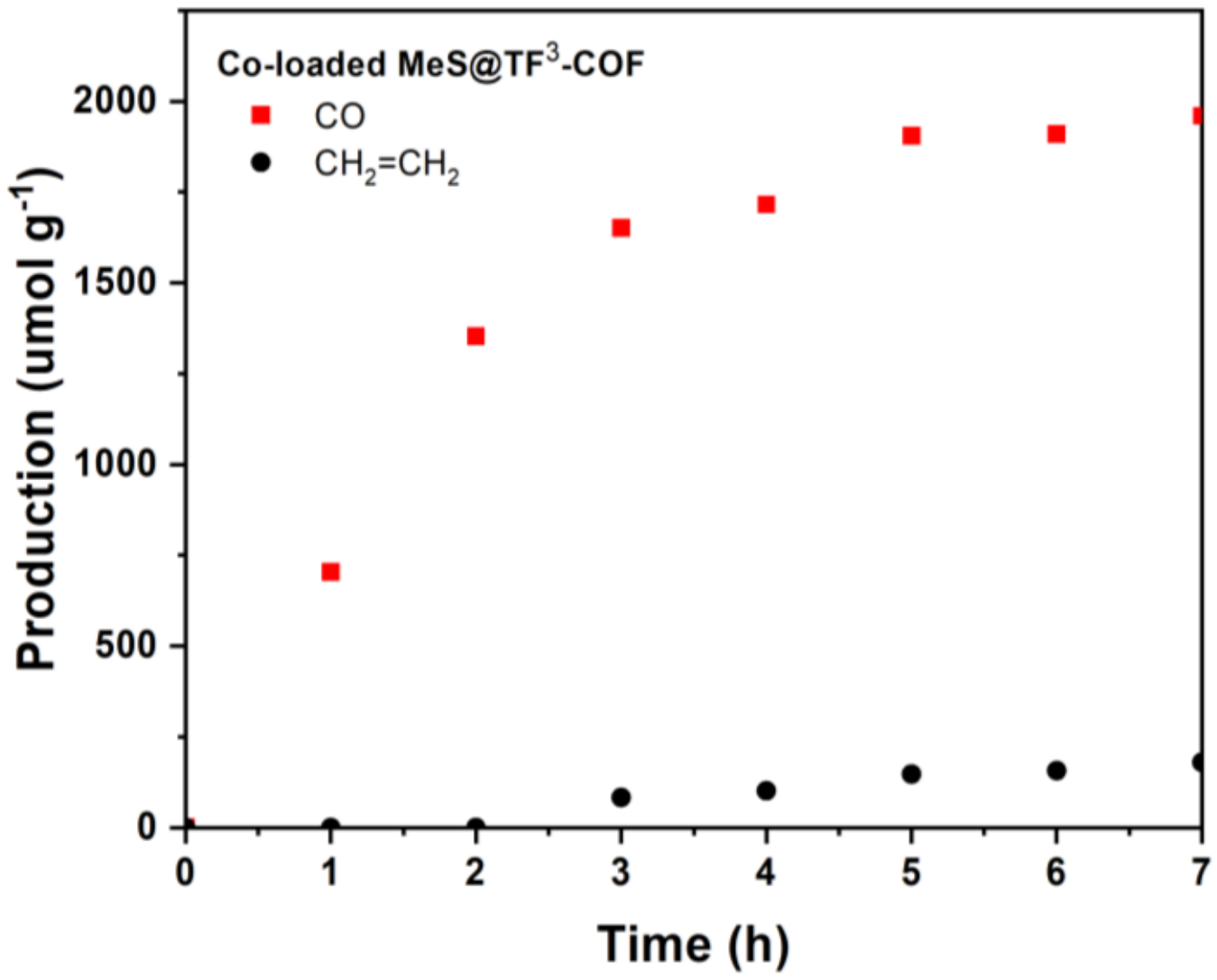

Figure S59 Irradiation time-dependent photocatalytic production of Co-loaded MeS@TF^3-COF. The formation of a small amount of ethylene (0.15 mmol g^{-1} h^{-1}) was observed for this COF with the thickest shell (75 nm).

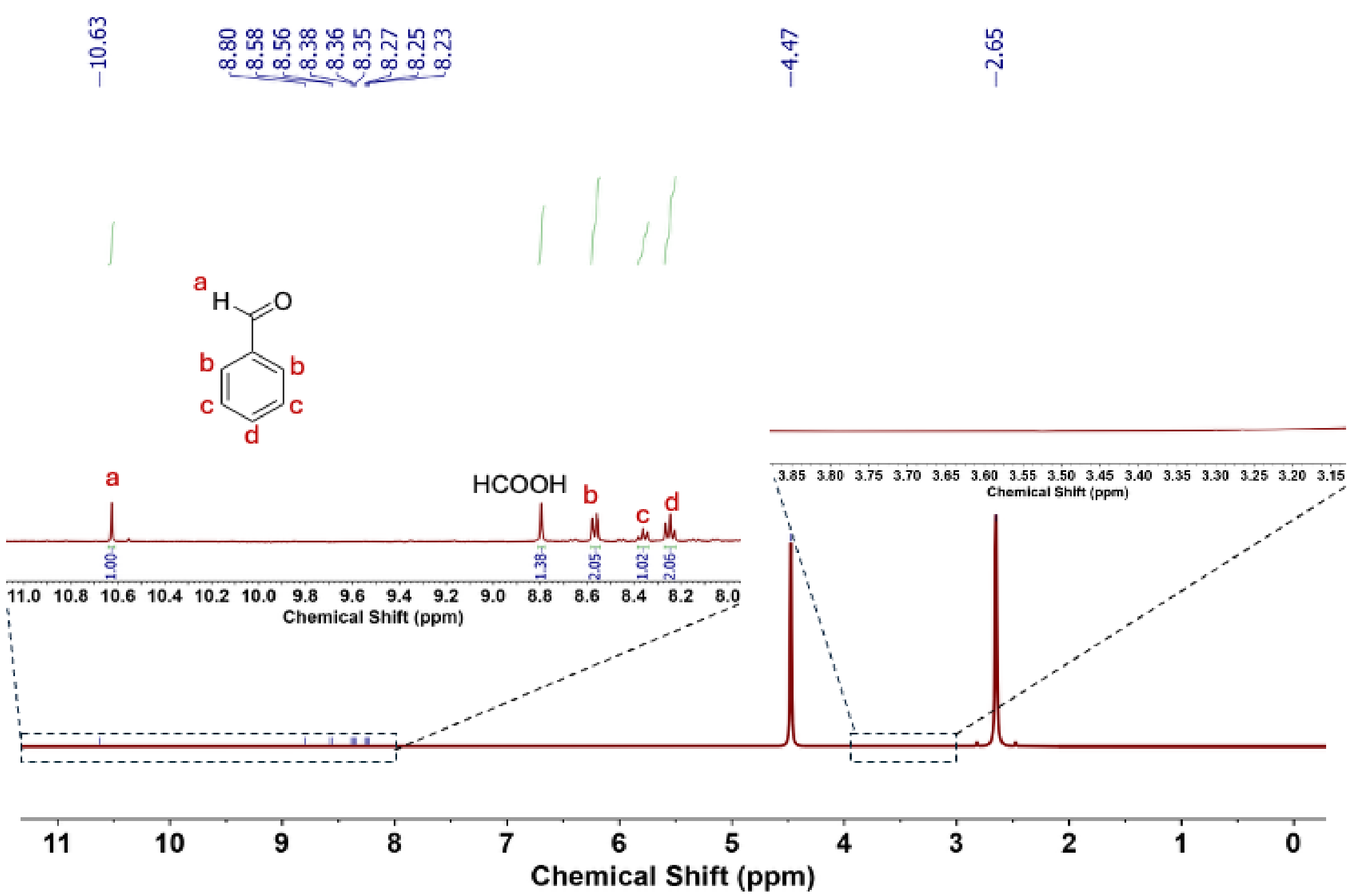

Figure S60 ^{1}H NMR spectrum of the CO_2 photoreduction solution containing the Co-loaded MeS@TF$^{2.5}$-COF in MeCN/H_2O after 5h of irradiation; benzaldehyde was added as an internal standard. The blown-up regions show the formation of HCOOH and no observable quantities of CH_3OH.

Table S13 Summary of photocatalytic CO_2 to CO performance of metal-free COFs without sacrificial agent.

Type	COF sample	Light	Co-catalyst	Sacrificial agent	CO Production Rate (umol g^{-1} h^{-1})	Ref.
Imine	Tz-TTA-COF	> 420 nm 300W Xe lamp	no	no	82	[5]
Imine	CT-COF	> 420 nm 300W Xe lamp	no	no	102.7	[6]
Imine	TAPBB-COF	200-1000nm Xe lamp	no	no	24.6	[7]
Imine	TpBb-COF	> 420 nm White LED light	no	no	89.9	[8]
Imine	BTT-bpy-COF	> 420 nm 300W Xe lamp	no	no	45.9	[9]
Alkyne	TPE-PT	Full spectrum 300W Xe lamp	no	no	10.6	[10]
Imine	2N-COF Membrane	> 420 nm 300W Xe lamp	no	no	310	[11]
Imine	2N-COF Powder	> 420 nm 300W Xe lamp	no	no	98	[11]
Imine	En-Q1DCOF	> 420 nm 300W Xe lamp	no	no	137.6	[12]
Sp2-carbon	BTE-TBD-COF	320-780 nm 300W Xe lamp	no	no	382.0	[13]
Sp2-carbon	NKCOF-63	> 420 nm 300W Xe lamp	no	no	347.1	[14]
Sp2-carbon	NKCOF-64	> 420 nm 300W Xe lamp	no	no	403.0	[14]
Sp2-carbon	NKCOF-65	> 420 nm 300W Xe lamp	no	no	139.2	[14]
Imine	MeS@TF-COF	320-780 nm 300W Xe lamp	no	no	226	This work

Table S14 Summary of photocatalytic CO_2 to CO performance of metal-containing COFs without sacrificial agent.

Type	COF sample	Light	Co-catalyst	Sacrificial agent	CO Production Rate (umol g^{-1} h^{-1})	Ref.
Imine	TT-COF-Zn	420-800 nm<br>300W Xe lamp	no	no	2.1	[15]
Olefine	viCOF-bpy-Re	420-800 nm<br>300W Xe lamp	no	no	190.6	[16]
Imine	TCOF-$MnMo_6$	400-800 nm<br>300W Xe lamp	no	no	37.2	[17]
Sp2-carbon	NKCOF-65-Re	> 420 nm<br>300W Xe lamp	no	no	1464.0	[14]
Imine	TpBpy-COF-Mo	> 420 nm<br>300W Xe lamp	no	no	6.2	[18]
Imine	Cu_4COF	300W Xe lamp	no	no	23.8	[19]
Dioxin	COF-318-TiO_2	320-800 nm<br>300W Xe lamp	no	no	69.7	[20]
Imine	MeS@TF-COF	320-780 nm<br>300W Xe lamp	$[Co(bpy)_3]Cl_2$	no	733	This work

10. Reference

[1] C. Feriante, A. M. Evans, S. Jhulki et al. "New mechanistic insights into the formation of imine-linked two-dimensional covalent organic frameworks," *Journal of the American Chemical Society* 142(2020):18637-18644.

[2] R. L. Li, N. C. Flanders, A. M. Evans, et al., "Controlled growth of imine-linked two-dimensional covalent organic framework nanoparticles," *Chemical Science* 10 (2019): 3796-3801.

[3] H. Gommans, S. Schols, A. Kadashchuk, P. Heremans, S. Meskers, "Exciton diffusion length and lifetime in subphthalocyanine films," *The Journal of Physical Chemistry C* 113 (2009): 2974-2979.

[4] S. Chandrasekhar, "Stochastic problems in physics and astronomy," *Reviews of Modern Physics* 15 (1943): 1.

[5] S. Biswas, A. Dey, F. A. Rahimi, S. Barman, T. K. Maji, "Metal-free highly stable and crystalline covalent organic nanosheet for visible-light-driven selective solar fuel production in aqueous medium," *ACS Catalysis* 13 (2023): 5926-5937.

[6] K. Lei, D. Wang, L. Ye, et al., "A Metal-Free Donor–acceptor covalent organic framework photocatalyst for visible-light-driven reduction of CO_2 with H_2O," *ChemSusChem* 13 (2020): 1725-1729.

[7] L. j. Wang, R. l. Wang, X. Zhang, J. l. Mu, Z. y. Zhou, Z. m. Su, "Improved photoreduction of CO_2 with water by tuning the valence band of covalent organic frameworks," *ChemSusChem* 13 (2020): 2973-2980.

[8] J.-X. Cui, L.-J. Wang, L. Feng, et al., "A metal-free covalent organic framework as a photocatalyst for CO_2 reduction at low CO_2 concentration in a gas–solid system," *Journal of Materials Chemistry A* 9 (2021): 24895-24902.

[9] H. Dong, H.-T. Che, L.-W. Bai, et al., "Benzotrithiophene-based covalent organic frameworks with rhenium modified for artificial photosynthetic CO_2 reduction," *Inorganic Chemistry* 63 (2024): 24421-24428.

[10] W. Zou, Y. Cheng, Y. X. Ye, X. Wei, Q. Tong, L. Dong, G. Ouyang, "Metal-free photocatalytic CO_2 reduction to CH_4 and H_2O_2 under non-sacrificial ambient conditions," *Angewandte Chemie International Edition* 135 (2023): e202313392.

[11] S. Gao, Q. Zhang, X. Su, et al., "Ingenious artificial leaf based on covalent organic framework membranes for boosting CO_2 photoreduction," *Journal of the American Chemical Society* 145 (2023): 9520-9529.
[12] J. Bai, Y. Hu, F. Si, et al., "Quasi-one-dimensional enaminone-linked covalent organic frameworks for efficient CO_2 photoreduction," *Nature Communications* 17 (2026): 2158.
[13] L. Yang, W. Yan, N. Yang, et al., "Regulating π-Conjugation in sp2-Carbon-Linked Covalent Organic Frameworks for Efficient Metal-Free CO_2 Photoreduction with H_2O," *Small* 19 (2023): 2208118.
[14] Y. Zhang, Z. Liang, K. Wang, et al., "Microenvironment Engineering Vinylene-Linked Covalent Organic Frameworks for Highly Efficient CO2 Photoreduction," *Journal of the American Chemical Society* 148 (2026): 16107-16118.
[15] M. Lu, J. Liu, Q. Li, et al., "Rational design of crystalline covalent organic frameworks for efficient CO_2 photoreduction with H_2O," *Angewandte Chemie International Edition* 131 (2019): 12522-12527.
[16] Y. Z. Cheng, W. Ji, P. Y. Hao, et al., "A fully conjugated covalent organic framework with oxidative and reductive sites for photocatalytic carbon dioxide reduction with water," *Angewandte Chemie International Edition* 62 (2023): e202308523.
[17] M. Lu, M. Zhang, J. Liu, et al., "Confining and highly dispersing single polyoxometalate clusters in covalent organic frameworks by covalent linkages for CO2 photoreduction," *Journal of the American Chemical Society* 144 (2022): 1861-1871.
[18] M. Kou, W. Liu, Y. Wang, et al., "Photocatalytic CO_2 conversion over single-atom MoN_2 sites of covalent organic framework," *Applied Catalysis B: Environmental* 291 (2021): 120146.
[19] Y. Xu, J. P. Dong, L. Wang, et al., "3D Cluster-Based Covalent Organic Framework for Efficient CO_2 Photoreduction," *Angewandte Chemie International Edition* 137 (2025): e202501391.
[20] M. Zhang, M. Lu, Z. L. Lang, et al., "Semiconductor/covalent-organic-framework Z-scheme heterojunctions for artificial photosynthesis," *Angewandte Chemie International Edition* 132 (2020): 6562-6568.
